# Synthesis, Urease Inhibition and Molecular Modelling Studies of Novel Derivatives of the Naturally Occurring *β*-Amyrenone

**DOI:** 10.1007/s13659-018-0193-7

**Published:** 2018-11-28

**Authors:** Jean J. K. Bankeu, Hira Sattar, Yannick S. F. Fongang, Syeda W. Muhammadi, Conrad V. Simoben, Fidele Ntie-Kang, Guy R. T. Feuya, Marthe A. T. Tchuenmogne, Mehreen Lateef, Bruno N. Lenta, Muhammad S. Ali, Augustin S. Ngouela

**Affiliations:** 1 Department of Chemistry, Faculty of Science, The University of Bamenda, P.O. Box 39, Bambili, Cameroon; 2 International Center for Chemical and Biological Sciences, University of Karachi, Karachi, 75270 Pakistan; 3 Department of Chemistry, Higher Teacher Training College, University of Maroua, P.O. Box 55, Maroua, Cameroon; 4 Department of Pharmaceutical Chemistry, Martin-Luther University of Halle-Wittenberg, Wolfgang-Langenbeck-Str. 4, 06120 Halle (Saale), Germany; 5 Department of Chemistry, Faculty of Science, University of Buea, P. O. Box 63, Buea, Cameroon; 6 Department of Chemistry, Faculty of Science, Scientific and Technical University of Masuku, Box 943, Franceville, Gabon; 7 Department of Chemistry, Faculty of Science, University of Yaoundé I, P.O. Box 812, Yaoundé, Cameroon; 8 Multi-Disciplinary Research Laboratory (MDRL), Bahria University Medical and Dental College, Bahria University, Karachi, Pakistan; 9 Department of Chemistry, Higher Teacher Training College, University of Yaoundé I, P.O. Box 47, Yaoundé, Cameroon

**Keywords:** *Helicobacter pylori*, Urease inhibition, Docking, Olean-12-en-3-one derivatives

## Abstract

**Abstract:**

Urease enzyme (UE) has been reported to be a potent virulence factor for *Helicobacter pylori* (HP) bacteria indicated to be responsible for various gastrointestinal diseases. Therefore, the spread of HP, currently regarded by the World Health Organization as a class 1 carcinogen, could be better controlled by targeting UE. It is in this line that we have synthesized three new derivatives (**2**–**4**) of the naturally occurring olean-12-en-3-one (**1**), which was previously isolated from the figs of *Ficus vallis*-*choudae* Delile (Moraceae). Among the synthesized compounds, **3** and **4** contain an indole moiety. Their structures were unambiguously assigned by spectroscopic and spectrometric techniques (1D-NMR, 2D-NMR and MS). The starting material and the synthesized compounds were screened for UE inhibition activity, and showed significant activities with IC_50_ values ranging from 14.5 to 24.6 μM, with compound (**1**) being the most potent as compared to the positive control thiourea (IC_50_ = 21.6 μM). Amongst the synthetic derivatives, compound **4** was the most potent (IC_50_ = 17.9 μM), while the others showed activities close to that of the control. In addition, molecular docking study of target compounds **2**–**4** was performed in an attempt to explore their binding mode for the design of more potent UE inhibitors.

**Graphical Abstract:**

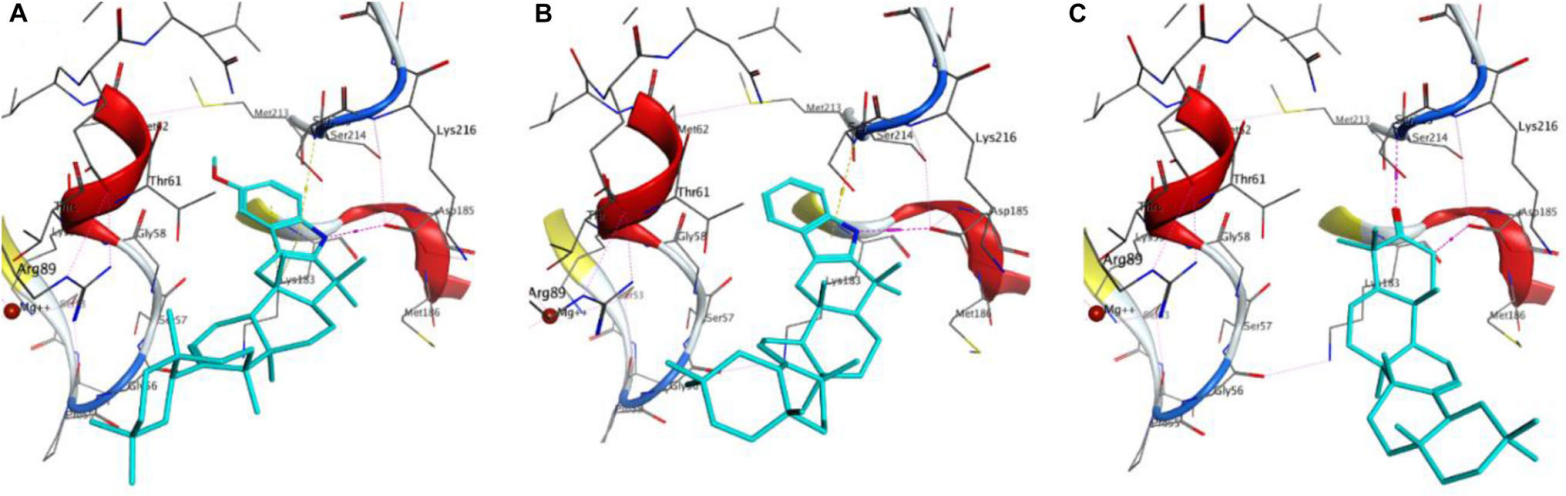

**Electronic supplementary material:**

The online version of this article (10.1007/s13659-018-0193-7) contains supplementary material, which is available to authorized users.

## Introduction

*Helicobacter pylori* (HP) infection is a worldwide problem and the first formally recognized bacterial carcinogen. It is one of the most successful human pathogens, as over half of the world’s population is colonized with this gram-negative bacterium [1, 2]. Unless treated, colonization usually persists lifelong [1, 2]. HP infection represents a key factor in the etiology of various gastrointestinal diseases, ranging from chronic active gastritis without clinical symptoms to peptic ulceration, gastric adenocarcinoma, and gastric mucosa-associated lymphoid tissue lymphoma [1]. The infection has a high morbidity rate, but a low mortality rate but could be treated using currently available antibiotics [2]. Urease enzyme (UE) has been reported to be a potent virulence factor for HP bacteria [3–5]. UE is therefore recognized as a key target in the treatment of HP-related diseases, hence HP UE is an important target for the development of drugs against the parasite. This is because this pathogen requires two nickel-containing enzymes, urease and [NiFe]^−^ hydrogenase, for the efficient colonization of gastric mucosa in humans [6]. Hence UE inhibition in HP could open up a potential pipeline for the development of efficacious next generation antimicrobial drugs targeting the parasite.

Indole alkaloids have been reported from many plant species [7–10]. They have also been reported to play an important role in medicinal chemistry as potential UE inhibitors [7] and antibacterial agents [8]. However, the treatment of HP using the current known synthetic drugs is often associated with high treatment cost, bacterial resistance and adverse side effects related to currently used drugs [11, 12]. Within the framework of exploring natural product as well as their derivatives as a safe source of urease inhibitors, compounds **1**–**4** were tested against urease. Furthermore, molecular docking was employed in order to explore their binding modes and, hence, suggest analogues which could be tighter HP UE binders. The aim of the present work was to design new *β*-amyrenone (**1**) derivatives and screen their biological activity with the quest to identify new compounds with high UE inhibitor activity and potentially low toxicity profiles (Table 1).

**Table 1:** ^13^C NMR Data of *β*-amyrenone (**1**) and synthetic derivatives **2–4**

No.	**1**	**2**	**3**	**4**
	*δ*_C_	*δ*_C_	*δ*_C_	*δ*_C_
1	39.2	38.1	36.9	34.8
2	34.2	20.7	106.9	107.0
3	217.7	167.1	141.9	140.8
4	47.4	42.2	32.5	32.6
5	55.3	55.4	53.1	53.2
6	19.6	19.7	19.3	19.4
7	32.1	32.3	32.1	32.1
8	39.7	39.8	39.9	39.9
9	46.8	46.7	47.3	47.4
10	36.6	36.7	38.0	38.0
11	23.6	23.6	23.6	23.6
12	121.4	121.4	121.9	122.0
13	145.2	145.3	145.0	145.0
14	41.8	41.8	41.9	41.9
15	26.1	26.1	26.1	26.2
16	26.9	26.9	27.0	27.0
17	32.5	32.5	34.0	34.0
18	47.2	47.3	46.3	46.3
19	46.7	46.7	46.8	46.8
20	31.0	31.1	31.1	31.1
21	34.7	34.7	34.7	36.9
22	37.1	37.1	37.1	37.2
23	26.4	28.4	31.0	31.0
24	21.5	24.3	23.7	23.7
25	15.2	14.9	15.8	15.8
26	25.8	25.9	25.8	25.8
27	16.7	16.8	16.6	16.6
28	28.4	28.8	28.5	28.5
29	33.3	33.3	33.4	33.3
30	23.6	23.7	23.4	23.3
1′	–	145.6	–	–
2′	–	128.9	131.1	136.1
3′	–	123.6	110.6	110.3
4′	–	137.5	110.9	120.9
5′	–	129.9	153.7	118.9
6′	–	116.5	100.3	118.0
7′	–	–	128.5	128.2
1″	–	–	56.0	–

## Results and Discussion

### Chemistry

**Scheme 1:**
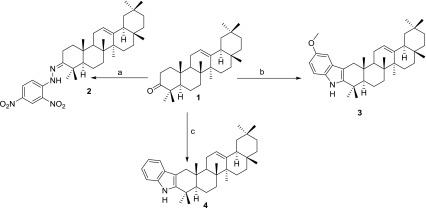
Schematic representation for the synthesis of compounds **2-4**. Reagents: **a** reflux, Dichloromethane (DCM)/MeOH, TFA, 2,4-dinitrophenylhydrazine, 80 °C, 3 days, 81.94% yield; **b** reflux, DCM/MeOH, TFA, *p*-methoxyphenylhydrazine, 110 °C, 6 days, 80.46% yield; **c** reflux, DCM/MeOH, TFA, phenylhydrazine, 230 °C, 8 days, 97.79% yield

**Fig. 1:**
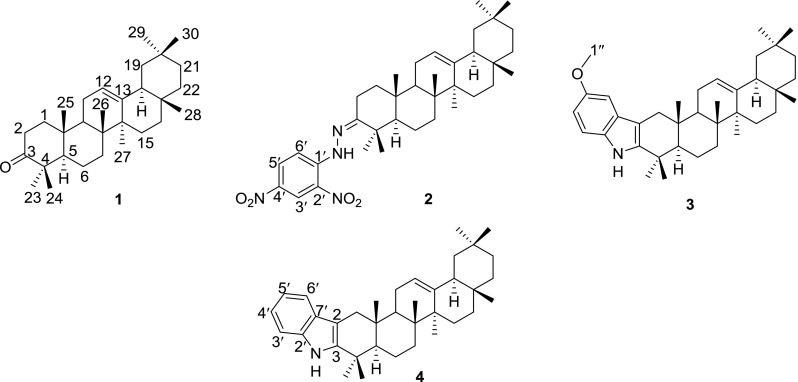
Structures of the compounds**1**-**4**

In thermal-assisted synthesis, compound **1** was combined with 2,4-dinitrophenylhydrazine, *p*-methoxyphenyl hydrazine or phenylhydrazine, were refluxed to yield compounds **2**–**4** as depicted in Scheme 1. The successful synthesis of compounds **2**–**4** was easily confirmed using comparative thin layer chromatography (TLC) profiles and their electronic impact mass spectra (EIMS) which showed an increasing mass as compared to that of **1** (*m/z* 424), implying an addition of chemical moieties to the starting raw compound. In fact, their high resolution EIMS (HREI-MS) revealed molecular ions peaks at *m/z* 604.4022 (calcd 604.3989 for C_36_H_52_N_4_O_4_), 527.4099 (calcd 527.4127 for C_37_H_53_NO), and 497.3993 (calcd 497.4022 for C_36_H_51_N) for compounds **2**, **3** and **4**, respectively. Their proton nuclear magnetic resonance (^1^H NMR) spectra exhibited broad singlet signals at *δ*_H_ 11.14, 7.56, 7.70 ppm due to NH in **2**, **3**, and **4**, respectively. In addition, they showed the same number or methyl group protons as in **1** except for compound **3** which has an additional methoxy methyl. Furthermore, some aromatic protons from Schiff base reagents were also revealed in these ^1^H NMR spectra. Their carbon-13 (^13^C NMR) spectra exhibited signals for 36 carbon atoms each. Apart from compound **2**, where the chemical shifts of carbon signals in the triterpene part were almost similar to those of **1** with comparatively moderate exceptions on C-2, C-3, and C-4, compounds **3** and **4** showed big chemical shift differences at the above mentioned positions. These big discrepancies observed in compounds **3** and **4** are due to the formation of the indole moiety which especially makes the carbons at position 2 (C-2) (*δ*_C_ 34.2, 20.7, 106.9, and 107.0 ppm for **1**, **2**, **3**, and **4**, respectively) to become aromatic and therefore, strongly deshielded as compared to the corresponding carbons in compounds **1** and **2**. The formation of the indole moiety in **3** and **4** was furthermore confirmed by the HMBC correlations observed between protons at position 1 [*δ*_H_ 2.17 (1H, d, *J* = 14.8 Hz, H-1a), 2.72 (1H, d, *J* = 14.8 Hz, H-1b) for **3** and 2.19 (1H, d, *J* = 15.0 Hz, H-1a), 2.77 (1H, d, *J* = 14.4 Hz, H-1b)] and carbons at 7′ [*δ*_C_ 128.5 (C-7′) for **3** and **4**]. The formation of the hydrazone moiety was also confirmed by HMBC correlations observed between the broad singlet at position *δ*_H_ 11.14 (NH) and C-3 (*δ*_C_ 167.1) (Fig. 1).

### Biological Activities

#### Urease Inhibition Activity

*β*-Amyrenone (**1**) and its synthetic derivatives (**2**-**4**) were screened for their UE inhibition activities. The IC_50_ values of the tested compounds are shown on Table 2. The results showed inhibition with IC_50_ values ranging from 14.5 to 24.6 μM (Table 2), with compound **1** being the most potent.

**Table 2:** Urease inhibition

Compound	Urease inhibition IC_50_ (μM)
**1**	14.5 ± 0.21
**2**	24.6 ± 0.17
**3**	22.2 ± 0.90
**4**	17.9 ± 0.45
Thiourea	21.6 ± 0.12

#### Docking of the Tested Compounds

**Fig. 2:**
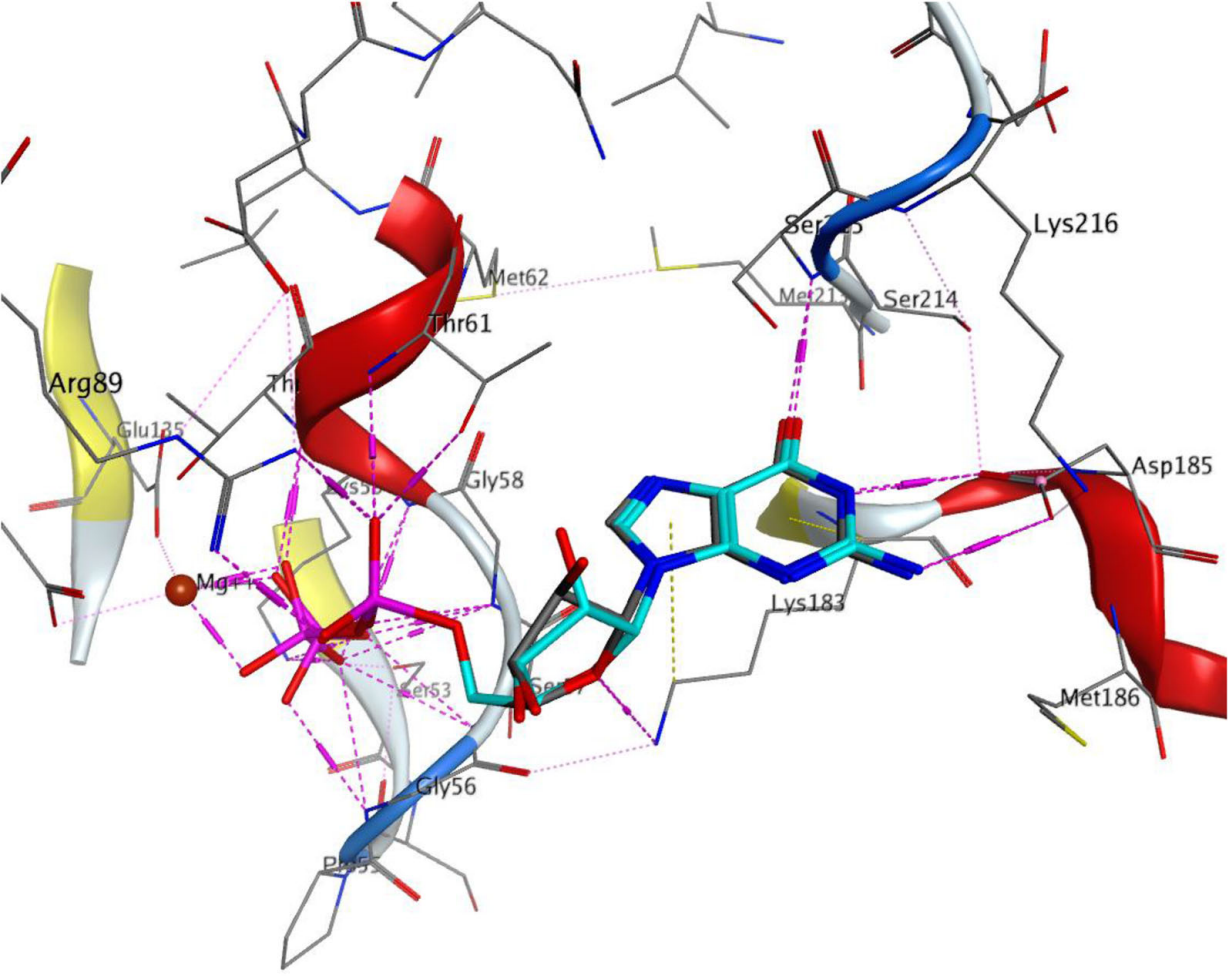
Re-docked pose of the co-crystallized small molecule. The docked pose in light blue while the X-ray structure is shown in cyan. H-bonds interactions are of the small molecules are shown as broken (dashed) magenta lines. Amino acid residues around the binding cavity are shown as sticks

**Fig. 3:**
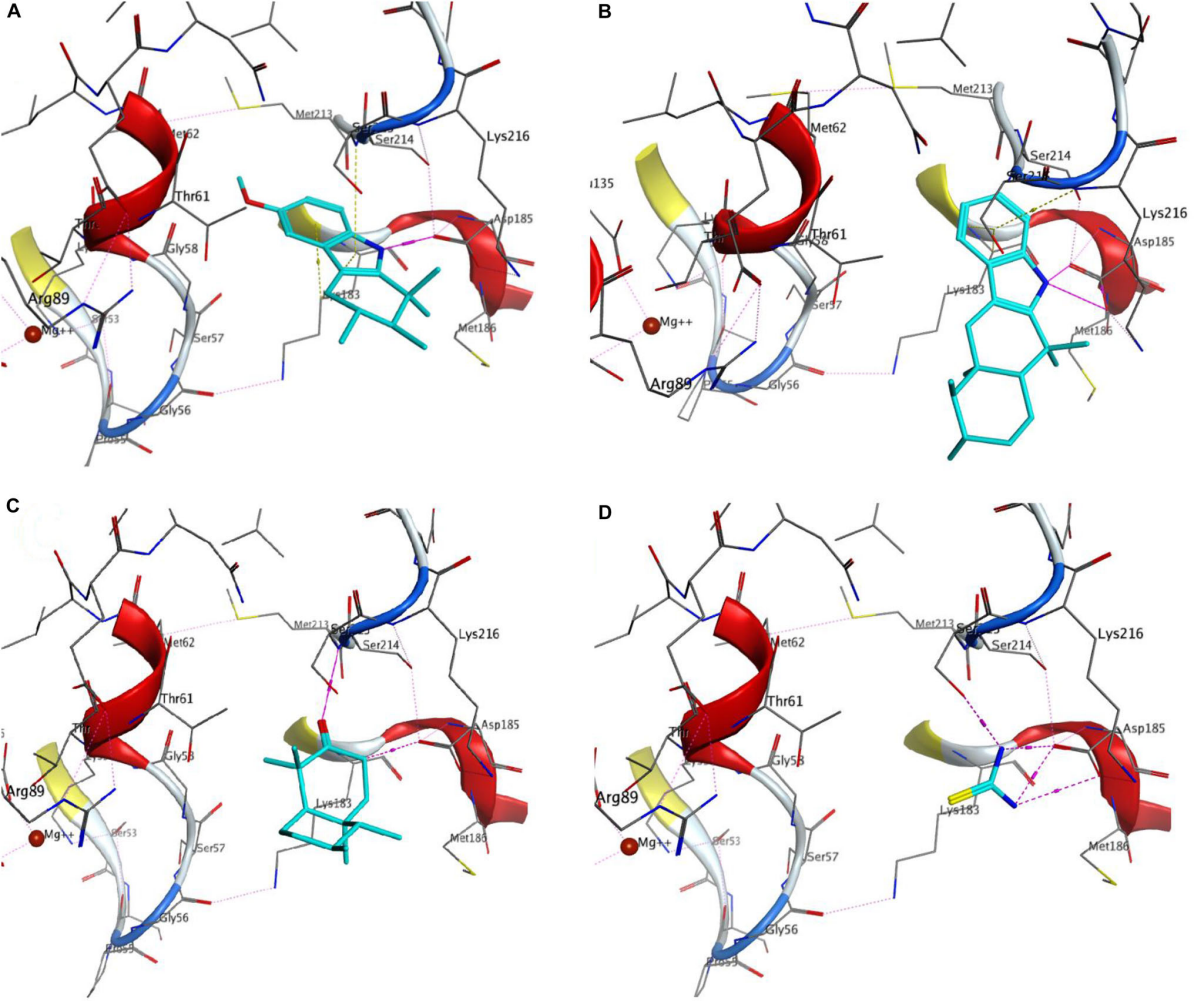
Docking poses of **a 3**, **b 2**, **c 1** and **d** Thiourea. Docked molecules are shown as light blue while the amino acid residues around the receptor pocket are shown as sticks. Hydrogen bonds and hydrophobic-π interactions are shown as dash magenta and yellow lines respectively

**Fig. 4:**
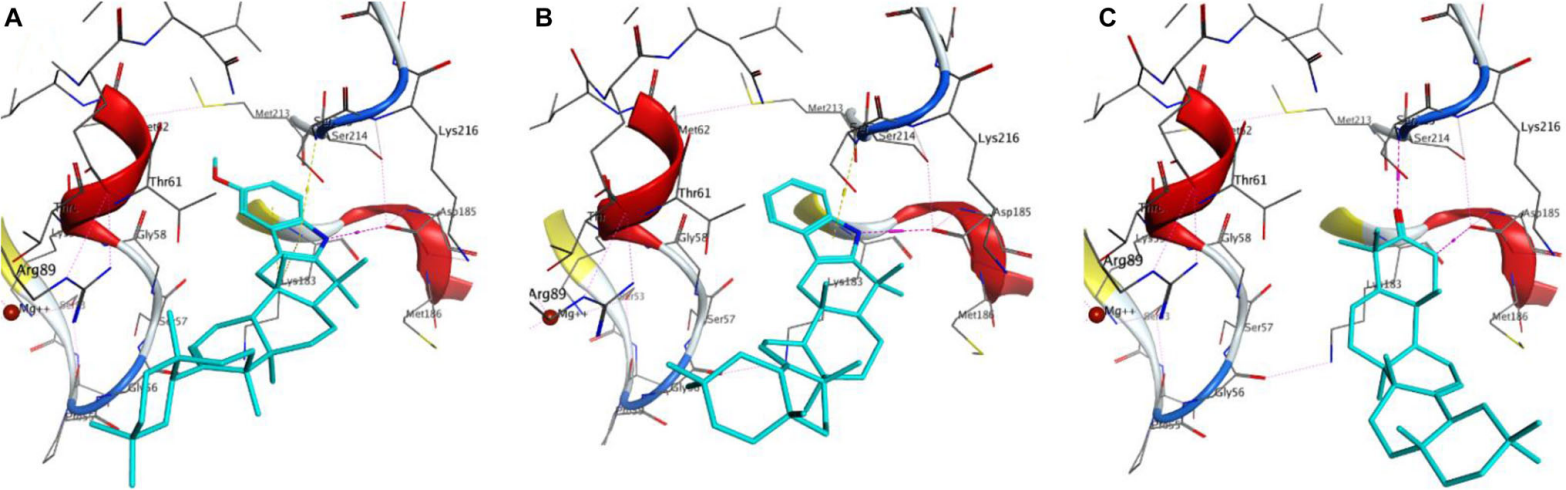
Displaying of the docked pose of the complete molecules **a 3**, **b 4**, **c 1**

The procedure used for docking validation of the native ligand (Fig. 2) was repeated on the same grid for docking *β*-amyrenone and its synthesised derivatives. The top scoring docking poses without the triterpene moiety (Fig. 3) and in the presence of the former (Fig. 4) have been shown. All compounds (**1**, **3** and **4**) and the moieties without the triterpene scaffold (**1′**, **3′** and **4′**, respectively), together with thiourea were able to dock the GDP binding site, except for compound **2** and its moiety after removing the triterpene scaffold, **2′**. The GlideScore (GS) standard precision (SP) docking scores and their various contributions; from rotatable bonds (GRB), lipophilicity (GS_lip_), H-bonding (GS_hb_), van der Waals interactions (GS_vdW_), electrostatic interactions (GS_ele_), along with the ligand efficiencies (LE) have been tabulated (Table 3). The observation that all docked compounds and moieties had weaker LE values, docking scores and contributions from H-bonding than the native ligand clearly points to the fact the binding of the native ligand (GDP) is rather driven by forces that are electrostatic in nature. On the contrary, the greatest contributions to binding of the compounds and their moieties are rather driven by lipophility and van der Waals interactions. The fact that docking of the Schiff base ligand and its moiety were both unable to give outputs (poses) after several attempts could imply that the inhibitions shown by this compound in the assays may be due to a mechanism that cannot be explained by docking to the cofactor binding site.

**Table 3:** Docking scores and contributions towards HP UE binding for the native ligand (GDP), the tested compounds (**1**, **3** and **4**), the compounds without the triterpene moiety (**1′**, **3′** and **4′**) and the reference thiourea

Compound	GRB^a^	LE^b^	GS (SP)^c^	GS_lip_^d^	GS_hb_^e^	GS_vdW_^f^	GS_ele_^g^
Native ligand	8	− 2.11	− 9.24	− 0.41	− 2.19	− 35.62	− 53.92
**1**	0	− 0.10	− 2.98	− 0.18	− 0.47	− 13.85	− 7.11
**3**	0	− 0.10	− 3.77	− 2.17	0.00	− 30.07	− 0.64
**4**	1	− 0.10	− 3.76	− 2.45	0.00	− 27.84	0.61
Thiourea	0	− 1.93	− 4.59	0	− 0.59	− 9.98	− 6.26
**1′**	0	− 1.04	− 3.93	− 1.15	0.00	− 19.08	0.62
**3′**	1	− 1.41	− 5.55	− 1.97	− 0.32	− 23.46	− 0.28
**4′**	0	− 1.16	− 4.85	− 2.16	0.00	− 24.00	− 0.64

^a^GlideScore contributions from rotatable bonds

^b^GlideScore ligand efficiency

^c^GlideScore standard precision

^d^GlideScore contributions from lipophilicity

^e^GlideScore contributions from H-bonding

^f^GlideScore contributions from van der Waals interactions

^g^GlideScore contributions from electrostatic interactions

##### Binding Interactions of the Tested Compounds Towards the Drug Target Site (2–4)

This was carried out on the cofactor binding site where GDP was co-crystallized with the protein. Since the tested compounds show highly hydrophobic scaffolds (with the highly hydrophobic triterpene moiety), contrary to the native GDP to the *H. pylori* UE target site was mostly driven by lipophilic and van der Waals interactions and less by H-bonding and electrostatic interactions, when compared with the native ligand (GDP), Table 3, the compounds were docked into the site without the characteristic triterpene moiety (Fig. 3) and in the presence of the latter (Fig. 4). It could be observed that, although the experimentally derived log values of the biological activities of all tested compounds were not so different, the parent compound (**1**) being tested to be the most active in the assays, the order of biological activities (**1 **> **4**> **3 **> **2**) could be reproduced in the GS SP docking scores for the tested compounds (**1 **> **4**> **3**) and their moieties without the triterpene scaffold (**1′ **> **4′ **> **3′**), no docking poses having been observed for both **2** and **2′** (Table 3). The two key protein–ligand interactions that could be observed in all docking poses for the tested compounds (Fig. 4), which were also observed in the binding with the co-crystallized GDP (Fig. 2) were those with the residues Gly56, Ser215 and Asp185. These same interactions were observed for the moieties without the triterpene scaffold and with thiourea (Fig. 3).

##### General Insights for Designing Tight Binding and More Potent Analogues

As could be expected, docking of very hydrophobic compounds unto a very polar binding site, would produce interactions which are different from those formed by the native GDP co-factor. Although a few native interactions were mimicked, due to the presence of the indole rings, it would make sense to synthesize even more analogues with other polar substituents susceptible to mimic H-bonding with the other binding site residues, e.g. Thr61, Lys183, Arg89 and Glu135. Since the introduction of the indole rings and creation of a Schiff base could only increase solubility without any improvement in biological activity, it might be much better to explore other regions of the *β*-amyrenone scaffold in order to obtain analogues with both improved activities and solubilities. Solubility predictions showed that the estimated solubilities of the tested compounds and the moieties without the triterpene scaffolds were about 2 log units less soluble than the native ligand (Supplementary Data). This implies the introduction of polar groups to the triterpene scaffold could both improve the solubility and binding by the improving the likelihood of formation of electrostatic interactions and H-bonds. Besides, docking of the moieties without the hydrophobic triterpene scaffold resulted in docking scores closer to those of thiourea and the native GDP. The former also showed less lipophilic and van der Waals contributions towards binding (Table 3). Moreover, none of the tested hits showed any PAINS alerts.

## Experimental Section

### Chemicals

The current study was conducted using the following reagents in technical, laboratory, and analytical grade solvents (Fisher): *n*-hexane, and ethyl acetate were used as pure or binary mixtures at different concentrations for purification of compounds. Column chromatography, used for first purification step, was performed on silica gel (230–400 mesh). Fractions were monitored by TLC using Merck pre-coated silica gel sheets (60 F_254_), and the detection of spots on the TLC plate was carried out using a vanillin spray (prepared by adding 15 g vanillin in 250 mL of ethanol followed by 2.5 mL of concentrated sulphuric acid) and heating the plate at about 80 °C. CDCl_3_ was used as solvent for ^1^H and ^13^C NMR experiments. Trifloroacetic acid, phenylhydrazine, 2,4-dinitrophenylhydrazine, and *p*-methoxyphenylhydrazine, all of analytical grade and purchased from Aldrich, USA, were used for synthesis. Olean-12-en-3-one commonly called *β*-amyrenone (**1**) was isolated from of the fruits of *Ficus vallis*-*choudae* Delile, collected from the North-West region of Cameroon [13]. The following reagents, all purchased from Sigma, were used for the urease assay: Phenol, sodium nitroprusside, dipotassium hydrogen phosphate trihydrate, EDTA, lithium chloride and thiourea.

### Apparatus

Melting points were measured on a Büchi M-560 melting point apparatus. Optical rotations were obtained with a JASCO DIP-360 polarimeter. UV spectra were recorded on a Hitachi UV 3200 spectrophotometer. A JASCO 320-A spectrophotometer was used for scanning IR spectroscopy using KBr pellets. 1D and 2D NMR spectra of compounds **1**-**4** recorded on a Bruker AM spectrometer operating at 150 and 600 MHz, respectively where chemical shifts (*δ*) were expressed in ppm with reference to the TMS. EI-MS spectra were obtained on Varian MAT 311A mass spectrometer operating at 300 °C. HPLC was carried out on recycling preparative High Performance Liquid Chromatogram of Japan Analytical Industry ltd. (Model LC-90 W). The column was of silica D-60 (internal diameter 20 mm, length 250 mm, particle size 40 µm. UV detector (Model 310) was used for detection.

### Compounds

#### *β*-Amyrenone (**1**)

Colourless polymorph solid (hexane–ethyl acetate 9.8:0.2); mp 166-167, IR (KBr) *ν*_max_ 1701 cm^−1^ (C=O); ^13^C-NMR (150 MHz, CDCl_3_): *δ* = 39.2 (C-1), 34.2 (C-2), 217.7 (C-3), 47.4 (C-4), 55.3 (C-5), 19.6 (C-6), 32.1 (C-7), 39.7 (C-8), 46.8 (C-9), 36.6 (C-10), 23.6 (C-11), 121.4 (C-12), 145.2 (C-13), 41.8 (C-14), 26.1 (C-15), 26.9 (C-16), 32.5 (C-17), 47.2 (C-18), 46.7 (C-19), 31.0 (C-20), 34.7 (C-21), 37.1 (C-22), 26.4 (C-23), 21.5 (C-24), 15.2 (C-25), 16.7 (C-26), 25.8 (C-27), 28.4 (C-28), 33.3 (C-29), 23.6 (C-30); EI-MS *m/z* (rel. int.%): 424 (M)^+^ (9.1), 409 (M-CH_3_)^+^ (7.0), 218 (100.0), 205 (16.0), 189 (15.7); Anal. Calcd. For C_30_H_48_O: C, 84.84; H, 11.39; O, 3.77. Found: C, 84.85; H, 11.38; O, 3.77.

#### (*E*)-1-(2,4-Dinitrophenyl)-2-(4,4,6a,6b,8a,11,11,14b-octamethyl-1,4,4a,5,6,6a,6b,7,8,8a,9,10,11,12,12a,14,14a,14b-octadecahydropicen-3(2H)-ylidene)hydrazine (**2**)

*β*-amyrenone (**1**) (40 mg, 0.094 mmol) was dissolved in dichloromethane (DCM) (1 mL). To this solution, a solution of 2,4-dinitrophenylhydrazine (18.67 mg, 0.094 mmol) in MeOH (1 mL) was added. To this mixture, 4 drops of TFA was also added. The reaction mixture was refluxed at 80 °C during 3 days. At completion of the reaction, the solvent was removed by evaporation under reduced pressure and the residue was suspended in cool water. After the suspension in water, the precipitate was successively chromatographed on silica gel column chromatography and HPLC eluting with the mixture of *n*-hexane-DCM (8:2) to afford **2** as an orange solid [*n*-hexane-DCM (8:2)], *R*_t_= 20 min, 46.69 mg, 81.94%; mp 260 °C; $[\alpha ]_{D}^{23}$ − 82.8 (*c* 0.0001, CHCl_3_); UV (CHCl_3_) λ_max_(logε) 364 (3.18) nm; IR (KBr) *ν*_max_ 3450 (NH), 2922 (C–H); 1620 (C=C), 1592 (N–O), 1509 (C=N) cm^−1^; ^1^H-NMR: (CDCl_3,_ 300 MHz): *δ* = 2.50–2.39 (1H, m, H-2a), 2.64–2.57 (1H, m, H-2b), 5.19 (1H, t, *J* = 3.0 Hz, H-12), 0.83 (3H, s, H-23), 1.17 (3H, s, H-24), 1.04 (3H, s, H-25), 1.12 (3H, s, H-26), 1.02 (3H, s, H-27), 1.3.0 (3H, s, H-28), 0.85 (6H, s, H-29, H-30), 9.10 (1H, d, *J* = 2.7 Hz, H-3′), 8.27 (1H, dd, *J* = 9.6, 2.4 Hz, H-5′), 7.94 (1H, d, *J* = 9.6 Hz, H-6′), 11.14 (1H, s, NH);^13^C-NMR: (CDCl_3_, 150 MHz): *δ* = 38.1 (C-1), 20.7 (C-2), 167.1 (C-3), 42.2 (C-4), 55.4 (C-5), 19.7 (C-6), 32.3 (C-7), 39.8 (C-8), 46.7 (C-9), 36.7 (C-10),23.6 (C-11), 121.4 (C-12), 145.3 (C-13), 41.8 (C-14), 26.1 (C-15), 26.9 (C-16), 32.5 (C-17), 47.3 (C-18), 46.7 (C-19), 31.1 (C-20), 34.7 (C-21), 37.1 (C-22), 28.4 (C-23), 21.5 (C-24), 14.9 (C-25), 25.9 (C-26), 16.8 (C-27), 28.8 (C-28), 33.3 (C-29), 23.7 (C-30), 145.6 (C-1′), 128.9 (C-2′), 123.6 (C-3′), 137.5 (C-4′), 129.9 (C-5′), 116.5 (C-6′); EI-MS *m/z* (rel. int.%): 604 [M]^+^ (20), 589 (3), 386 (6), 305 (11), 218 (100), 203 (75), 109 (34) and 55 (47); HREIMS *m/z* 604.4022 (Calcd 604.3989 for C_36_H_52_N_4_O_4_); Anal. Calcd. For C_36_H_52_N_4_O_4_: C, 71.49; H, 8.67; N, 9.26, O, 10.58. Found: C, 71.48; H, 8.67; N, 9.27, O, 10.58.

#### (4a*R*,6a*S*,6b*R*,8a*R*,15a*R*,17b*S*)-13-Methoxy-2,2,4a,6a,6b,9,9,15a-octamethyl-2,3,4,4a,5,6,6a,6b,7,8,8a,9,10,15,15a,15b,16,17b-octadecahydro-1*H*-chryseno[2,1-b]carbazole (**3**)

The synthesis of compound (**3**) was similar to that of (**2**) but with different Schiff base, temperature and reaction time. In this case, 2,4-dinitrophenylhydrazine was replaced by *p*-methoxyphenylhydrazine (13.01 mg, 0.094 mmol) and the reaction mixture was rather refluxed at 110 °C for 6 days. At completion of the reaction, the final mixture was treated as in the previous case and also successively chromatographed on silica gel column chromatography and HPLC eluting with the mixture of *n*-hexane-DCM (9.8:0.2) to yield **3** as an off white solid [*n*-hexane-DCM (8:2)], *R*_t_= 50 min, 40 mg, 80.46%, mp 255; $[\alpha ]_{D}^{23}$ − 96 (*c* 0.001, CHCl_3_); UV (CHCl_3_) λ_max_(logε) 283 (3.89) nm; IR(KBr) ν_max_: 3420 (N–H), 2922 (C-H), 1459 (C=C), 1211 (C–N) and 1086 (C–O) cm^−1^; ^1^H-NMR: (CDCl_3_, 400 MHz): *δ* = 2.17 (1H, d, *J* = 14.8 Hz, H-1a), 2.72 (1H, d, *J* = 14.8 Hz, H-1b), 5.28 (1H, t, *J* = 3.6 Hz, H-12), 3.82 (s, H-1″), 1.20 (3H, s, H-27) 1.17 (3H, s, H-23), 1.05 (3H, s, H-24), 0.96 (3H, s, H-25), 0.88 (3H, s, H-28), 0.87 (3H each, s, H-29 and H-30), 0.85 (3H, s, H-26), 7.56 (1H, s, NH-1′), 6.75 (1H, dd, *J* = 8.4, 2.4 Hz, H-3′), 7.16 (1H, d, *J* = 8.4 Hz, H-4′), 6.87 (1H, d, *J* = 2.4 Hz, H-6′); ^13^C-NMR: (CDCl_3_, 100 MHz): *δ* = 36.9 (C-1), 106.9 (C-2), 141.9 (C-3), 32.5 (C-4), 53.1 (C-5), 19.3 (C-6), 32.1 (C-7), 39.9 (C-8), 47.3 (C-9), 38.0 (C-10), 23.6 (C-11), 121.9 (C-12), 145.0 (C-13), 41.9 (C-14), 26.1 (C-15), 27.0 (C-16), 34.0 (C-17), 46.3 (C-18), 46.8 (C-19), 31.1 (C-20), 34.7 (C-21), 37.1 (C-22), 31.0 (C-23), 23.7 (C-24), 15.8 (C-25), 25.8 (C-26), 16.6 (C-27), 28.5 (C-28), 33.4 (C-29), 23.4 (C-30), 131.1 (C-2′), 110.6 (C-3′), 110.9 (C-4′), 153.7 (C-5′), 100.3 (C-6′), 128.5 (C-7′), 56.0 (C-1″); EI-MS *m/z* (rel. int.%): 527 [M] ^+^ (100), 240 (9), 201 (51), 83 (40); HREIMS *m/z* 527.4099 (Calcd 527.4127 for C_37_H_53_NO); Anal. Calcd. For C_37_H_53_NO: C, 84.19; H, 10.13; N, 2.65, O, 3.03. Found: C, 84.18; H, 10.13; N, 2.65, O, 3.03.

#### (4a*R*,6a*S*,6b*R*,8a*R*,15a*R*,17b*S*)-2,2,4a,6a,6b,9,9,15a-Octamethyl-2,3,4,4a,5,6,6a,6b,7,8,8a,9,10,15,15a,15b,16,17b-octadecahydro-1*H*-chryseno[2,1-b]carbazole (**4**)

The synthesis of compound (**4**) was also similar to that of compounds (**2**) and (**3**) with phenylhydrazine (10.19 mg, 0.094 mmol) as in the two previous reactions. Here, the reaction mixture was refluxed at 230 °C within 8 days. At completion of the reaction, the final mixture was treated as in the previous cases and equally successively chromatographed on silica gel column chromatography and HPLC eluting with the mixture of *n*-hexane-DCM (8:2) to yield **3** as an off white solid [*n*-hexane-DCM (8:2)], *R*_t_= 20 min, 45.85 mg, 97.79%, mp 254.6; $[\alpha ]_{D}^{23}$ − 47.5 (*c* 0.001, CHCl_3_); UV (CHCl_3_) λ_max_(logε) 283 (3.49) nm; IR (KBr) ν_max_: 3420 (N–H), 2916 (C-H), 1461 (C=C) and 1300 (C–N) cm^−1^; ^1^H-NMR: (CDCl_3_, 600 MHz): *δ* = 2.19 (1H, d, *J* = 15.0 Hz, H-1a), 2.77 (1H, d, *J* = 14.4 Hz, H-1b), 5.28 (1H, brt, H-12) 1.30 (3H, s, H-23), 1.21 (3H, s, H-24), 0.96 (3H, s, H-25), 1.05 (3H, s, H-26), 1.17 (3H, s, H-27), 0.85 (3H, s, H-28), 0.87 (3H, s, H-29), 0.88 (3H each, s, H-30), 7.70 (1H, s, NH-1′), 7.28 (1H, d, *J* = 7.8 Hz, H-3′), 7.09 (1H, t, *J* = 7.2 Hz, H-4′), 7.04 (1H, t, *J* = 7.2 Hz, H-5′), 7.40 (1H, d, *J* = 7.8 Hz, H-6′); ^13^C-NMR: (CDCl_3_, 150 MHz): *δ* = 36.9 (C-1), 106.9 (C-2), 141.9 (C-3), 32.5 (C-4), 53.1 (C-5), 19.3 (C-6), 32.1 (C-7), 39.9 (C-8), 47.3 (C-9), 38.0 (C-10), 23.6 (C-11), 121.9 (C-12), 145.0 (C-13), 41.9 (C-14), 26.1 (C-15), 27.0 (C-16), 34.0 (C-17), 46.3 (C-18), 46.8 (C-19), 31.1 (C-20), 34.7 (C-21), 37.1 (C-22), 31.0 (C-23), 23.7 (C-24), 15.8 (C-25), 25.8 (C-26), 16.6 (C-27), 28.5 (C-28), 33.4 (C-29), 23.4 (C-30), 131.1 (C-2′), 110.6 (C-3′), 110.9 (C-4′), 153.7 (C-5′), 100.3 (C-6′), 128.5 (C-7′); EI-MS *m/z* (rel. int.%): 497 [M]^+^ (100), 279 (14), 210 (14), 171 (44); HREIMS: *m/z* 497.3993 (Calcd 497.4022 for C_36_H_51_N); Anal. Calcd. For C_36_H_51_N: C, 86.86; H, 10.33; N, 2.81. Found: C, 86.85; H, 10.33; N, 2.82.

### Biological Activities

#### Urease Inhibition Assay

Urease activity was obtained by measuring ammonia production using the indophenol method as described by Bankeu et al., (2017). Thiourea was used as the standard inhibitor of urease for this assay. Reaction mixtures with 25 μL of enzyme (Jack bean Urease bought from Sigma) solution and 55 μL of buffers containing 100 mM urea were incubated with 5 μL of compounds **1**—**4** (1 mM concentration) at 30 °C for 15 min in 96-well plates. In fact, 45 μL each of phenol reagent (1% w/v phenol and 0.005% w/v sodium nitroprusside) and 70 μL of alkali reagent (0.5% w/v NaOH and 0.1% active chloride NaOCl) were added to each well. The absorbance at 630 nm was recorded after 50 min, using a microplate reader (Molecular Device, USA). All reactions were performed in triplicate in a final volume of 200 μL. The results (change in absorbance per min) were processed by using Soft Max Pro software (Molecular Device, USA). All the assays were performed at pH 8.2 (0.01 M K_2_HPO_4_**·**3H_2_O, 1 mM EDTA and 0.01 M LiCl). Percentage inhibitions were calculated from the formula (Eq. 1):

1 $${\text{Urease inhibition }}\left( \% \right) \, = { 1}00\,{-}\,\left( {{\text{OD}}_{\text{test}} /{\text{OD}}_{\text{control}} } \right)\, \times \, 100$$

where OD stands for optical density.

Urease inhibition percentages were used to determine the concentration that inhibit 50% of urease activity (IC_50_) using GraphPad Prism 5.0, USA.

#### Statistical Analysis

The resulting data are shown as mean ± SD of three independent assays. One way one analysis of variance (ANOVA) was carried out for the determination of difference between groups (GraphPad Prism 5.0, USA). P > 0.05 was considered as significant.

#### Molecular Docking Studies

Several structures for *H. pylori* urease have been deposited in the protein databank, e.g. PDB codes; 3O1Q (1.85 Å resolution, [27]), 3SF5 (2.49 Å resolution, [27]), 4HI0 (2.35 Å resolution, [28]) and 4LPS (2.00 Å resolution, [6]). Most of the deposited crystal structures had no ligand or co-factor in the receptor active site. 4LPS was chosen because, in addition to the presence of the co-crystallized a co-factor within the receptor site, this crystal structure has a good resolution. In an attempt to explain the observed inhibitory activity of the *β*-amyrenone and derivatives against *H. pylori*, the compounds were docked into the receptor active site of the 4LPS crystal structure [6]. This enzyme is known to possess complex metallocenters that are assembled by teams of proteins in multistep pathways. The aim was to observe the target site interactions which could explain why the parent compound was more active than its synthesized analogues and to explore regions of the biding site which could be further exploited for analogue design.

##### Docking Validation of Native Ligands in HP UE Binding Site

Validation of the docking protocol was done by the re-docking of the co-crystallized ligand (GDP) into the catalytic pocket of 4LPS. The binding pose of the co-crystallized ligands could be reproduced by the docking program with root mean square deviation (rmsd) values < 0.4 Å. The docking scores and various contributions towards binding have been shown on Table 3 for the co-crystallized ligand, along with tested compounds (**1**—**4**). Generally, the docked-pose of the native ligand in the active site of 4LPS showed similar interactions to the observed binding mode of the resolved *H. pylori* crystal structure (PDB: 4LPS) and the adenine (nitrogenous base) group was well accommodated into receptor active site. Key H-bond interaction between the adenine group and amino acid residue in the binding site could be reproduced, including those with Ser215, Thr61, Asp185, Gly56, Lys183, Arg89 and Glu135 via the Mg^2+^ cation (Fig. 2).

#### Molecular Modelling

The ligands and protein–ligand complex used for the in silico studies were prepared as follows:

##### Protein Preparation and Binding Site Analysis

The high resolution crystal structure [6] of HypB from *H. pylori* (PDB ID: 4LPS) was downloaded from the Protein Databank (PDB; www.rcsb.org) [14]. All water molecules were deleted using the MOE software [15]. Protein Preparation Wizard of Schrödinger software was subsequently employed for further preparations of the protein structure using the default settings [16, 17]. Bond orders were assigned and hydrogen atoms added as well as protonation of the heteroatom states using Epik-tool (with the pH set at biologically relevant values, i.e. at 7.0 ± 2.0). The H-bond network was then optimized. The structure was finally subjected to a restrained energy minimization step (rmsd of the atom displacement for terminating the minimization was 0.3 Å), using the Optimized Potentials for Liquid Simulations (OPLS) 2005 force field [18].

##### Ligand Preparation

The default setting of the LigPrep tool implemented in Schrödinger’s software (version 2017-1) was used to prepare the ligands for docking [19]. All possible tautomers and combination of stereoisomers were generated for pH 7.0 ± 2.0, using the Epik ionization method [17]. Energy minimization was subsequently done using the integrated OPLS 2005 force field [18]. Pan-Assay Interference (PAIN) filters were applied using Schrodinger’s Canvas tool [20] and the CbLigand web server [21]. Furthermore, ConfGen was used to generate 50 conformers of each prepared ligand, using the default settings and allowing minimization of the output conformations [22, 23].

##### Docking and Pose Validation

Docking was done using the Glide program [24–26]. The receptor grid preparation for the docking procedure was carried out by assigning the co-crystallized nucleotide as the centroid of the grid box. The generated 3D conformers were docked into the receptor model using Glide using the Standard Precision (SP) mode as the scoring function [24, 25]. A total of 5 poses per ligand conformer were included in the post-docking minimization step, and a maximum of 2 docking poses was generated for each ligand conformer. The proposed docking procedure was able to re-dock the crystallized nucleotide within the receptor binding pocket with rmsd < 0.4 Å. The re-docked nucleotide pose was observed to reproduce the key H-bonds interaction with the target site.

## Conclusion

The synthesis and testing of novel analogues of a naturally occurring compound *β*-amyrenone has been carried out in order to derive three new compounds (two indoles and one Schiff base), having activities in the lower micromolar range in the *H. pylori* UE assay. The nitrogenous derivatives showed improved solubilities with respect to the parent compounds, but without a consequent improvement in biological activities. Docking the tested compounds and their less hydrophobic moieties towards the cofactor binding site of *H. pylori* UE showed that the binding of the tested compounds were more driven by hydrophobic and van der Waals interactions contrary to the native ligand. The exploitation of other regions of the parent compound scaffold would therefore be the most logical approach to improve activities. It is expected that the obtained results would serve as ground work for efforts towards the discovery of novel antimicrobials targeting HP infection from natural sources.

## Electronic supplementary material

Below is the link to the electronic supplementary material.

Supplementary material 1 (PDF 6166 kb)
