# Supplementary material for: Synthesis, Urease Inhibition and Molecular Modelling Studies of Novel Derivatives of the Naturally Occurring β-Amyrenone
Source: Nat Prod Bioprospect. 2018 Nov 28;9(1):49–59. doi: 10.1007/s13659-018-0193-7 (PMC6328428; doi:10.1007/s13659-018-0193-7)
Supplement: Supplementary file 1 — Supplementary material 1 (PDF 6166 kb) [file 13659_2018_193_MOESM1_ESM.pdf]

Electronic Supplementary Material

Synthesis, urease inhibition and molecular modelling studies of novel derivatives of the naturally occurring *β*-amyrenone

Jean J.K. Bankeu[a,b,*] · Hira Sattar[b] · Yannick S.F. Fongang[b,c] · Syeda W. Muhammadi[b] · Conrad V. Simoben[d] · Fidele Ntie-Kang[d,e,*] · Guy R.T. Feuya[f] · Marthe A.T. Tchuenmogne[g] · Mehreen Lateef[h] · Bruno N. Lenta[i,*] · Muhammad S. Ali[b] · Augustin S. Ngouela[g]

✉ Jean J.K. Bankeu

bk_jeanjules@yahoo.fr

✉ Fidele Ntie-Kang

ntiekfidele@gmail.com or fidele.ntie-kang@ubuea.cm

✉ Bruno N. Lenta

lentabruno@yahoo.fr

[1] Department of Chemistry, Faculty of Science, The University of Bamenda, P.O. Box 39, Bambili, Cameroon

[2] International Center for Chemical and Biological Sciences, University of Karachi, Karachi 75270, Pakistan

[3] Department of Chemistry, Higher Teacher Training College, University of Maroua, P.O. Box 55, Maroua, Cameroon

[4] Department of Pharmaceutical Chemistry, Martin-Luther University of Halle-Wittenberg, Wolfgang-Langenbeck-Str. 4, 06120, Halle, Saale, Germany

[5] Department of Chemistry, Faculty of Science, University of Buea, P. O. Box 63, Buea, Cameroon

[6] Department of Chemistry, Faculty of Science, Scientific and Technical University of Masuku, Box 943, Franceville, Gabon

[7] Department of Chemistry, Faculty of Science, University of Yaoundé I, P.O. Box 812, Yaoundé, Cameroon

[8] Multi-Disciplinary Research Laboratory (MDRL), Bahria University Medical and Dental College, Karachi, Pakistan

[9] Department of Chemistry, Higher Teacher Training College, University of Yaoundé I, P.O. Box 47 Yaoundé, Cameroon.

Table of Contents

S1: ^{1}H-NMR spectrum of compound **1** 4

S2: ^{13}C-NMR spectrum of compound **1** 5

S3: ^{1}H-NMR spectrum of compound **2** 8

S4: ^{13}C-NMR spectrum of compound **2** 9

S5: ^{13}C DEPT-135 spectrum of compound **2** 10

S6: ^{13}C DEPT-90 spectrum of compound **2** 11

S7: DEPT-HSQC spectrum of compound **2** 12

S8: HMBC spectrum of compound **2** 13

S9: COSY spectrum of compound **2** 14

S10: NOESY spectrum of compound **2** 15

S11: EIMS spectrum of compound **2** 16

S12: HR-EIMS spectrum of compound **2** 17

S13: ^{1}H-NMR spectrum of compound **3** 24

S14: ^{13}C-NMR spectrum of compound **3** 25

S15: ^{13}C DEPT-135 spectrum of compound **3** 26

S16: ^{13}C DEPT-90 spectrum of compound **3** 27

S17: DEPT-HSQC spectrum of compound **3** 29

S18: HMBC spectrum of compound **3** 32

S19: COSY spectrum of compound **3** 34

S20: NOESY spectrum of compound **3** 35

S21: EIMS spectrum of compound **3** 36

S22: HR-EIMS spectrum of compound **3** 37

S23: ^{1}H-NMR spectrum of compound **4** 43

S24: ^{13}C-NMR spectrum of compound **4** 44

S25: ^{13}C DEPT-135 spectrum of compound **4** 45

S26: ^{13}C DEPT-90 spectrum of compound **4** 46

S27: DEPT-HSQC spectrum of compound **4** 47

S28: HMBC spectrum of compound **4** 48

S29: COSY spectrum of compound **4** 49

S30: NOESY spectrum of compound **4**... 50

S31: EIMS spectrum of compound **4**.. 51

S32: HR-EIMS spectrum of compound **4**.. 52

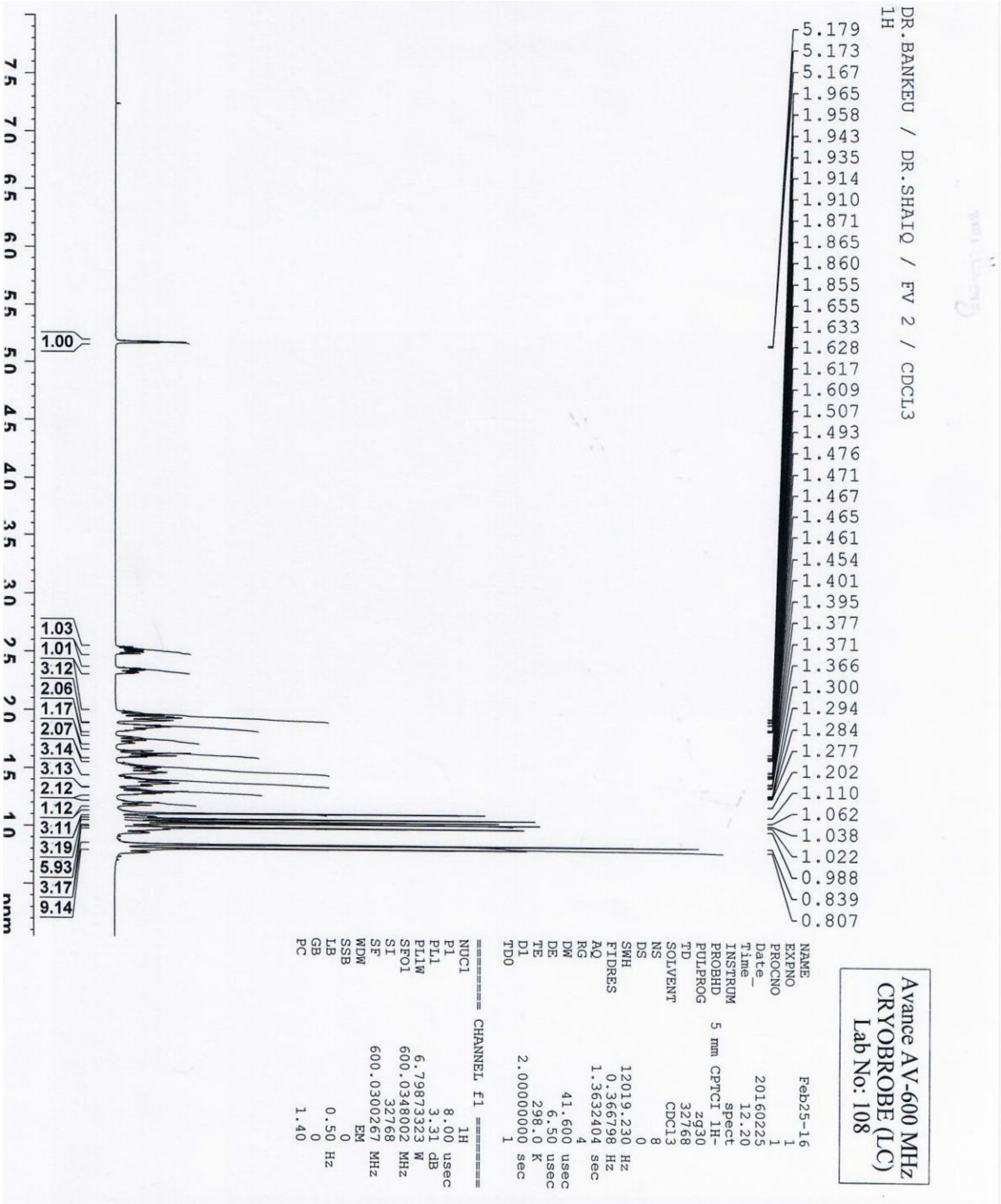

S1: ^{1}H-NMR spectrum of compound **1**

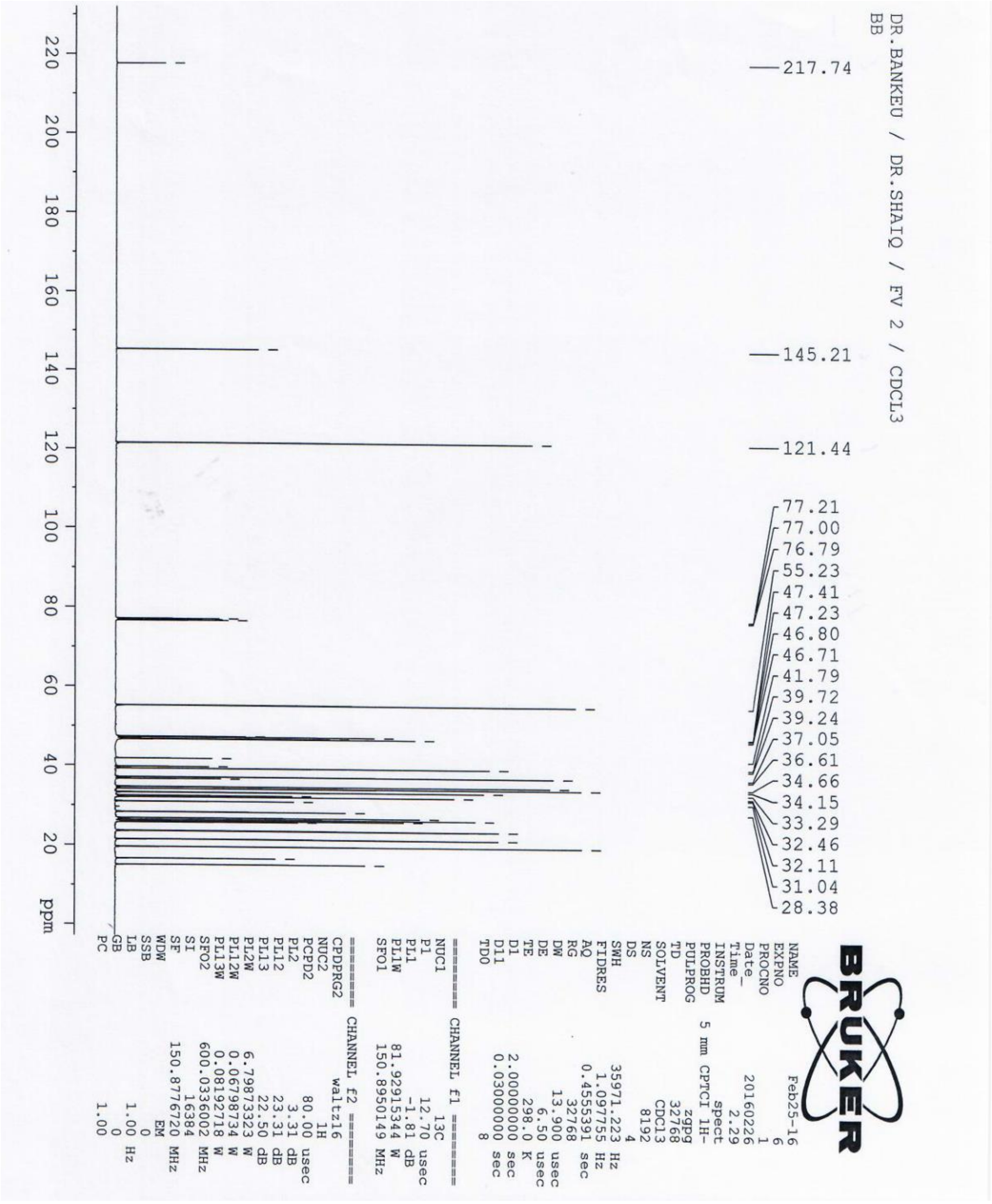

S2: ^{13}C-NMR spectrum of compound **1**

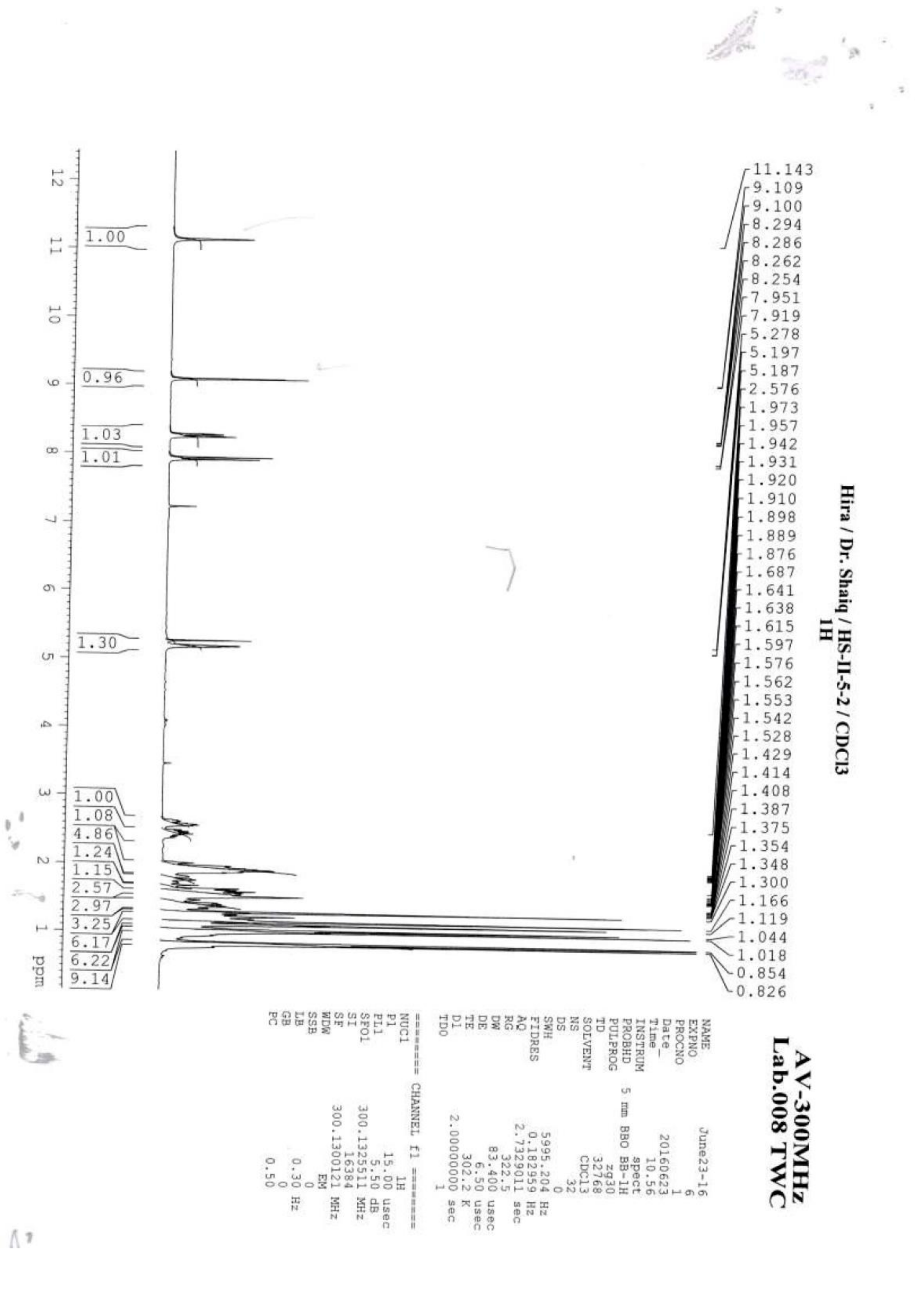
Hira / Dr. Shaiq / HS-II-5-2 / CDCl3
1H
AV-300MHz
Lab.008 TWC
11.143
9.109
9.100
8.294
8.286
8.262
8.254
7.951
7.919
5.278
5.197
5.187
2.576
1.973
1.957
1.942
1.931
1.920
1.910
1.898
1.889
1.876
1.687
1.641
1.638
1.615
1.597
1.576
1.562
1.553
1.542
1.528
1.429
1.414
1.408
1.387
1.375
1.354
1.348
1.300
1.166
1.119
1.044
1.018
0.854
0.826
NAME June23-16
EXPNO 6
PROCNO 1
Date_ 20160623
Time 10.56
INSTRUM spect
PROBHD 5 mm BBO BB-1H
PULPROG zg30
TD 32768
SOLVENT CDCl3
NS 32
DS 0
SWH 5995.204 Hz
FIDRES 0.182959 Hz
AQ 2.7329011 sec
RG 322.5
DW 83.400 usec
DE 6.50 usec
TE 302.2 K
D1 2.00000000 sec
TD0 1
======== CHANNEL f1 ========
NUC1 1H
P1 15.00 usec
PL1 5.50 dB
SFO1 300.1325511 MHz
SI 16384
SF 300.1300121 MHz
WDW EM
SSB 0
LB 0.30 Hz
GB 0
PC 0.50
1.00
0.96
1.03
1.01
1.30
1.00
1.08
4.86
1.24
1.15
2.57
2.97
3.25
6.17
6.22
9.14
12
11
10
9
8
7
6
5
4
3
2
1
ppm

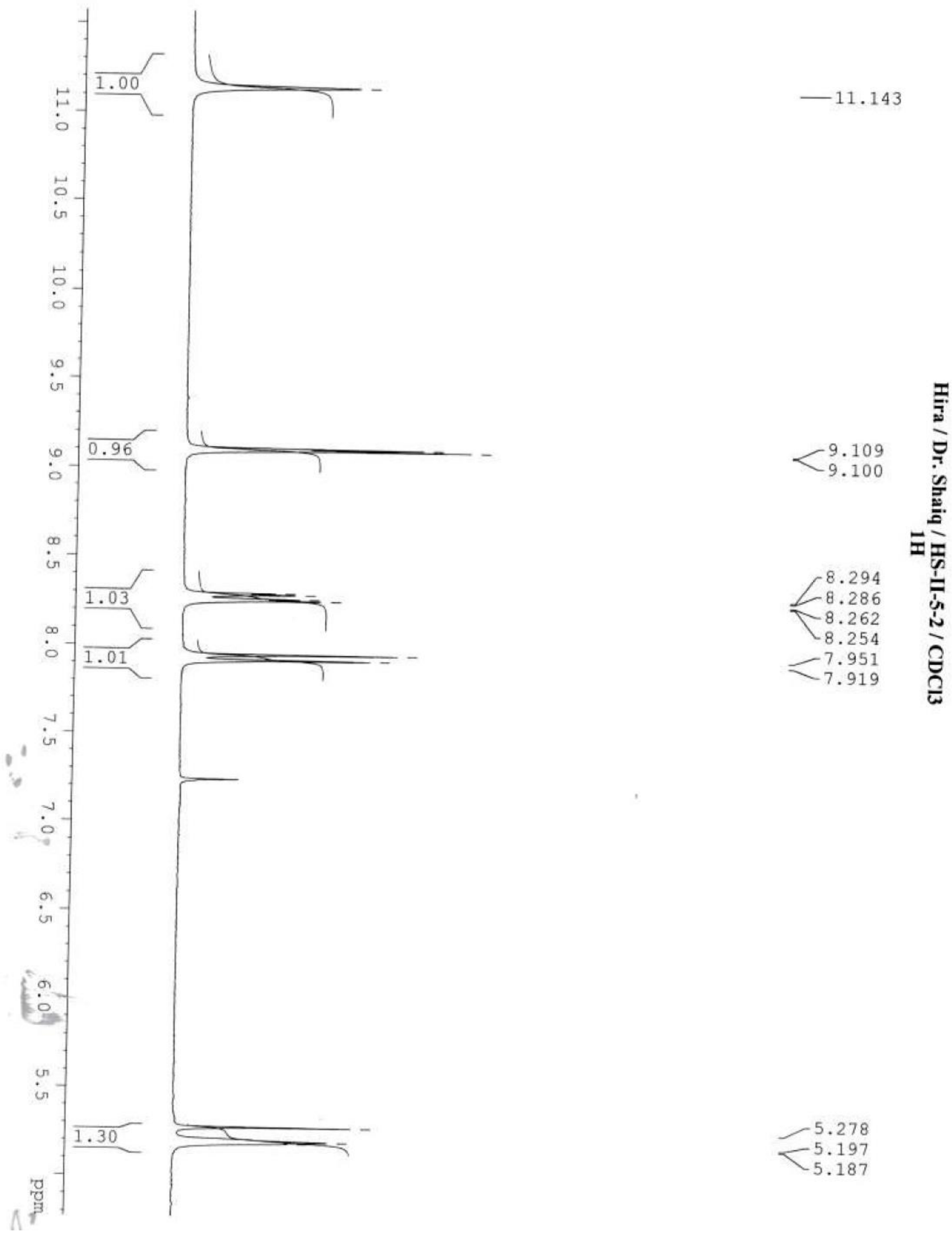

Hira / Dr. Shaiq / HS-II-5-2 / CDCl3
1H
11.143
9.109
9.100
8.294
8.286
8.262
8.254
7.951
7.919
5.278
5.197
5.187
1.00
0.96
1.03
1.01
1.30
11.0
10.5
10.0
9.5
9.0
8.5
8.0
7.5
7.0
6.5
6.0
5.5
ppm

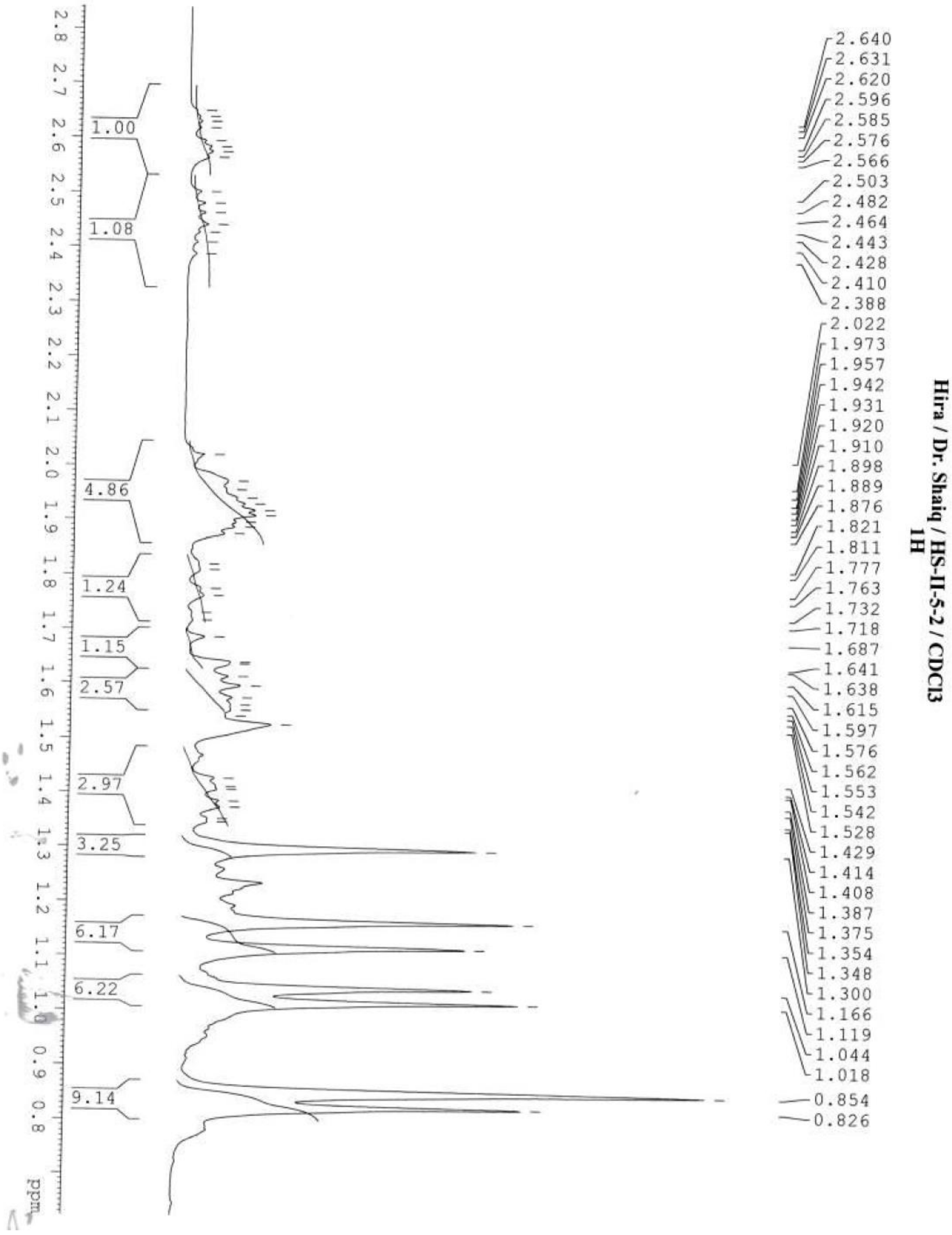

S3: ^{1}H-NMR spectrum of compound **2**

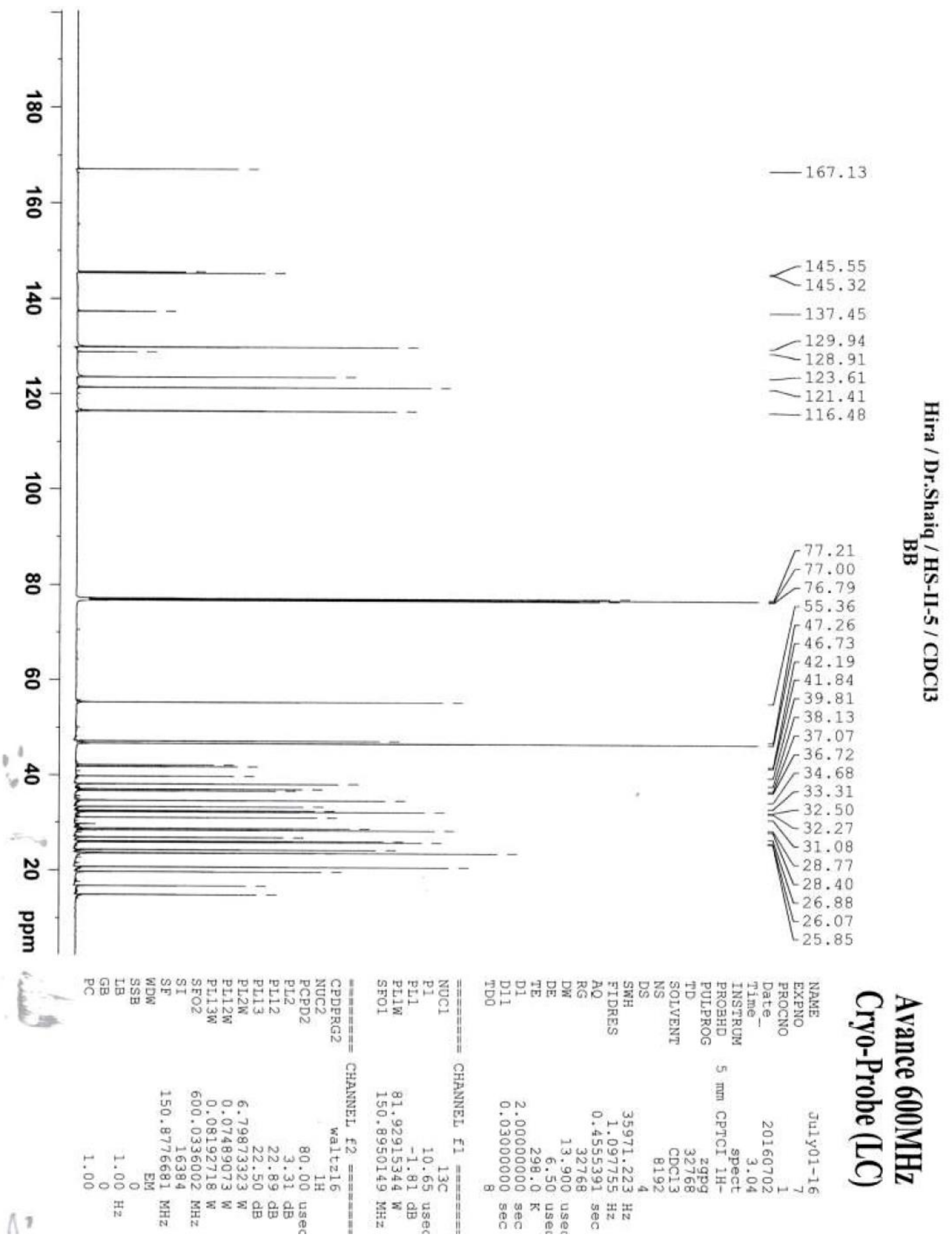

S4: ^{13}C-NMR spectrum of compound **2**

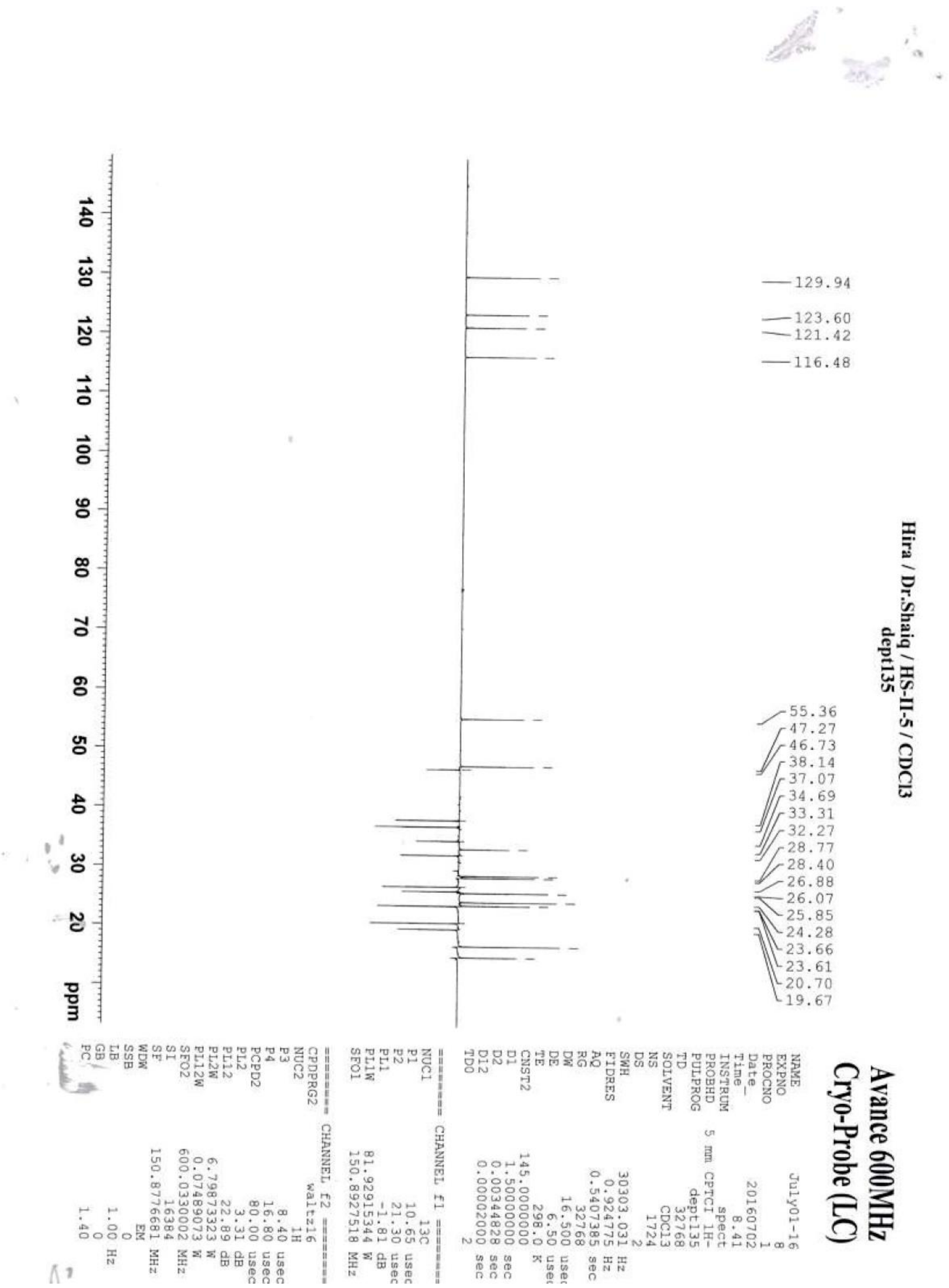

S5: ^{13}C DEPT-135 spectrum of compound **2**

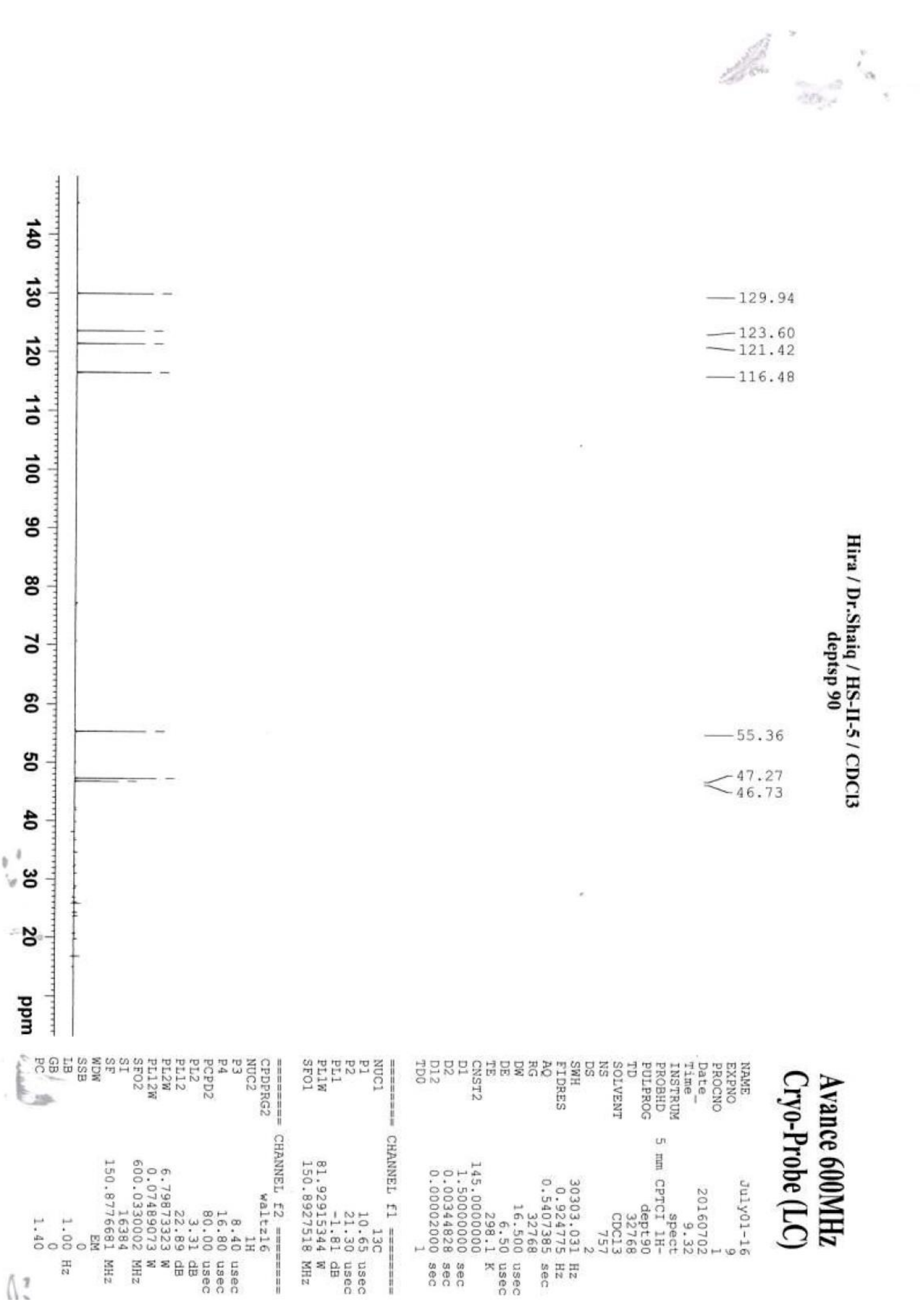

S6: ^{13}C DEPT-90 spectrum of compound **2**

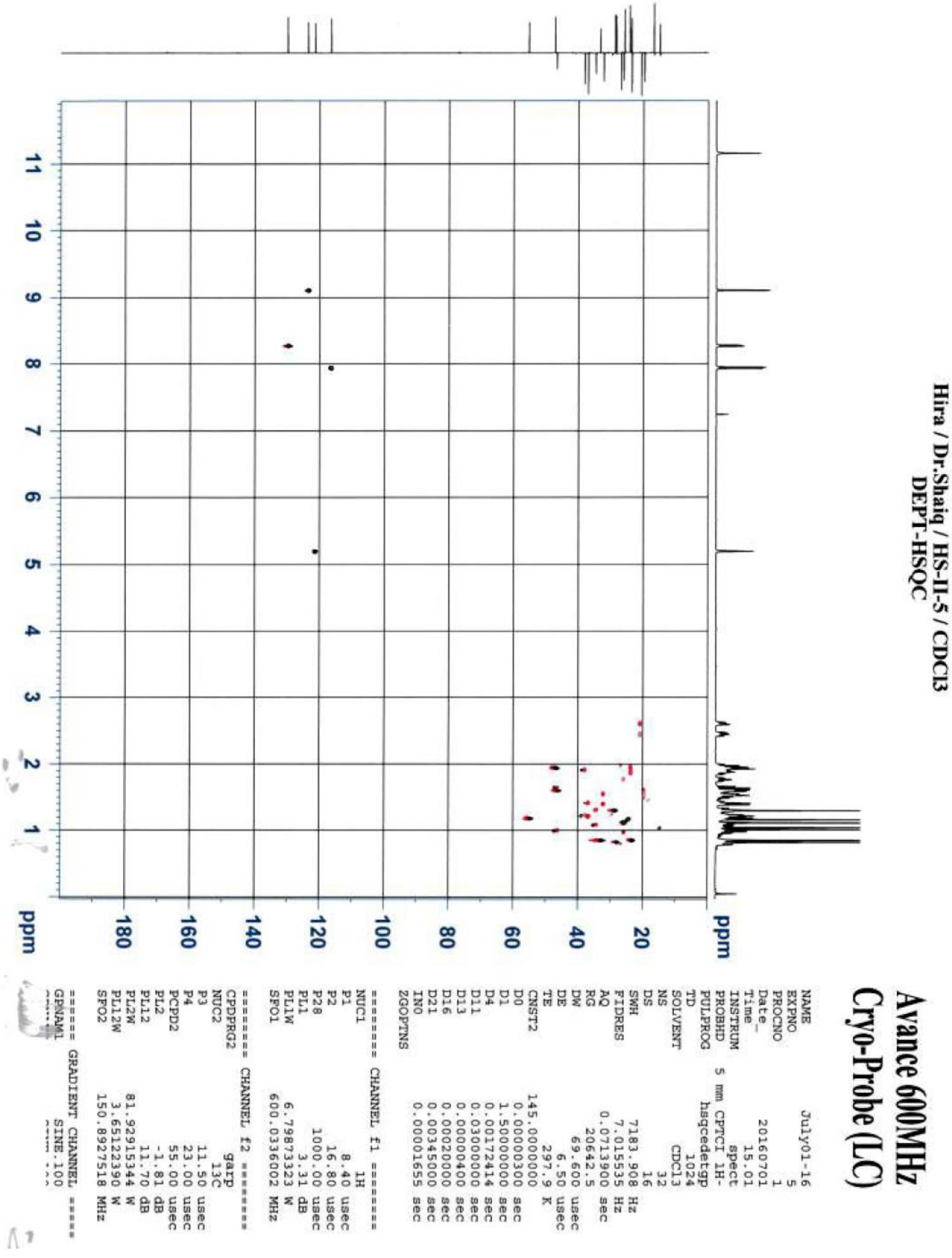

S7: DEPT-HSQC spectrum of compound **2**

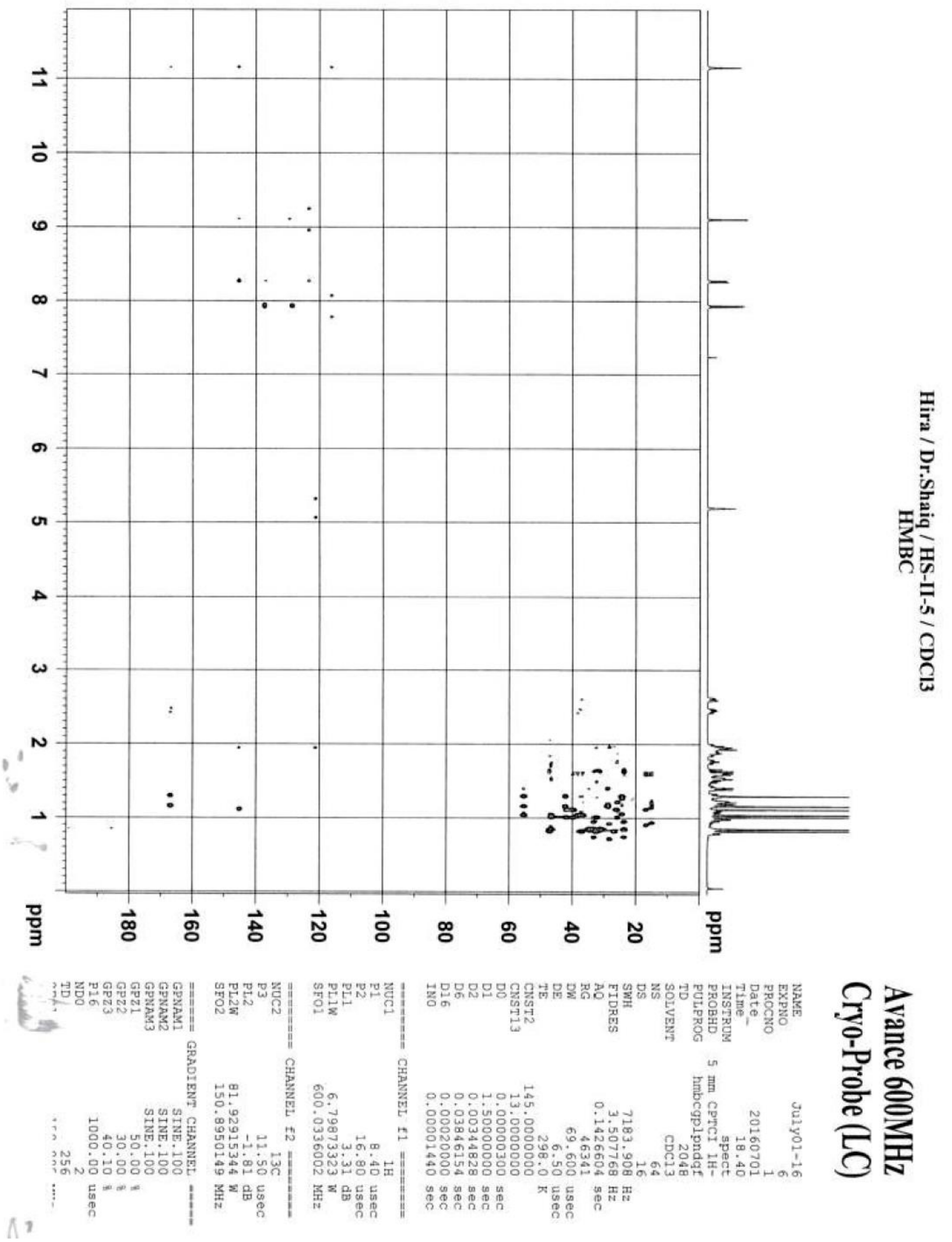

S8: HMBC spectrum of compound **2**

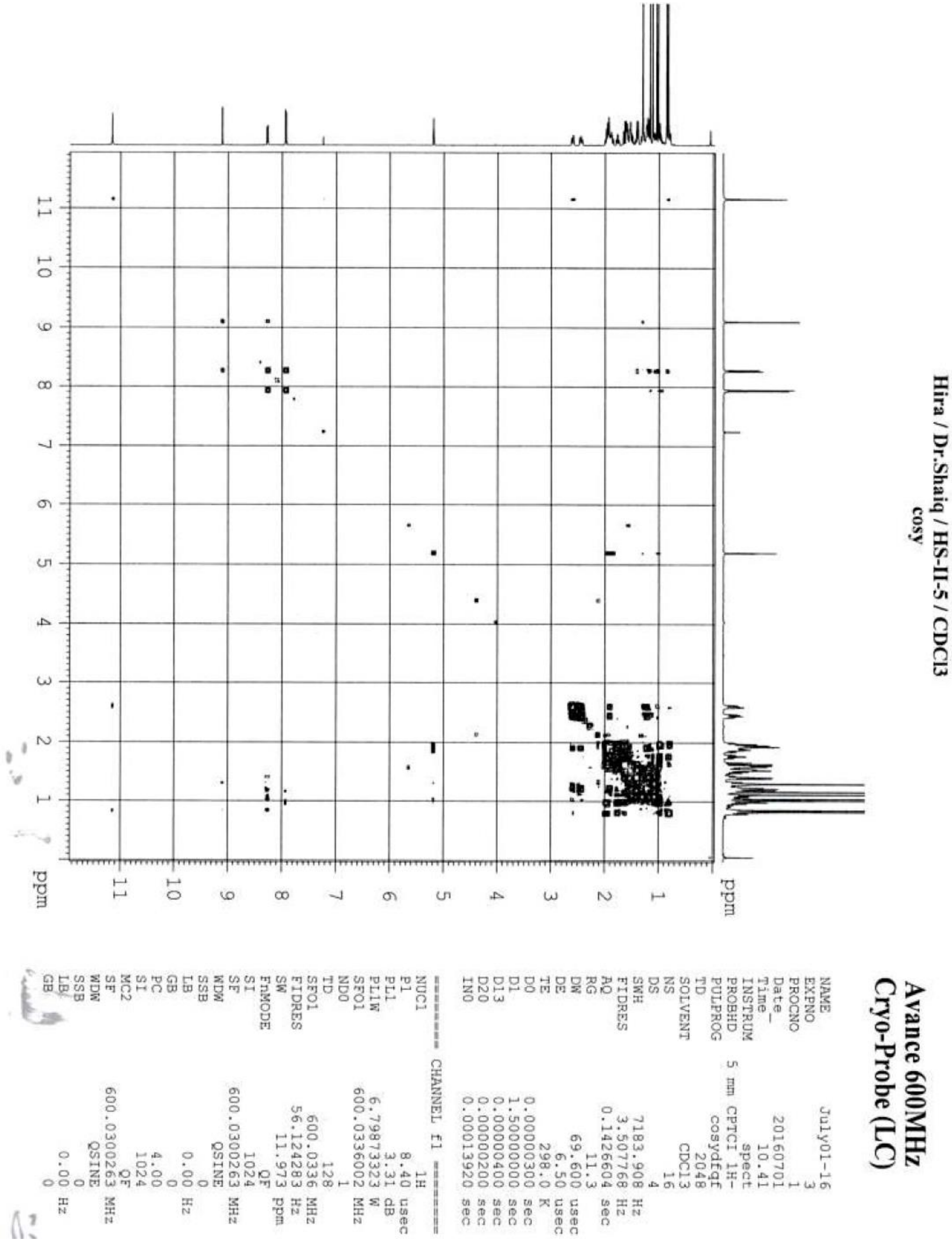

S9: COSY spectrum of compound **2**

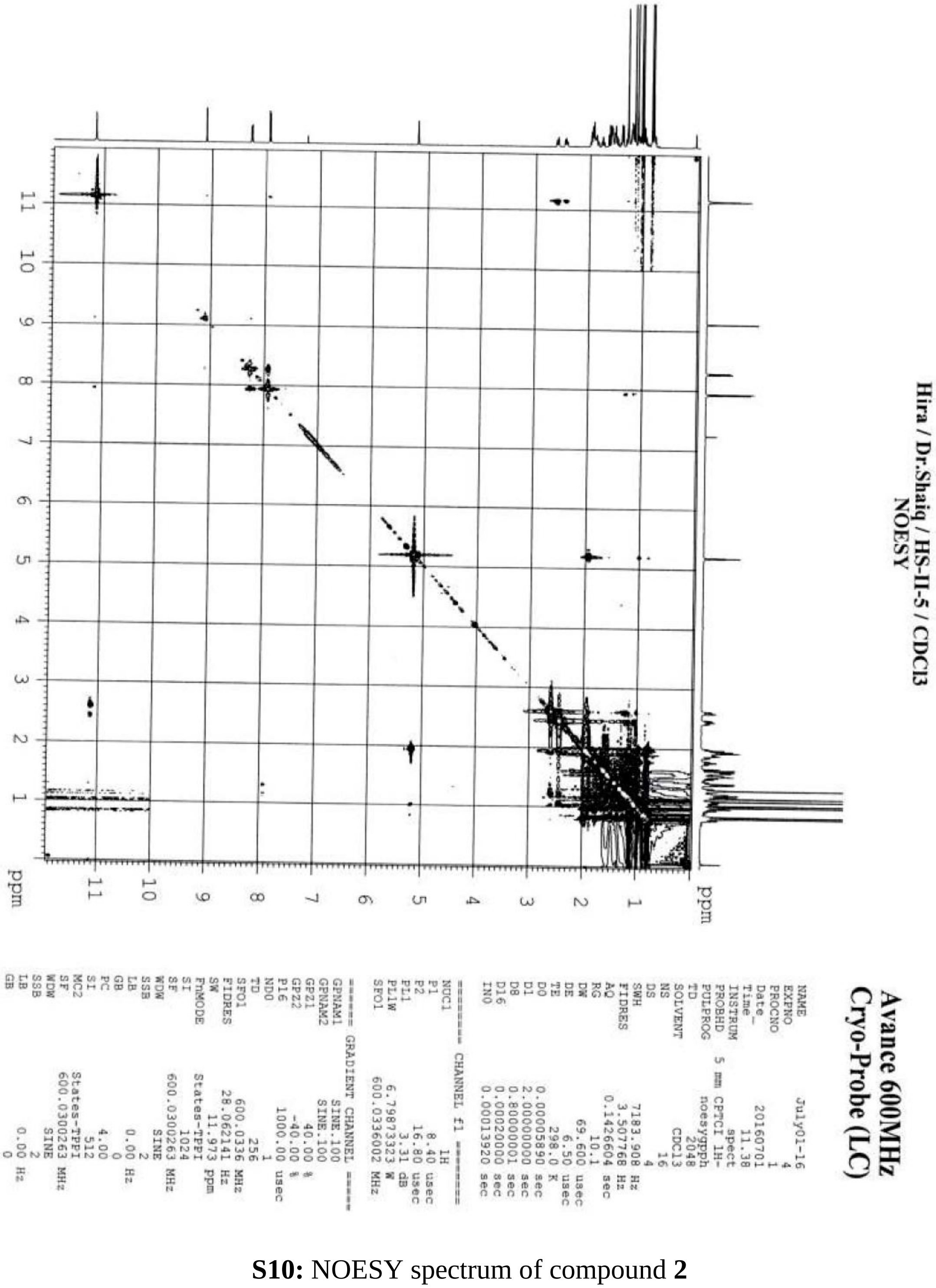

S10: NOESY spectrum of compound **2**

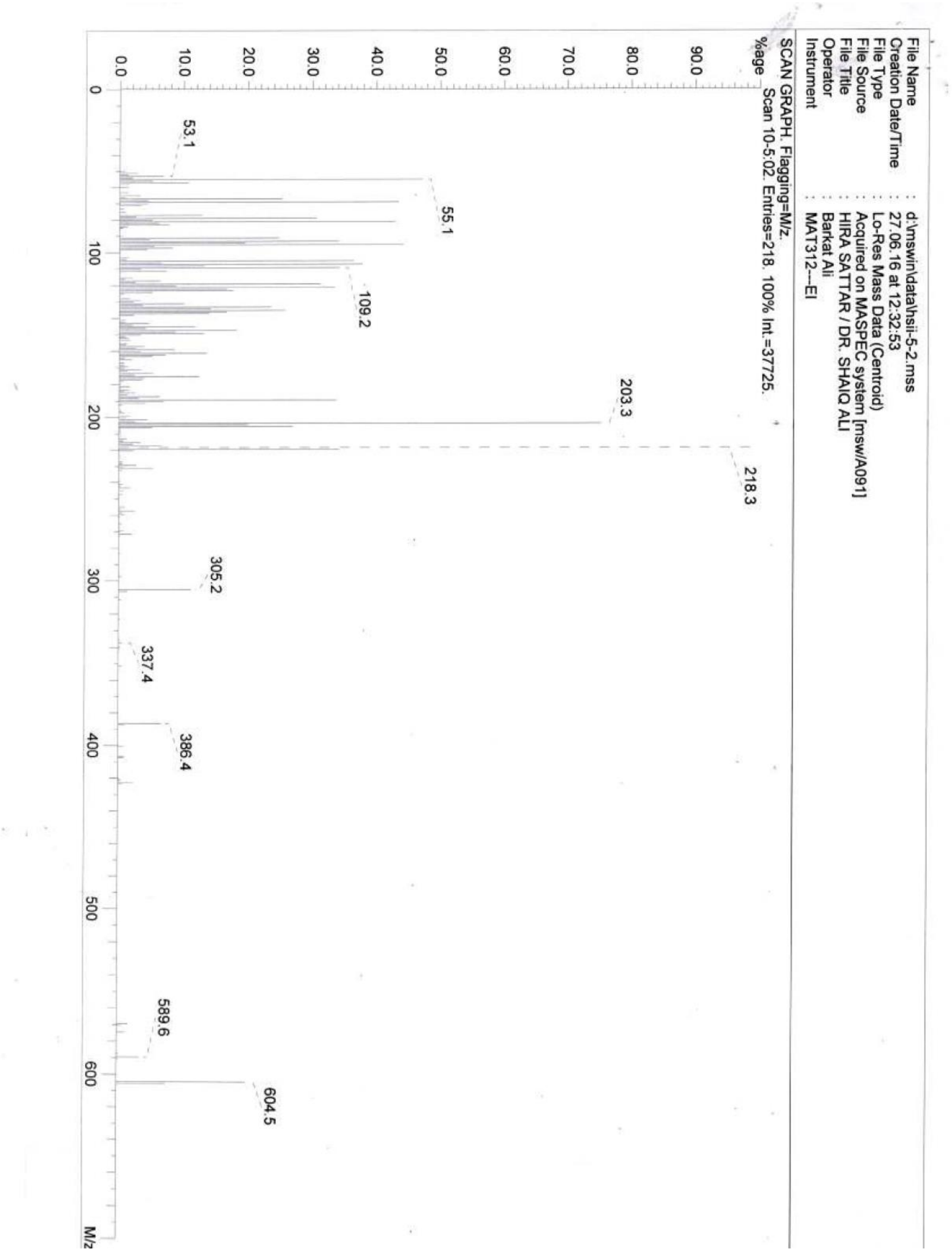

S11: EIMS spectrum of compound **2**

Mass	Relative Intensity	Theoretical Mass	Delta [ppm]	Delta [mmu]	RDB	Composition
		527.4087	9.2	4.8	8.0	$C_{32}H_{53}O_{3}N_{3}$
		527.4213	-14.7	-7.7	7.5	$C_{33}H_{55}O_{3}N_{2}$
528.42248	1.7	528.4205	3.7	1.9	11.5	$C_{37}H_{54}O_{1}N_{1}$
		528.4192	6.2	3.3	12.0	$C_{35}H_{52}N_{4}$
		528.4179	8.7	4.6	7.0	$C_{34}H_{56}O_{4}$
		528.4165	11.3	6.0	7.5	$C_{32}H_{54}O_{3}N_{3}$
		528.4291	-12.5	-6.6	7.0	$C_{33}H_{56}O_{3}N_{2}$
529.38329	1.7	529.3834	-0.3	-0.1	16.5	$C_{40}H_{49}$
		529.3794	7.3	3.9	12.5	$C_{35}H_{49}O_{2}N_{2}$
		529.3880	-8.8	-4.7	8.0	$C_{31}H_{51}O_{4}N_{3}$
		529.3906	-13.9	-7.4	12.5	$C_{34}H_{49}O_{1}N_{4}$
		529.3754	14.9	7.9	8.5	$C_{30}H_{49}O_{4}N_{4}$
540.42121	6.3	540.4205	1.2	0.7	12.5	$C_{38}H_{54}O_{1}N_{1}$
		540.4192	3.7	2.0	13.0	$C_{36}H_{52}N_{4}$
		540.4179	6.2	3.4	8.0	$C_{35}H_{56}O_{4}$
		540.4165	8.7	4.7	8.5	$C_{33}H_{54}O_{3}N_{3}$
		540.4291	-14.6	-7.9	8.0	$C_{34}H_{56}O_{3}N_{2}$
541.42076	3.4	541.4243	-6.6	-3.6	8.0	$C_{33}H_{55}O_{3}N_{3}$
		541.4257	-9.1	-4.9	7.5	$C_{35}H_{57}O_{4}$
		541.4158	9.2	5.0	12.5	$C_{37}H_{53}O_{1}N_{2}$
		541.4270	-11.6	-6.3	12.5	$C_{36}H_{53}N_{4}$
		541.4284	-14.0	-7.6	12.0	$C_{38}H_{55}O_{1}N_{1}$
542.40589	2.5	542.4084	-4.5	-2.5	8.0	$C_{33}H_{54}O_{4}N_{2}$
		542.4110	-9.5	-5.1	12.5	$C_{36}H_{52}O_{1}N_{3}$
		542.3998	11.2	6.1	12.5	$C_{37}H_{52}O_{2}N_{1}$
		542.4124	-12.0	-6.5	12.0	$C_{38}H_{54}O_{2}$
		542.3985	13.7	7.4	13.0	$C_{35}H_{50}O_{1}N_{4}$
543.40162	1.3	543.4036	-3.7	-2.0	8.0	$C_{32}H_{53}O_{4}N_{3}$
		543.3991	4.7	2.5	16.5	$C_{41}H_{51}$
		543.4063	-8.6	-4.7	12.5	$C_{35}H_{51}O_{1}N_{4}$
		543.4076	-11.1	-6.0	12.0	$C_{37}H_{53}O_{2}N_{1}$
		543.3951	12.1	6.6	12.5	$C_{36}H_{51}O_{2}N_{2}$
544.41167	3.3	544.4114	0.4	0.2	7.5	$C_{32}H_{54}O_{4}N_{3}$
		544.4141	-4.5	-2.4	12.0	$C_{35}H_{52}O_{1}N_{4}$
		544.4155	-6.9	-3.8	11.5	$C_{37}H_{54}O_{2}N_{1}$
		544.4069	8.8	4.8	16.0	$C_{41}H_{52}$
		544.4029	16.2	8.8	12.0	$C_{36}H_{52}O_{2}N_{2}$
545.40750	1.4	545.4067	1.5	0.8	7.5	$C_{31}H_{53}O_{4}N_{4}$
		545.4107	-5.9	-3.2	11.5	$C_{36}H_{53}O_{2}N_{2}$
		545.4022	9.8	5.3	16.0	$C_{40}H_{51}N_{1}$
		545.4147	-13.3	-7.2	15.5	$C_{41}H_{53}$
		545.3995	14.7	8.0	11.5	$C_{37}H_{53}O_{3}$
569.40196	1.1	569.4022	-0.3	-0.2	18.0	$C_{42}H_{51}N_{1}$
		569.3995	4.4	2.5	13.5	$C_{39}H_{53}O_{3}$
		569.3981	6.7	3.8	14.0	$C_{37}H_{51}O_{2}N_{3}$
		569.4067	-8.3	-4.7	9.5	$C_{33}H_{53}O_{4}N_{4}$
		569.4107	-15.4	-8.7	13.5	$C_{38}H_{53}O_{2}N_{2}$
574.45263	2.3	574.4539	-2.1	-1.2	15.0	$C_{43}H_{58}$
		574.4498	4.9	2.8	11.0	$C_{38}H_{58}O_{2}N_{2}$
		574.4584	-10.0	-5.8	6.5	$C_{34}H_{60}O_{4}N_{3}$
		574.4458	11.9	6.8	7.0	$C_{33}H_{58}O_{4}N_{4}$
		574.4611	-14.7	-8.4	11.0	$C_{37}H_{58}O_{1}N_{4}$
589.40748	2.4	589.4046	5.0	2.9	16.5	$C_{42}H_{53}O_{2}$
		589.4032	7.2	4.3	17.0	$C_{40}H_{51}O_{1}N_{3}$
		589.4118	-7.3	-4.3	12.5	$C_{36}H_{53}O_{3}N_{4}$
		589.4131	-9.5	-5.6	12.0	$C_{38}H_{55}O_{4}N_{1}$
		589.4005	11.8	6.9	12.5	$C_{37}H_{53}O_{4}N_{2}$
604.40220	6.3	604.4029	-1.1	-0.7	17.0	$C_{41}H_{52}O_{2}N_{2}$
		604.3989	5.5	3.3	13.0	$C_{36}H_{52}O_{4}N_{4}$
		604.4069	-7.8	-4.7	21.0	$C_{46}H_{52}$
		604.3943	13.0	7.9	21.5	$C_{45}H_{50}N_{1}$
		604.4114	-15.3	-9.2	12.5	$C_{37}H_{54}O_{4}N_{3}$
605.42198	2.4	605.4219	0.1	0.0	16.5	$C_{40}H_{53}O_{1}N_{4}$

S12: HR-EIMS spectrum of compound **2**

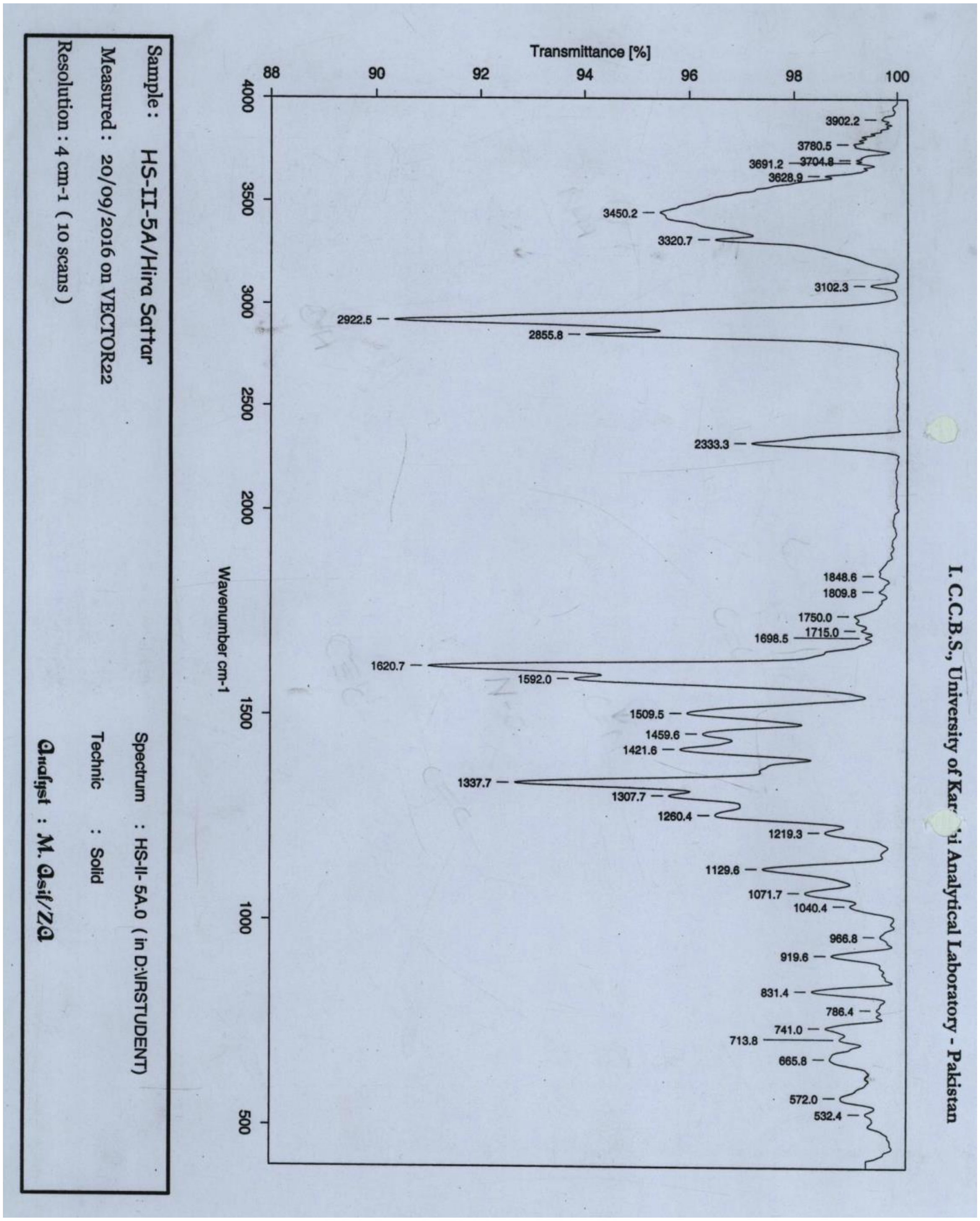

S13: IR spectrum of compound **2**

THERMO ELECTRON ~ VISIONpro SOFTWARE V4.10

Operator Name	ARSHAD ALAM	Date of Report	4/27/2018
Department	Analytical Laboratory TWC # 004	Time of Report	12:23:17AM
Organization	ICCBS Karachi of Universty.		
Information	Prof.Dr.M.Shaiq Ali./ Hira Sattar.		

Scan Graph

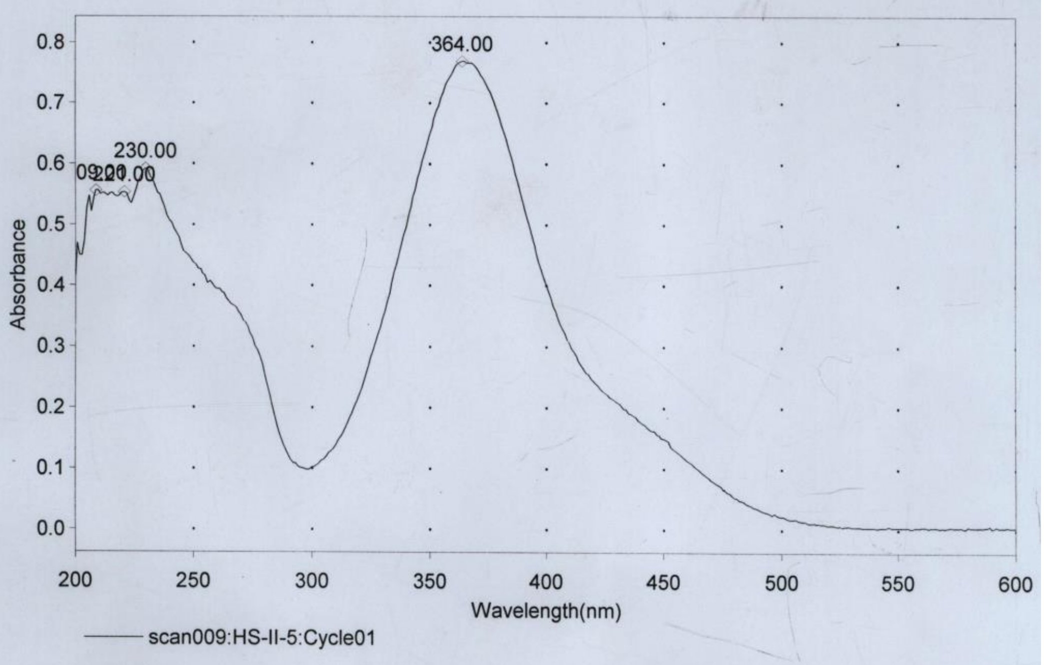

Results Table - scan009,HS-II-5,Cycle01

nm	A	**Peak Pick Method**
209.00	0.556	Find 4 Peaks Above -3.0000 A
221.00	0.553	Start Wavelength 200.00 nm
230.00	0.592	Stop Wavelength 600.00 nm
364.00	0.769	Sort By Wavelength
Sensitivity	Medium	

S14: UV spectrum of compound **2**

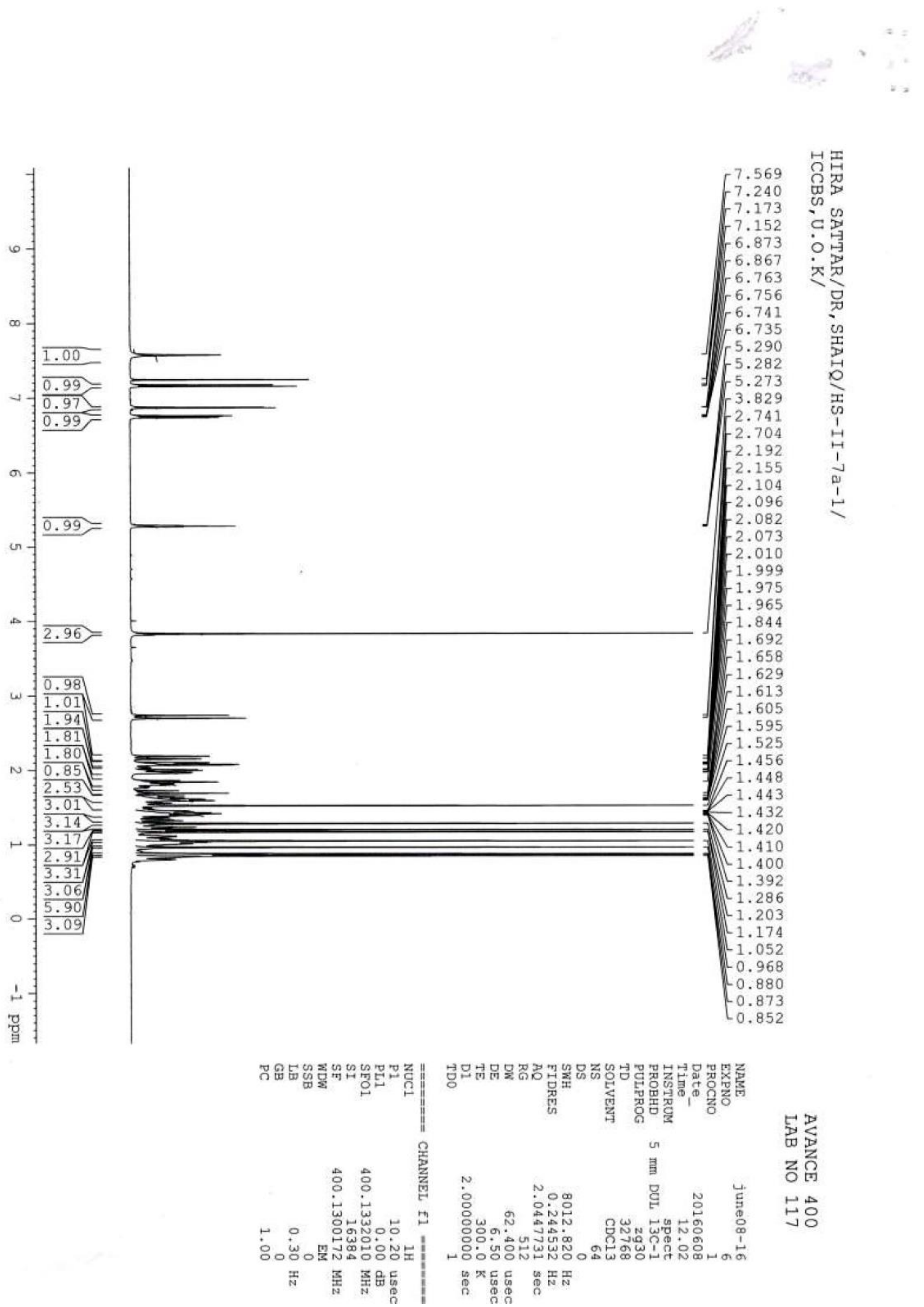
HIRA SATTAR/DR,SHAIQ/HS-II-7a-1/
ICCBS,U.O.K/
7.569
7.240
7.173
7.152
6.873
6.867
6.763
6.756
6.741
6.735
5.290
5.282
5.273
3.829
2.741
2.704
2.192
2.155
2.104
2.096
2.082
2.073
2.010
1.999
1.975
1.965
1.844
1.692
1.658
1.629
1.613
1.605
1.595
1.525
1.456
1.448
1.443
1.432
1.420
1.410
1.400
1.392
1.286
1.203
1.174
1.052
0.968
0.880
0.873
0.852
1.00
0.99
0.97
0.99
0.99
2.96
0.98
1.01
1.94
1.81
1.80
0.85
2.53
3.01
3.14
3.17
2.91
3.31
3.06
5.90
3.09
9
8
7
6
5
4
3
2
1
0
-1 ppm
AVANCE 400
LAB NO 117
NAME june08-16
EXPNO 6
PROCNO 1
Date_ 20160608
Time 12.02
INSTRUM spect
PROBHD 5 mm DUL 13C-1
PULPROG zg30
TD 32768
SOLVENT CDCl3
NS 64
DS 0
SWH 8012.820 Hz
FIDRES 0.244532 Hz
AQ 2.0447731 sec
RG 512
DW 62.400 usec
DE 6.50 usec
TE 300.0 K
D1 2.00000000 sec
TD0 1
======== CHANNEL f1 ========
NUC1 1H
P1 10.20 usec
PL1 0.00 dB
SFO1 400.1332010 MHz
SI 16384
SF 400.1300172 MHz
WDW EM
SSB 0
LB 0.30 Hz
GB 0
PC 1.00

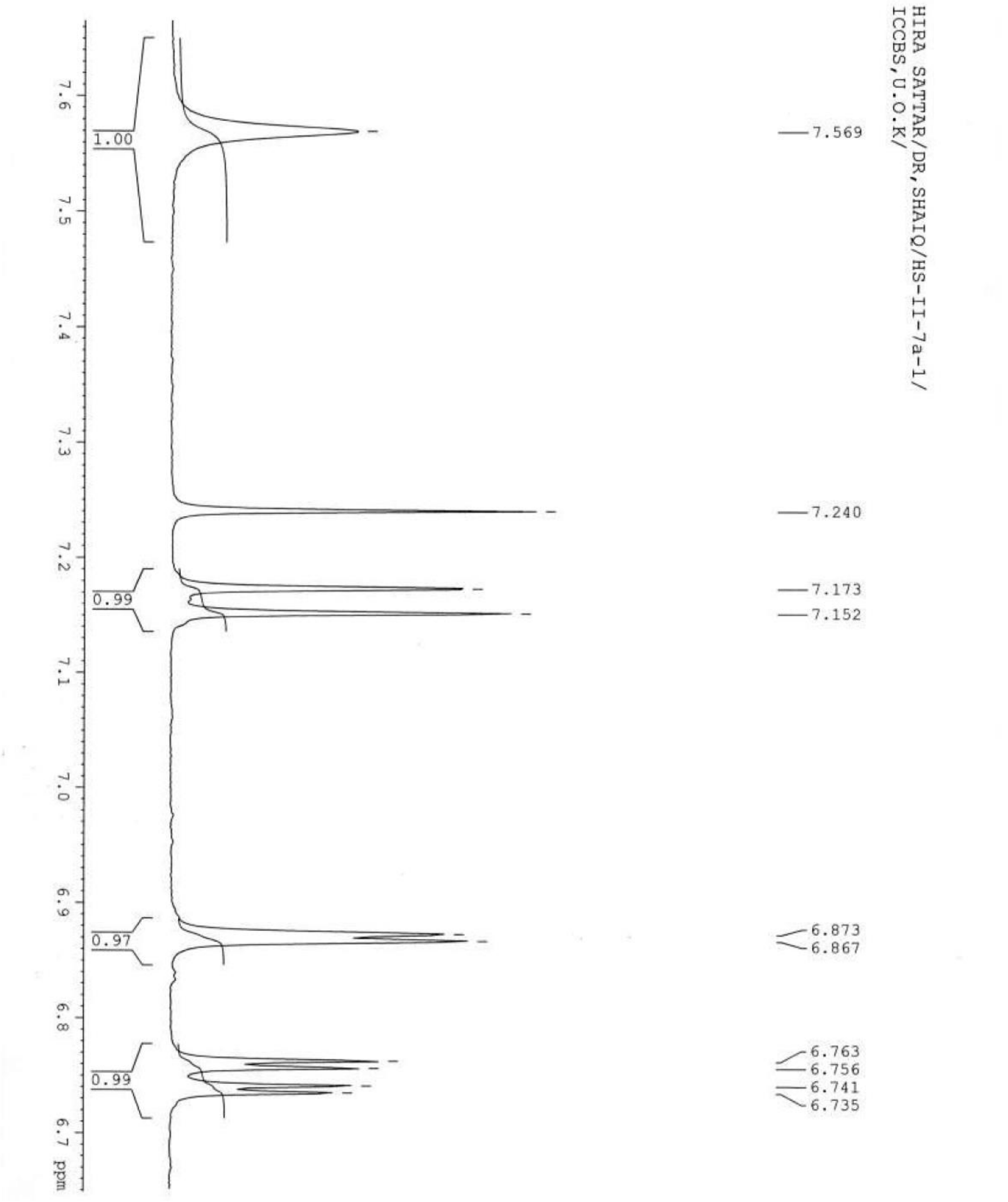
HIRA SATTAR/DR,SHAIQ/HS-II-7a-1/
ICCBS,U.O.K/
7.569
7.240
7.173
7.152
6.873
6.867
6.763
6.756
6.741
6.735
1.00
0.99
0.97
0.99
7.6
7.5
7.4
7.3
7.2
7.1
7.0
6.9
6.8
6.7 ppm

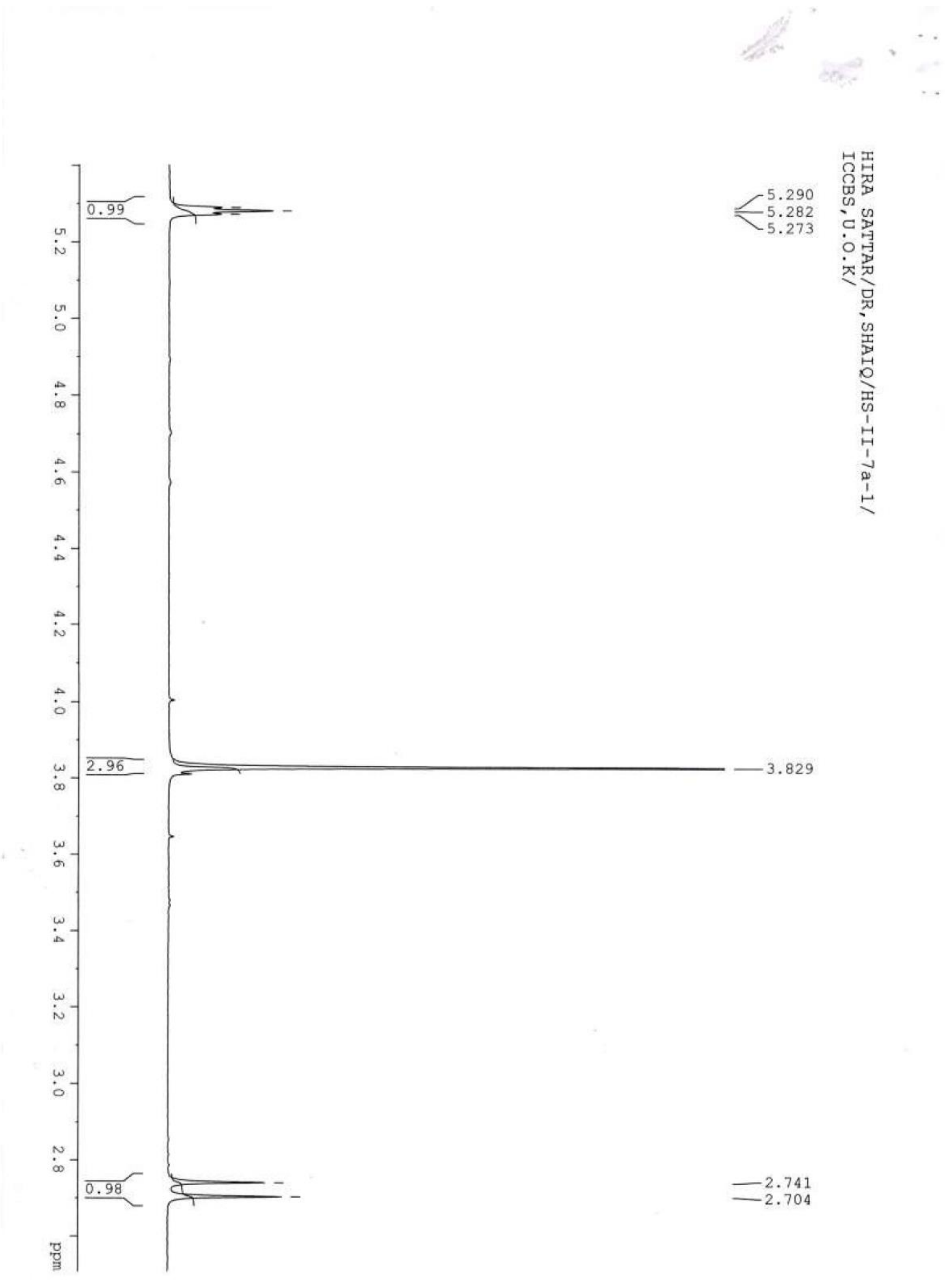
HIRA SATTAR/DR,SHAIQ/HS-II-7a-1/
ICCBS,U.O.K/
5.290
5.282
5.273
3.829
2.741
2.704
0.99
2.96
0.98
5.2
5.0
4.8
4.6
4.4
4.2
4.0
3.8
3.6
3.4
3.2
3.0
2.8
ppm

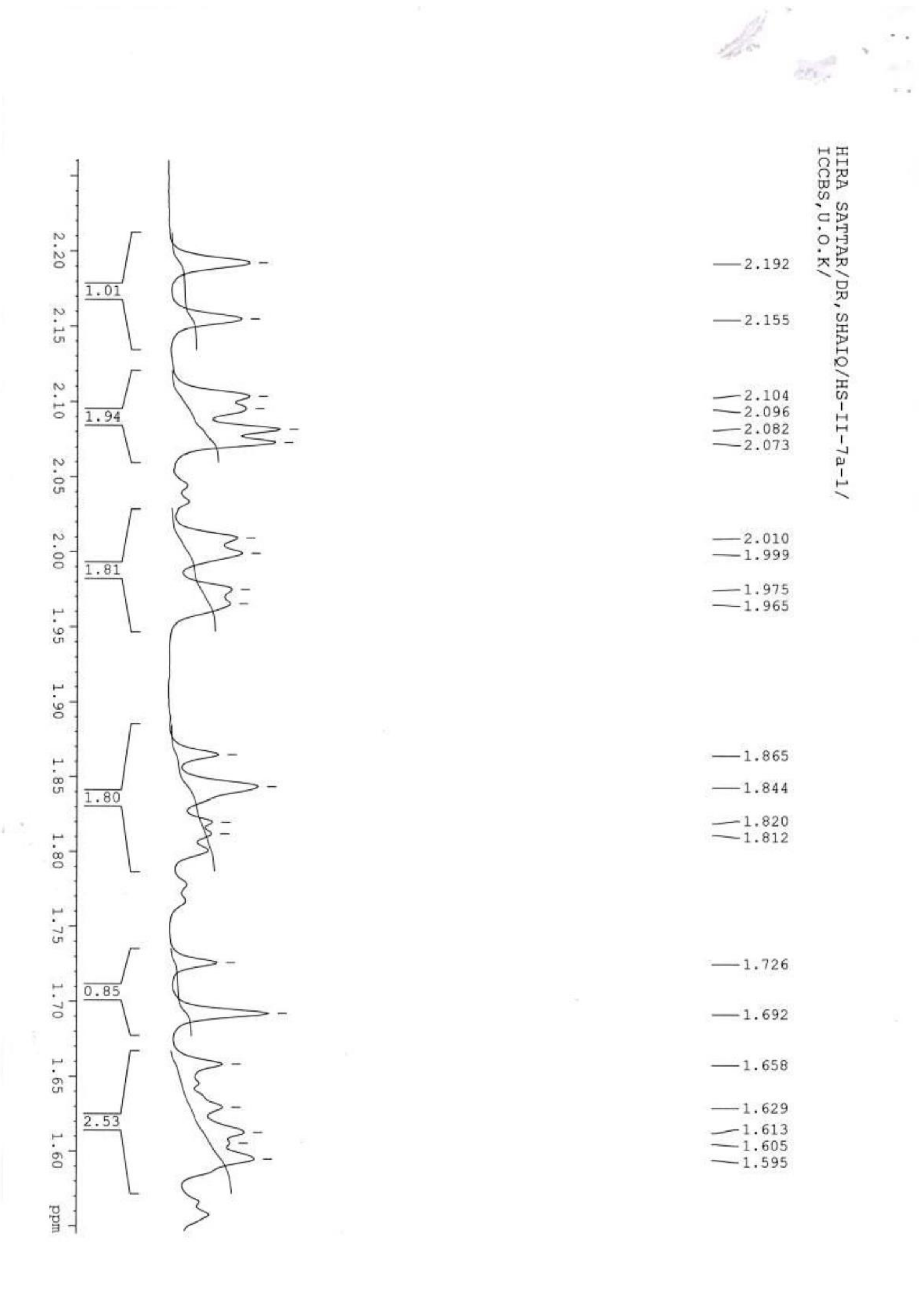
HIRA SATTAR/DR,SHAIQ/HS-II-7a-1/
ICCBS,U.O.K/
2.192
2.155
2.104
2.096
2.082
2.073
2.010
1.999
1.975
1.965
1.865
1.844
1.820
1.812
1.726
1.692
1.658
1.629
1.613
1.605
1.595
1.01
1.94
1.81
1.80
0.85
2.53
2.20
2.15
2.10
2.05
2.00
1.95
1.90
1.85
1.80
1.75
1.70
1.65
1.60
ppm

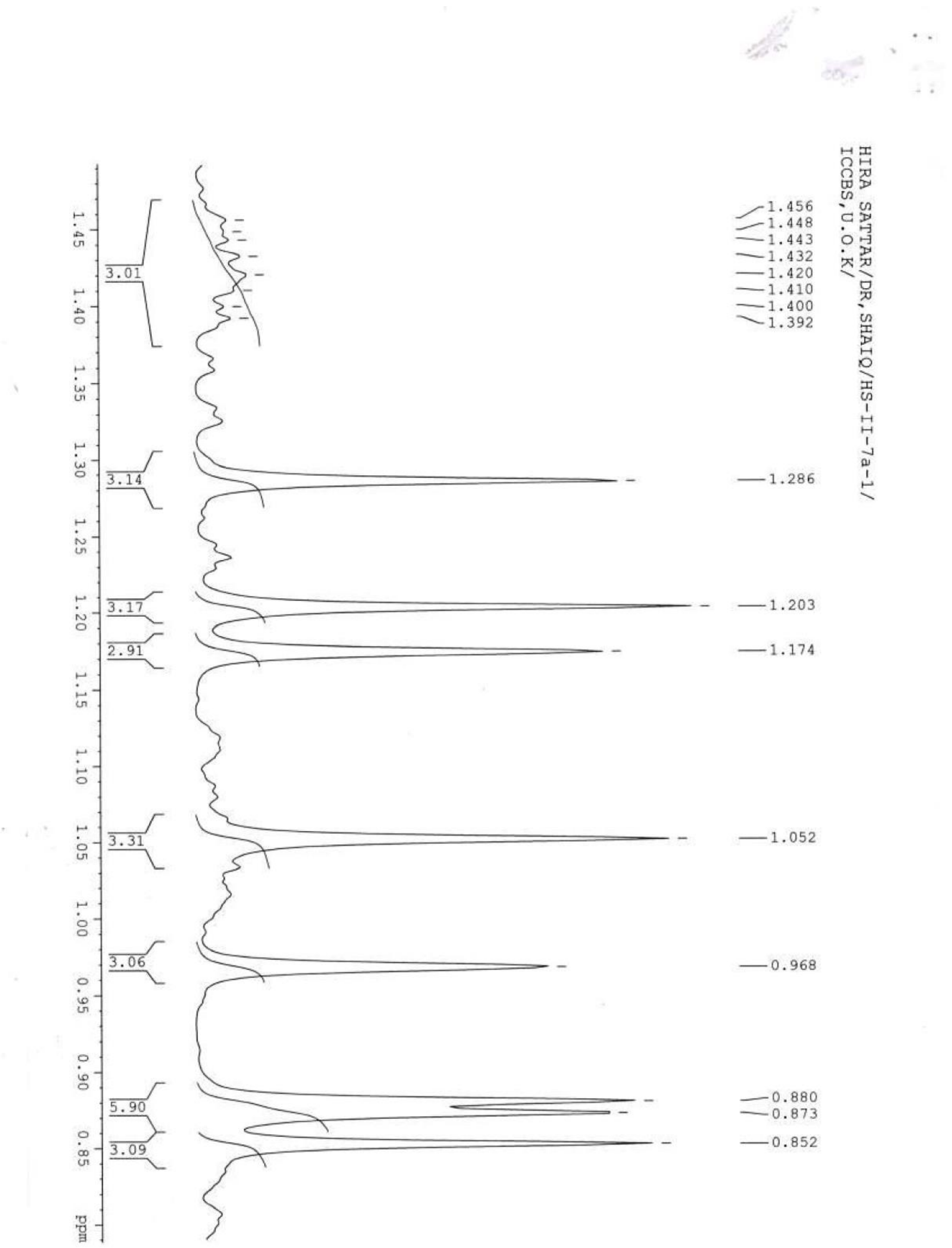

S15: ^{1}H-NMR spectrum of compound **3**

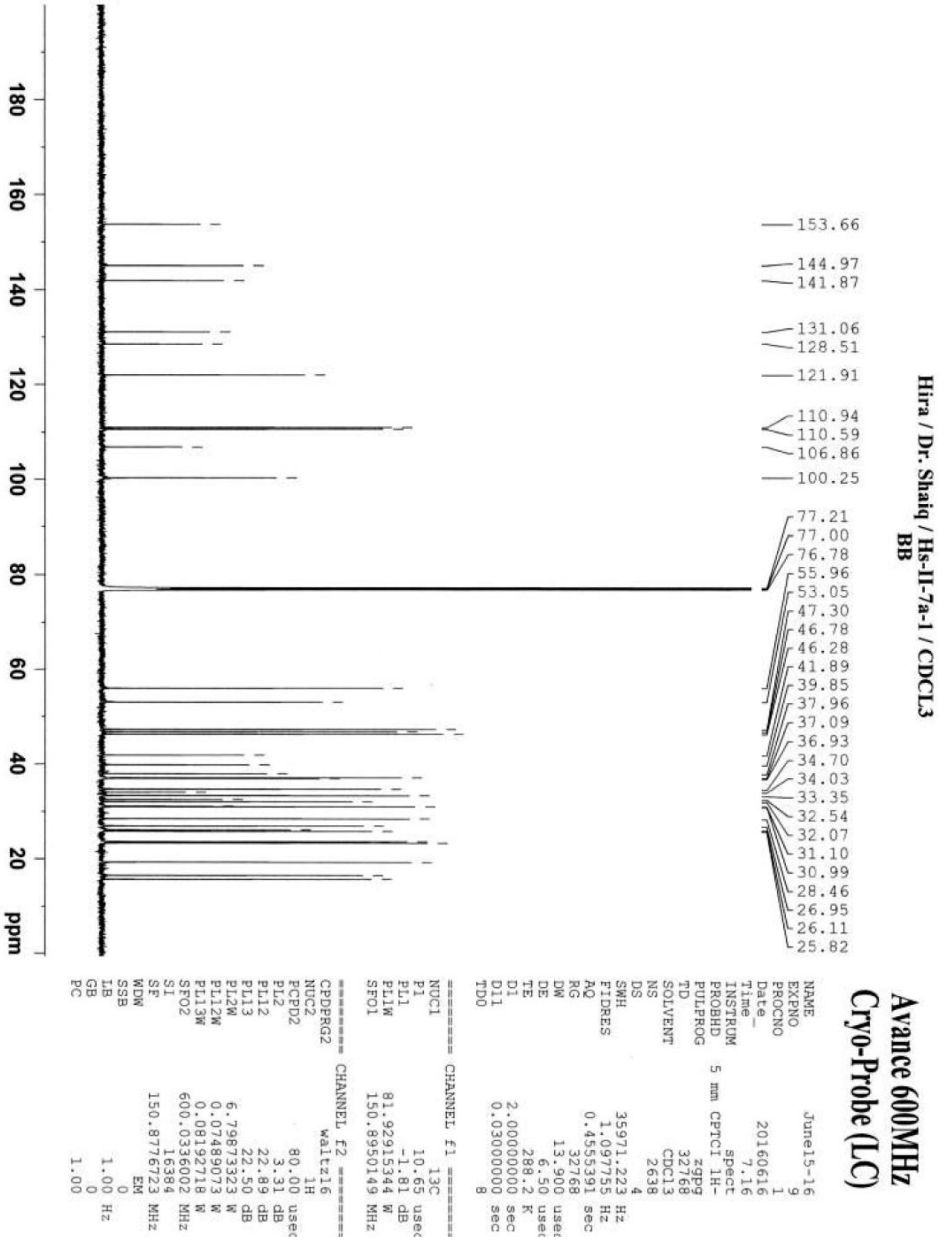

S16: ^{13}C-NMR spectrum of compound **3**

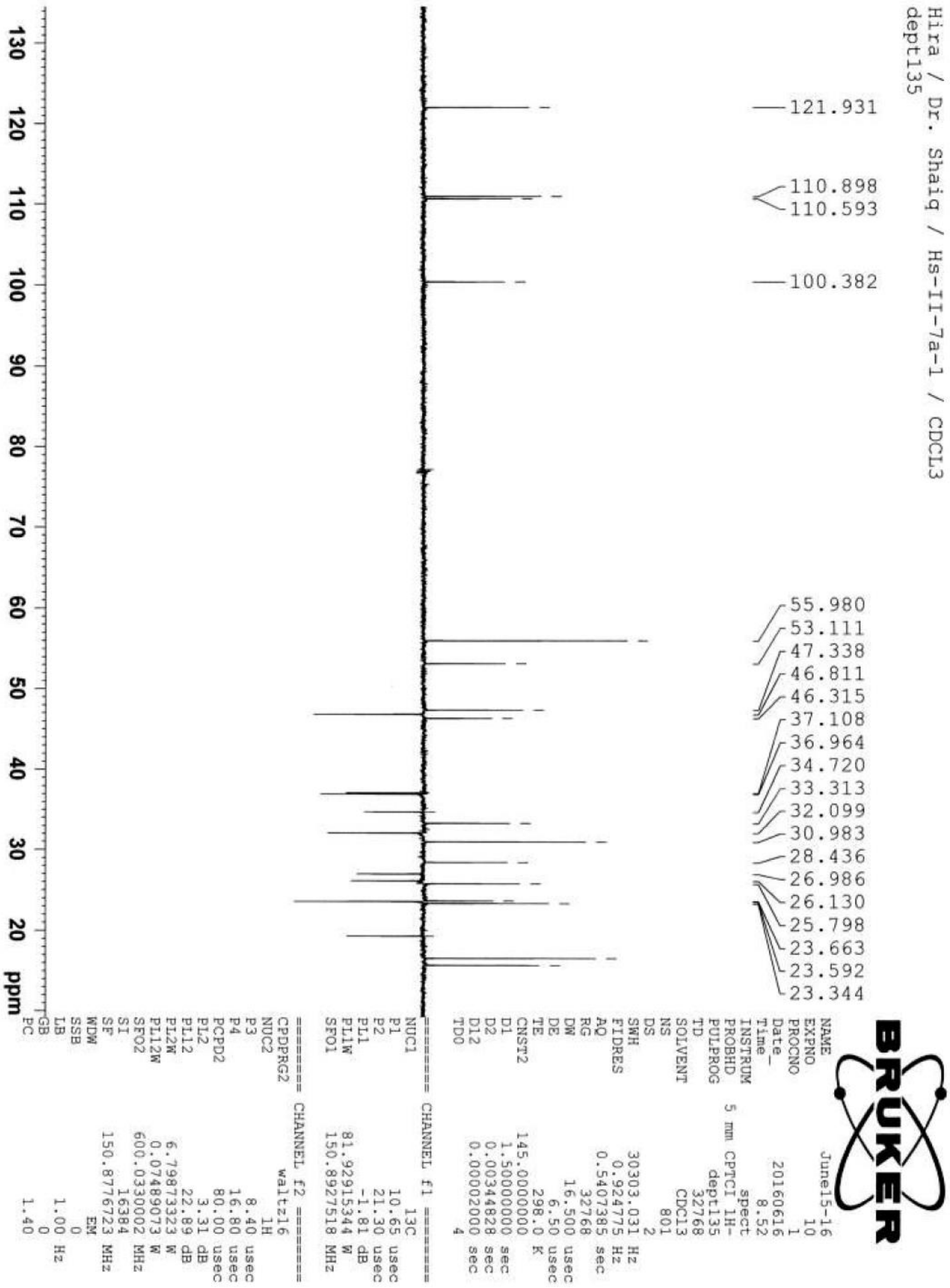

S17: ^{13}C DEPT-135 spectrum of compound **3**

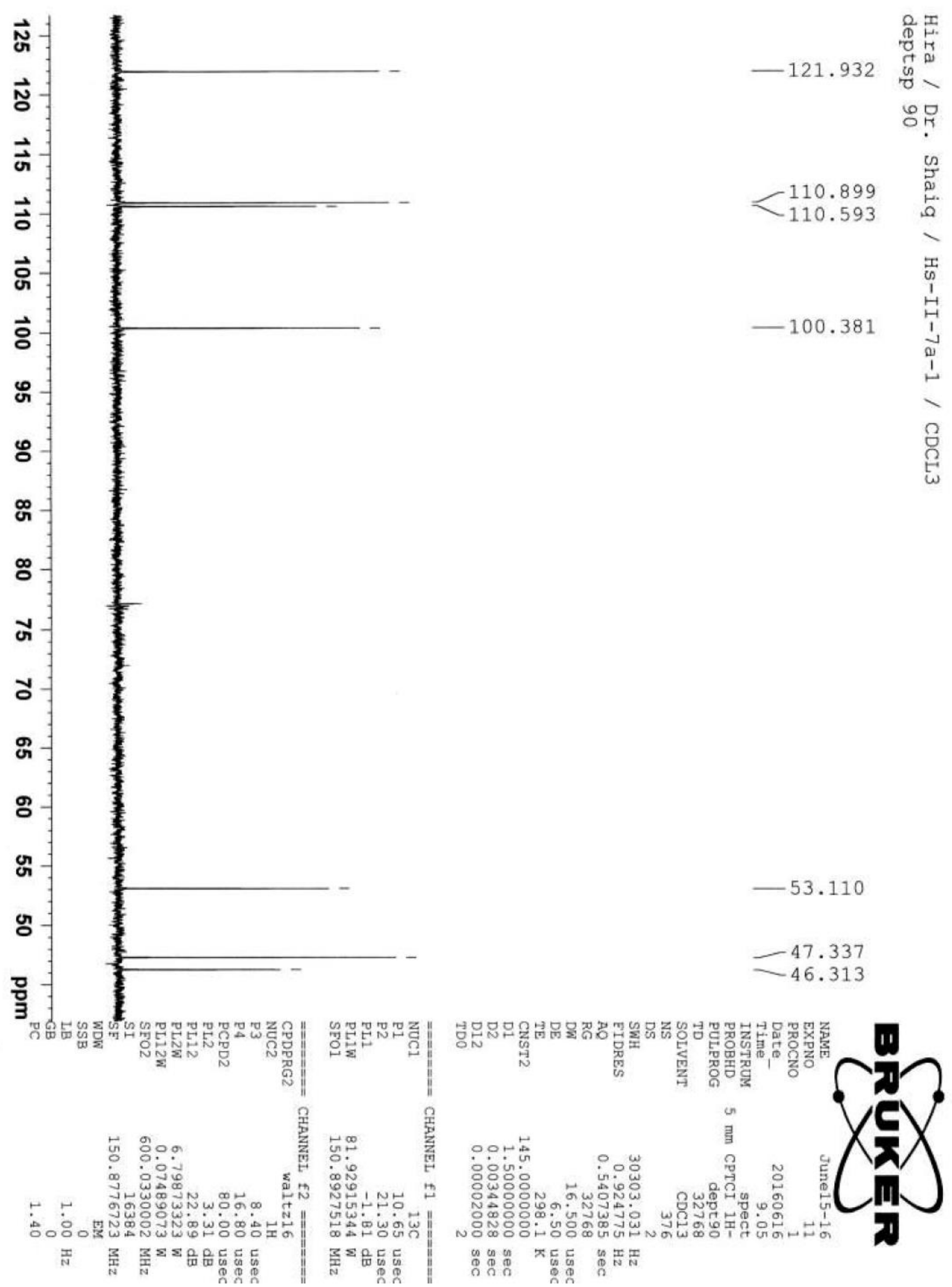

S18: ^{13}C DEPT-90 spectrum of compound **3**

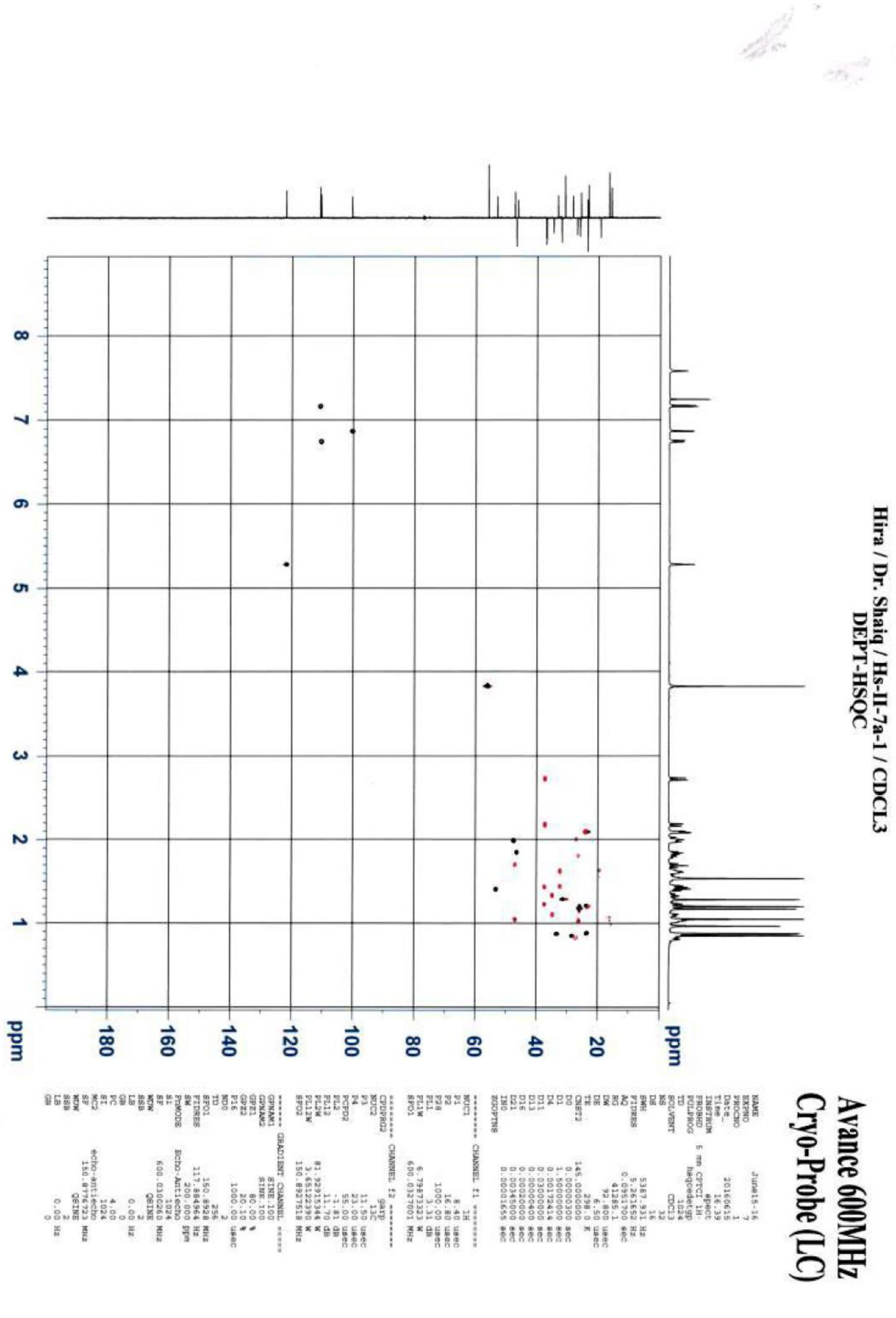
Hira / Dr. Shaiq / Hs-II-7a-1 / CDCL3
DEPT-HSQC
Avance 600MHz
Cryo-Probe (LC)
ppm
ppm
NAME June15-16
EXPNO 7
PROCNO 1
Date_ 20160615
Time 16.39
INSTRUM spect
PROBHD 5 mm CPTCI 1H-
PULPROG hsqcedetgp
TD 1024
SOLVENT CDCl3
NS 32
DS 16
SWH 5387.931 Hz
FIDRES 5.261652 Hz
AQ 0.0951700 sec
RG 41285.1
DW 92.800 usec
DE 6.50 usec
TE 298.0 K
CNST2 145.0000000
D0 0.00000300 sec
D1 1.50000000 sec
D4 0.00172414 sec
D11 0.03000000 sec
D13 0.00000400 sec
D16 0.00020000 sec
D21 0.00345000 sec
IN0 0.00001655 sec
ZGOPTNS
======== CHANNEL f1 ========
NUC1 1H
P1 8.40 usec
P2 16.80 usec
P28 1000.00 usec
PL1 3.31 dB
PL1W 6.79873323 W
SFO1 600.0327001 MHz
======== CHANNEL f2 ========
CPDPRG2 garp
NUC2 13C
P3 11.50 usec
P4 23.00 usec
PCPD2 55.00 usec
PL2 -1.81 dB
PL12 11.70 dB
PL2W 81.92915344 W
PL12W 3.65122390 W
SFO2 150.8927518 MHz
====== GRADIENT CHANNEL =====
GPNAM1 SINE.100
GPNAM2 SINE.100
GPZ1 80.00 %
GPZ2 20.10 %
P16 1000.00 usec
ND0 2
TD 256
SFO1 150.8928 MHz
FIDRES 117.884964 Hz
SW 200.000 ppm
FnMODE Echo-Antiecho
SI 1024
SF 600.0300260 MHz
WDW QSINE
SSB 2
LB 0.00 Hz
GB 0
PC 4.00
SI 1024
MC2 echo-antiecho
SF 150.8776723 MHz
WDW QSINE
SSB 2
LB 0.00 Hz
GB 0

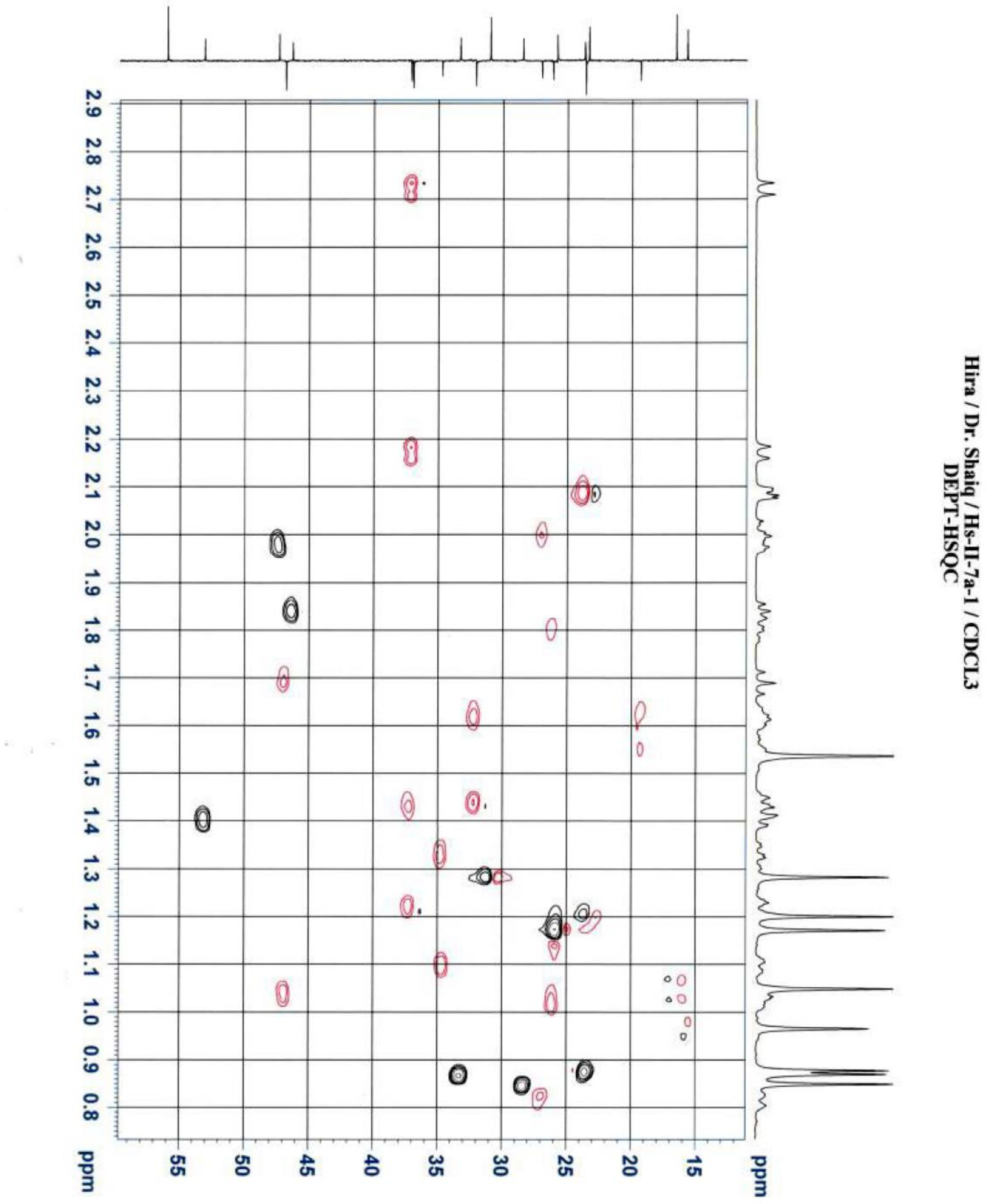

S19: DEPT-HSQC spectrum of compound **3**

Hira / Dr. Shaiq / Hs-II-7a-1 / CDCL3
HMBC

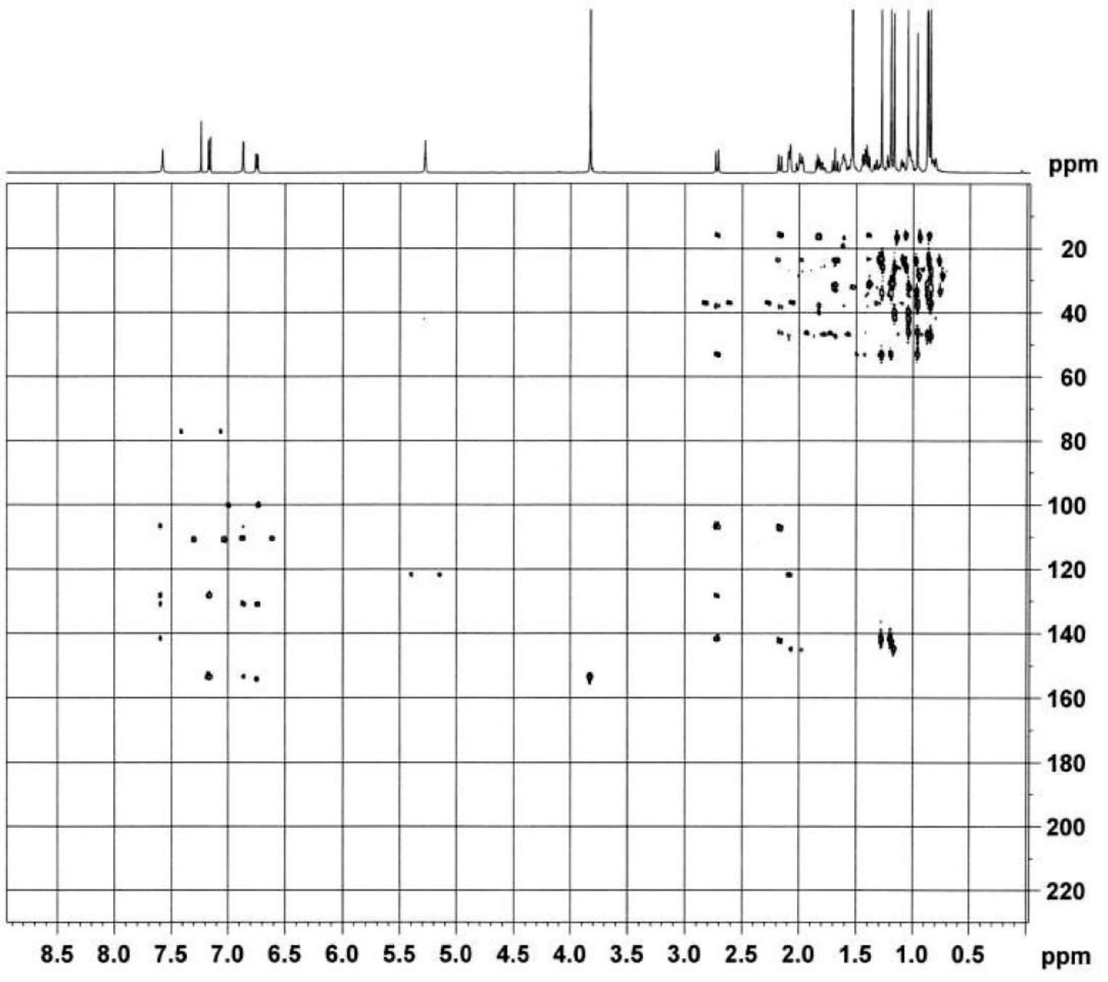

Avance 600MHz
Cryo-Probe (LC)

```
NAME          June15-16
EXPNO                 8
PROCNO                1
Date_          20160615
Time              20.22
INSTRUM           spect
PROBHD   5 mm CPTCI 1H-
PULPROG    hmbcgplpndqf
TD                 2048
SOLVENT           CDCl3
NS                   64
DS                   16
SWH            5387.931 Hz
FIDRES         2.630826 Hz
AQ            0.1901972 sec
RG              41285.1
DW               92.800 usec
DE                 6.50 usec
TE                298.0 K
CNST2       145.0000000
CNST13       13.0000000
D0           0.00000300 sec
D1           2.00000000 sec
D2           0.00344828 sec
D6           0.03846154 sec
D16          0.00020000 sec
IN0          0.00001440 sec

======== CHANNEL f1 ========
NUC1                 1H
P1                 8.40 usec
P2                16.80 usec
PL1                3.31 dB
PL1W         6.79873323 W
SFO1        600.0327001 MHz

======== CHANNEL f2 ========
NUC2                13C
P3                11.50 usec
PL2               -1.81 dB
PL2W        81.92915344 W
SFO2        150.8950149 MHz

====== GRADIENT CHANNEL =====
GPNAM1         SINE.100
GPNAM2         SINE.100
GPNAM3         SINE.100
GPZ1              50.00 %
GPZ2              30.00 %
GPZ3              40.10 %
P16             1000.00 usec
ND0                   2
TD                  256
SFO1            150.895 MHz
FIDRES       135.569733 Hz
SW              230.000 ppm
FnMODE               QF
SI                 1024
SF          600.0300260 MHz
WDW                SINE
SSB                   0
LB                 0.00 Hz
GB                    0
PC                 4.00
SI                 1024
MC2                  QF
SF          150.8776723 MHz
WDW                SINE
SSB                   0
LB                 0.00 Hz
GB                    0
```

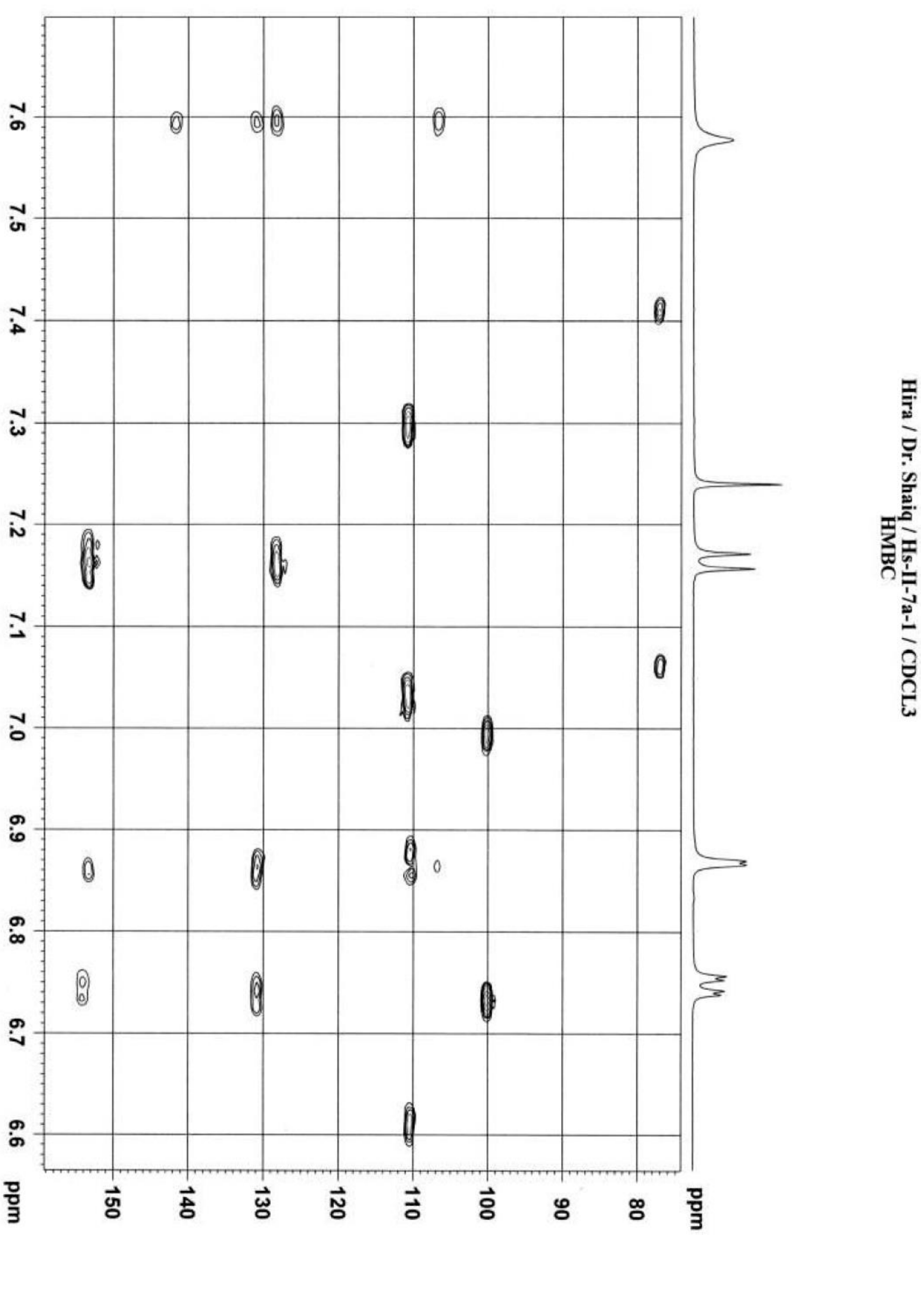
Hira / Dr. Shaiq / Hs-II-7a-1 / CDCL3
HMBC
ppm
ppm

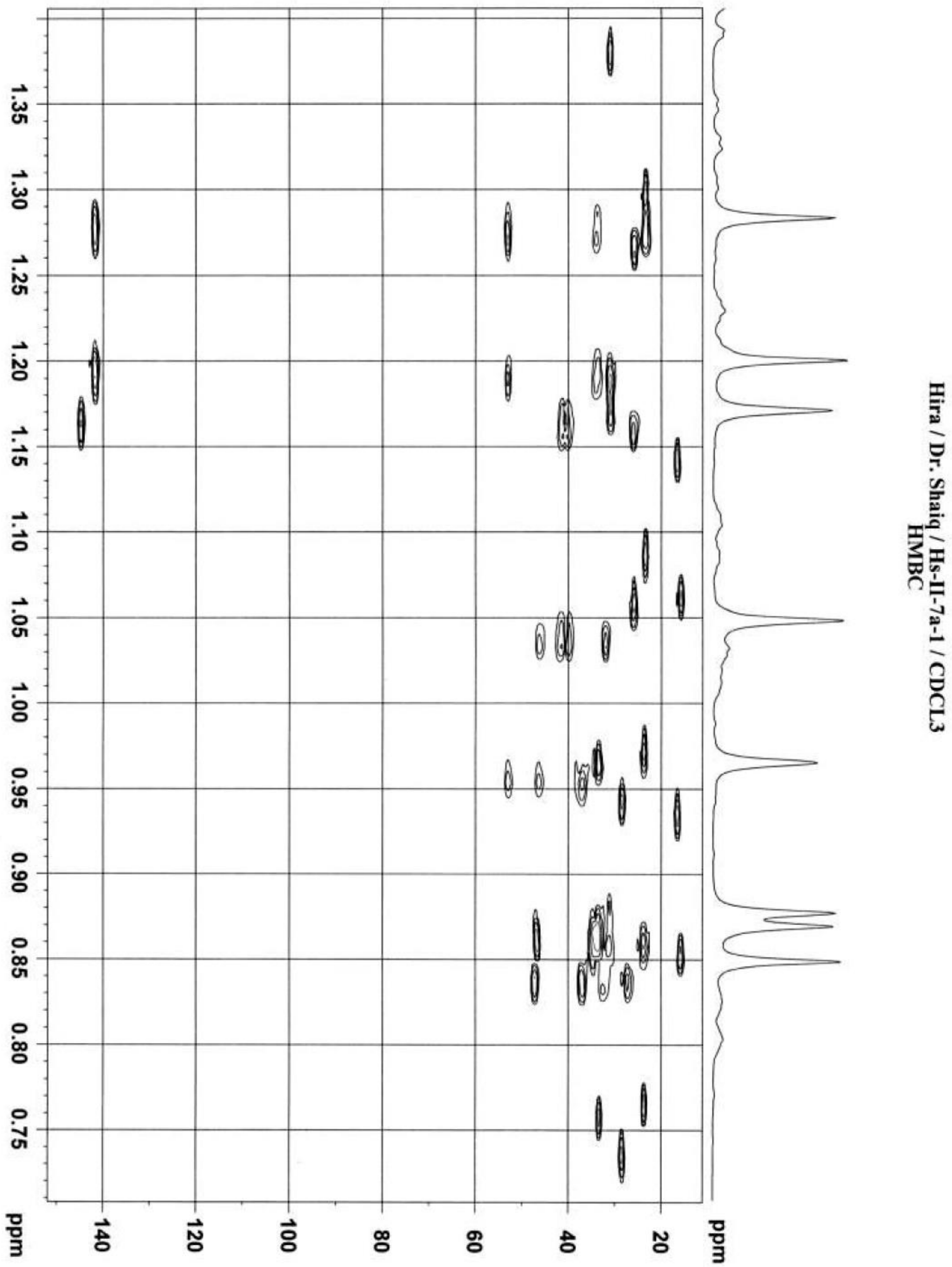

S20: HMBC spectrum of compound **3**

Hira / Dr. Shaiq / Hs-II-7a-1 / CDCL3
cosy

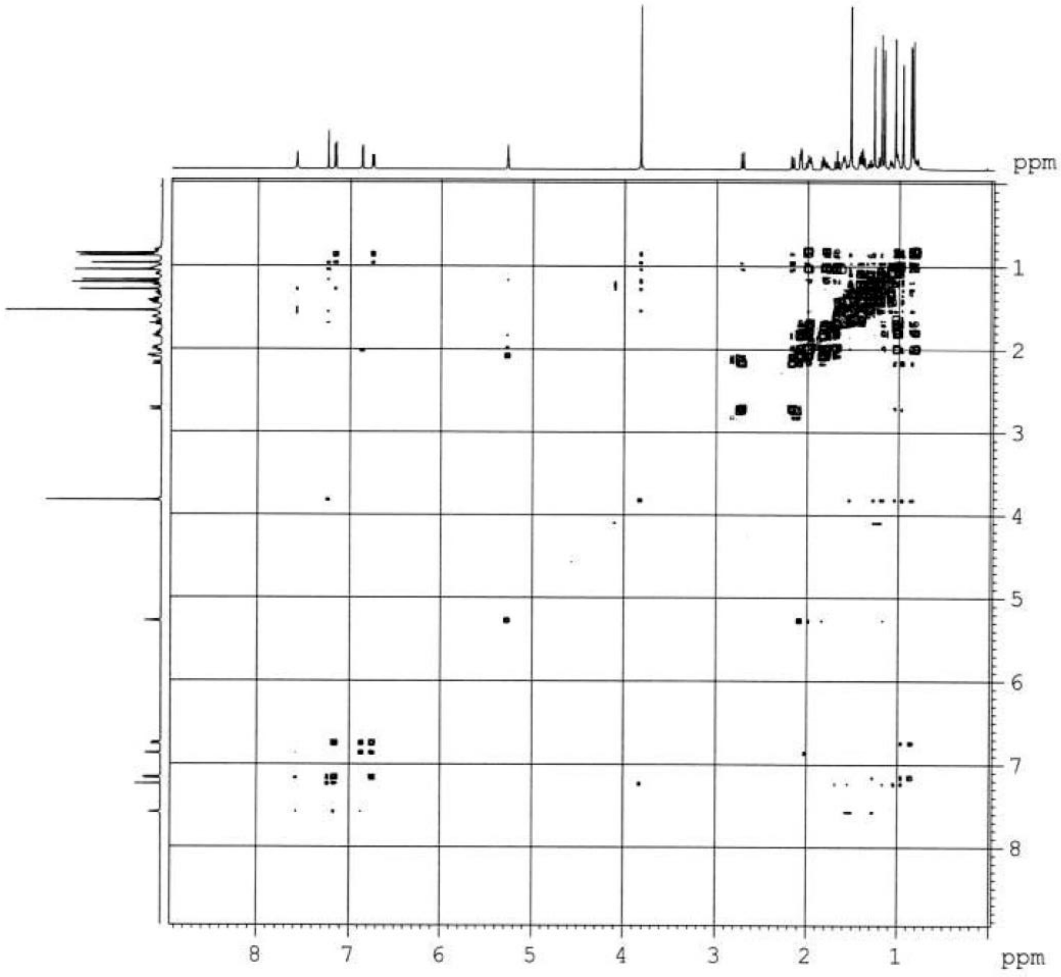

Avance 600MHz Cryo-Probe (LC)

NAME	June15-16
EXPNO	5
PROCNO	1
Date_	20160617
Time	10.36
INSTRUM	spect
PROBHD	5 mm CPTCI 1H-
PULPROG	cosydfqf
TD	2048
SOLVENT	CDCl3
NS	16
DS	16
SWH	5387.931 Hz
FIDRES	2.630826 Hz
AQ	0.1901972 sec
RG	40.3
DW	92.800 usec
DE	6.50 usec
TE	298.0 K
D0	0.00000300 sec
D1	2.00000000 sec
D13	0.00000400 sec
D20	0.00000200 sec
IN0	0.00018560 sec
======== CHANNEL f1 ========	
NUC1	1H
P1	8.40 usec
PL1	3.31 dB
PL1W	6.79873323 W
SFO1	600.0327001 MHz
ND0	1
TD	128
SFO1	600.0327 MHz
FIDRES	42.093212 Hz
SW	8.979 ppm
FnMODE	QF
SI	1024
SF	600.0300260 MHz
WDW	QSINE
SSB	0
LB	0.00 Hz
GB	0
PC	4.00
SI	1024
MC2	QF
SF	600.0300260 MHz
WDW	QSINE
SSB	0
LB	0.00 Hz
GB	0

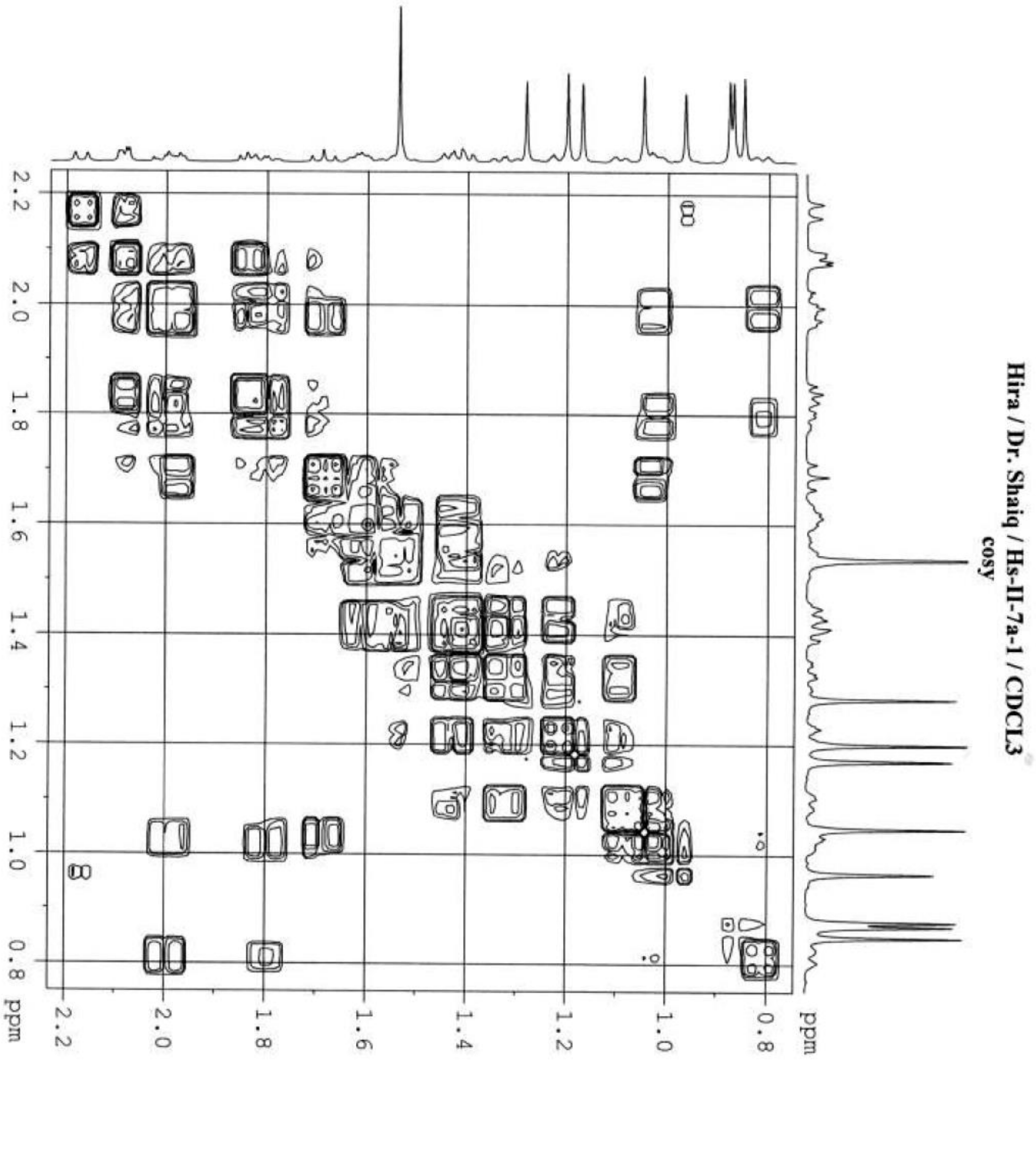

S21: COSY spectrum of compound **3**

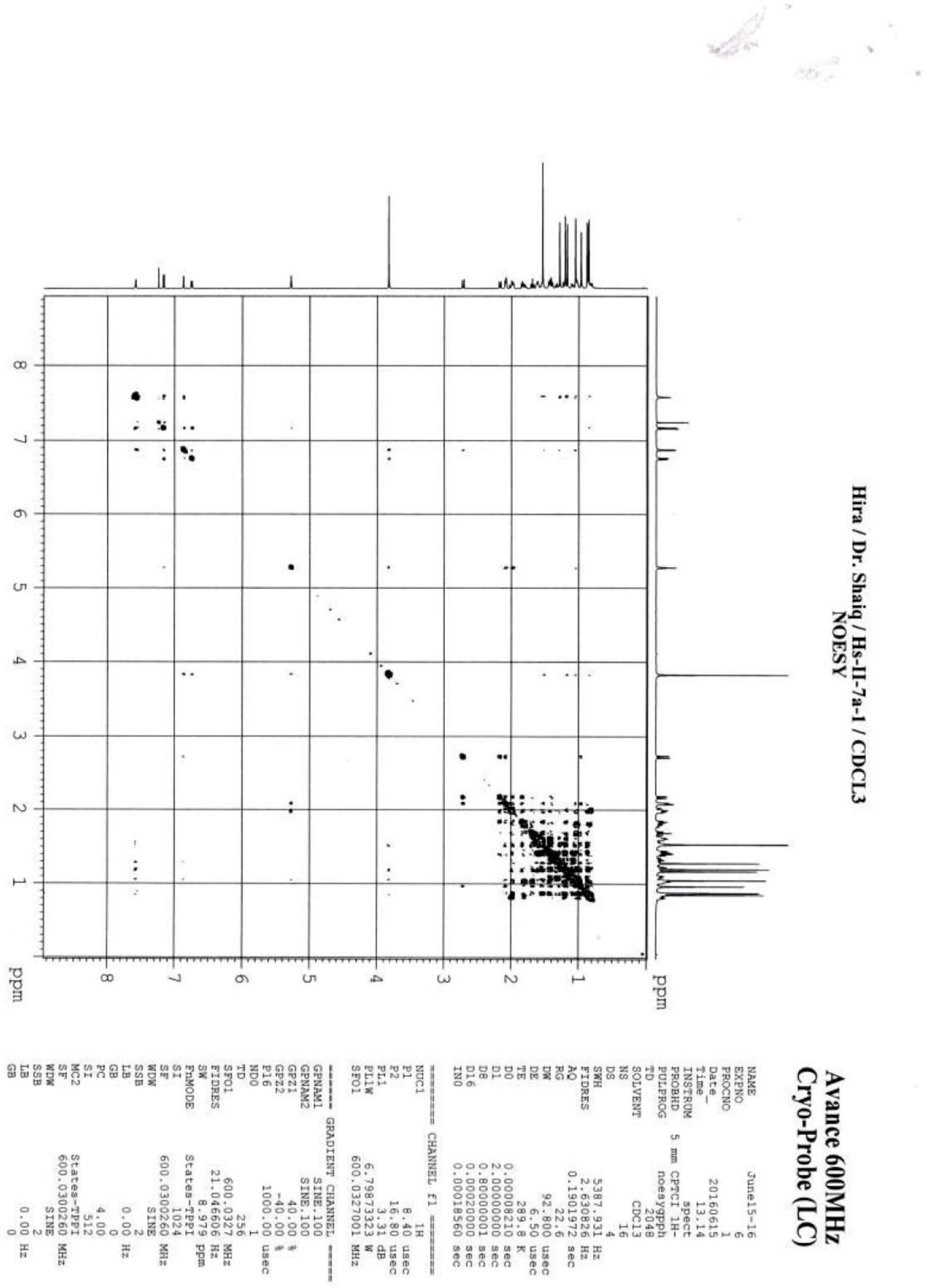

S22: NOESY spectrum of compound **3**

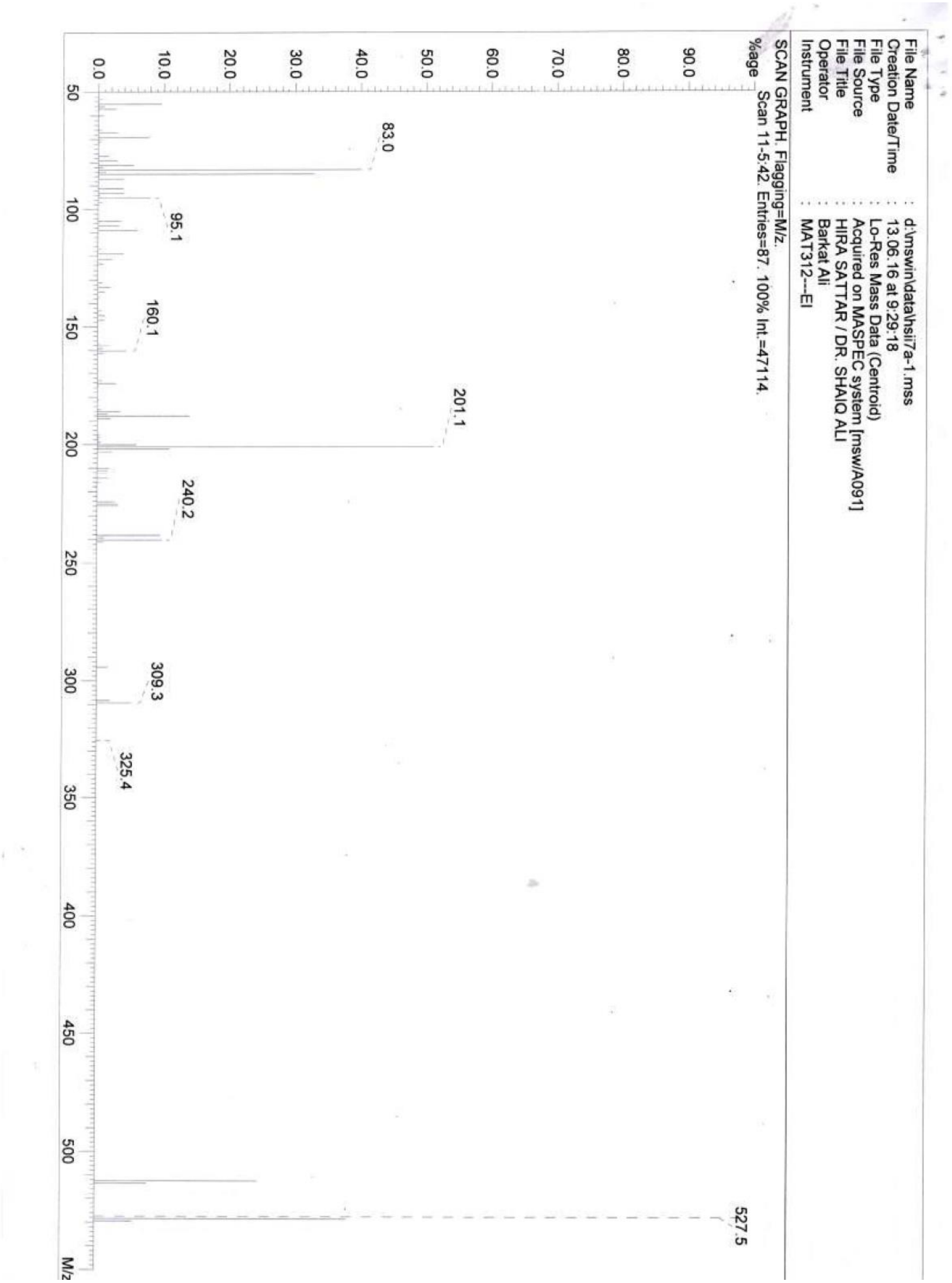

S23: EIMS spectrum of compound **3**

Mass	Relative Intensity	Theoretical Mass	Delta [ppm]	Delta [mmu]	RDB	Composition
513.40852	3.1	513.4096	-2.2	-1.1	11.5	$C_{37} H_{53} O_{1}$
		513.3971	22.3	11.5	12.0	$C_{36} H_{51} O_{1} N_{1}$
514.39790	1.7	514.4049	-13.6	-7.0	11.5	$C_{36} H_{52} O_{1} N_{1}$
524.39430	1.2	524.3892	9.6	5.1	13.5	$C_{37} H_{50} O_{1} N_{1}$
		524.4018	-14.3	-7.5	13.0	$C_{38} H_{52} O_{1}$
525.40034	2.6	525.3971	6.2	3.3	13.0	$C_{37} H_{51} O_{1} N_{1}$
		525.4096	-17.7	-9.3	12.5	$C_{38} H_{53} O_{1}$
526.40275	2.3	526.4049	-4.1	-2.1	12.5	$C_{37} H_{52} O_{1} N_{1}$
		526.4175	-28.0	-14.7	12.0	$C_{38} H_{54} O_{1}$
527.40988	6.0	527.4127	-5.4	-2.8	12.0	$C_{37} H_{53} O_{1} N_{1}$
		527.4253	-29.2	-15.4	11.5	$C_{38} H_{55} O_{1}$
528.41511	2.4	528.4205	-10.3	-5.4	11.5	$C_{37} H_{54} O_{1} N_{1}$
529.41378	1.1	529.4046	17.4	9.2	11.5	$C_{37} H_{53} O_{2}$
		529.4284	-27.5	-14.6	11.0	$C_{37} H_{55} O_{1} N_{1}$
541.42645	1.6	541.4284	-3.5	-1.9	12.0	$C_{38} H_{55} O_{1} N_{1}$
		541.4409	-26.8	-14.5	11.5	$C_{39} H_{57} O_{1}$

S24: HR-EIMS spectrum of compound **3**

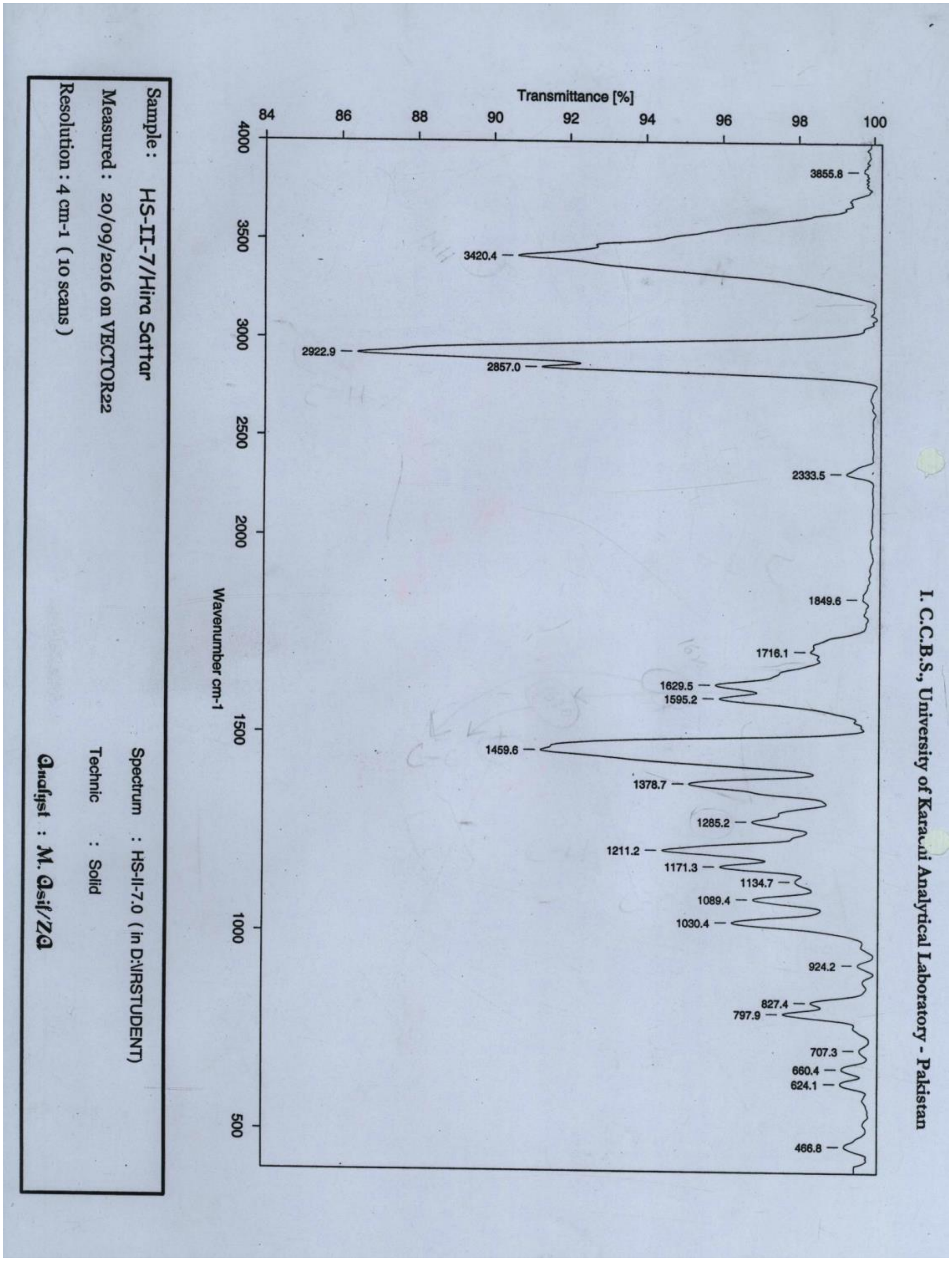

S25: IR spectrum of compound **3**

THERMO ELECTRON ~ VISIONpro SOFTWARE V4.10

Operator Name	ARSHAD ALAM	Date of Report	4/27/2018
Department	Analytical Laboratory TWC # 004	Time of Report	12:32:22AM
Organization	ICCBS Karachi of Universty.		
Information	Prof.Dr.M.Shaiq Ali./ Hira Sattar.		

Scan Graph

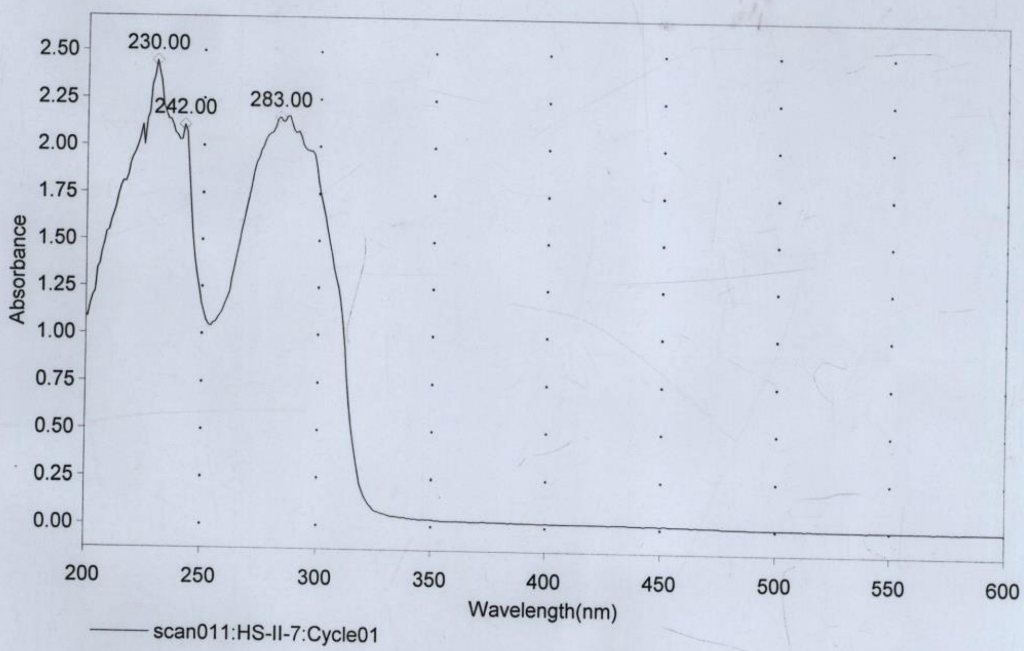

Results Table - HS-II-7.sre,HS-II-7,Cycle01

nm	A
230.00	2.446
242.00	2.109
283.00	2.154

Sensitivity Medium

Peak Pick Method
Find 3 Peaks Above -3.0000 A
Start Wavelength 200.00 nm
Stop Wavelength 600.00 nm
Sort By Wavelength

S26: UV spectrum of compound **3**

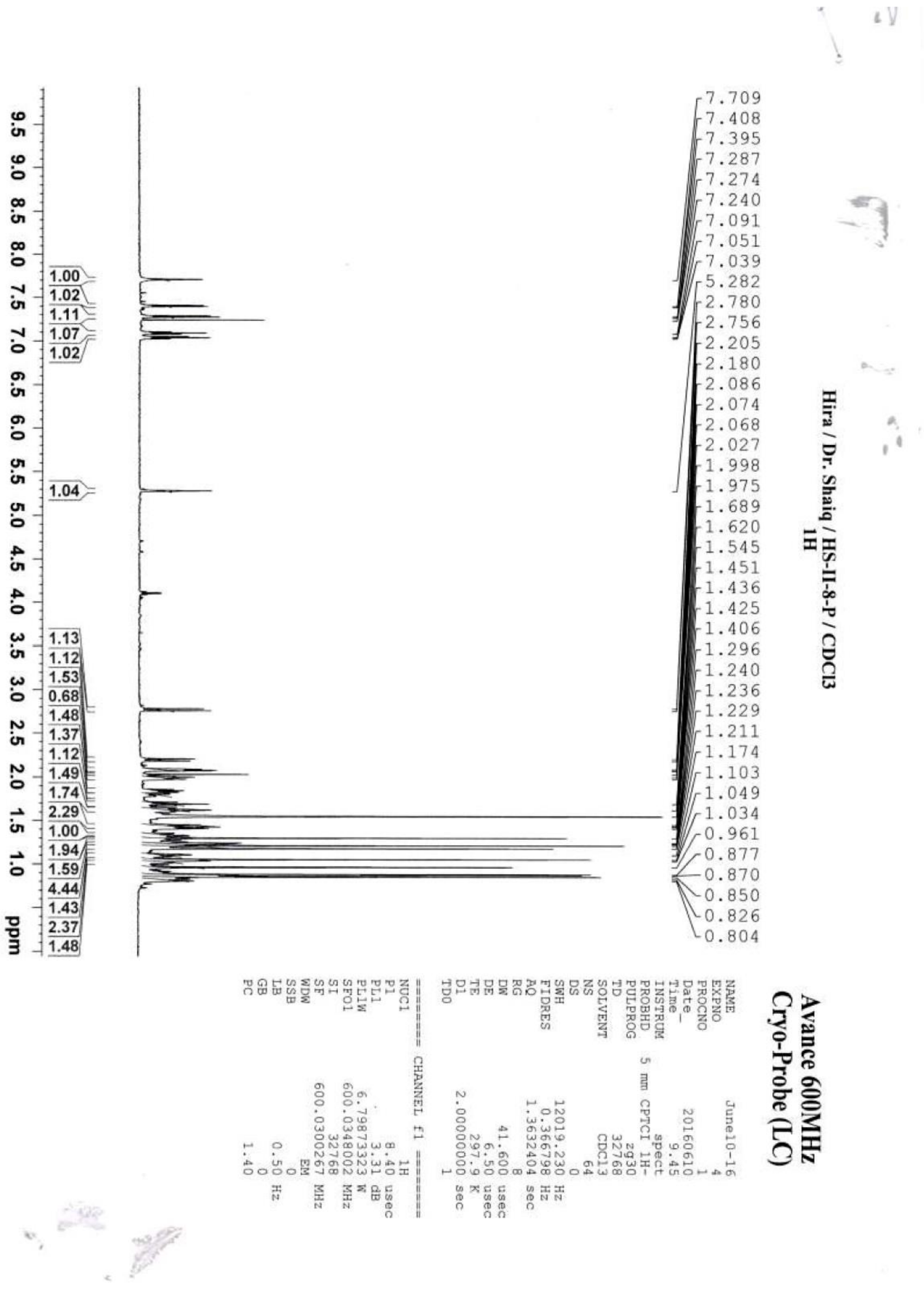
Hira / Dr. Shaiq / HS-II-8-P / CDCl3
1H
7.709
7.408
7.395
7.287
7.274
7.240
7.091
7.051
7.039
5.282
2.780
2.756
2.205
2.180
2.086
2.074
2.068
2.027
1.998
1.975
1.689
1.620
1.545
1.451
1.436
1.425
1.406
1.296
1.240
1.236
1.229
1.211
1.174
1.103
1.049
1.034
0.961
0.877
0.870
0.850
0.826
0.804
Avance 600MHz
Cryo-Probe (LC)
NAME June10-16
EXPNO 4
PROCNO 1
Date_ 20160610
Time 9.45
INSTRUM spect
PROBHD 5 mm CPTCI 1H-
PULPROG zg30
TD 32768
SOLVENT CDCl3
NS 64
DS 0
SWH 12019.230 Hz
FIDRES 0.366798 Hz
AQ 1.3632404 sec
RG 8
DW 41.600 usec
DE 6.50 usec
TE 297.9 K
D1 2.00000000 sec
TD0 1
======== CHANNEL f1 ========
NUC1 1H
P1 8.40 usec
PL1 3.31 dB
PL1W 6.79873323 W
SFO1 600.0348002 MHz
SI 32768
SF 600.0300267 MHz
WDW EM
SSB 0
LB 0.50 Hz
GB 0
PC 1.40
1.00
1.02
1.11
1.07
1.02
1.04
1.13
1.12
1.53
0.68
1.48
1.37
1.12
1.49
1.74
2.29
1.00
1.94
1.59
4.44
1.43
2.37
1.48
9.5 9.0 8.5 8.0 7.5 7.0 6.5 6.0 5.5 5.0 4.5 4.0 3.5 3.0 2.5 2.0 1.5 1.0 ppm

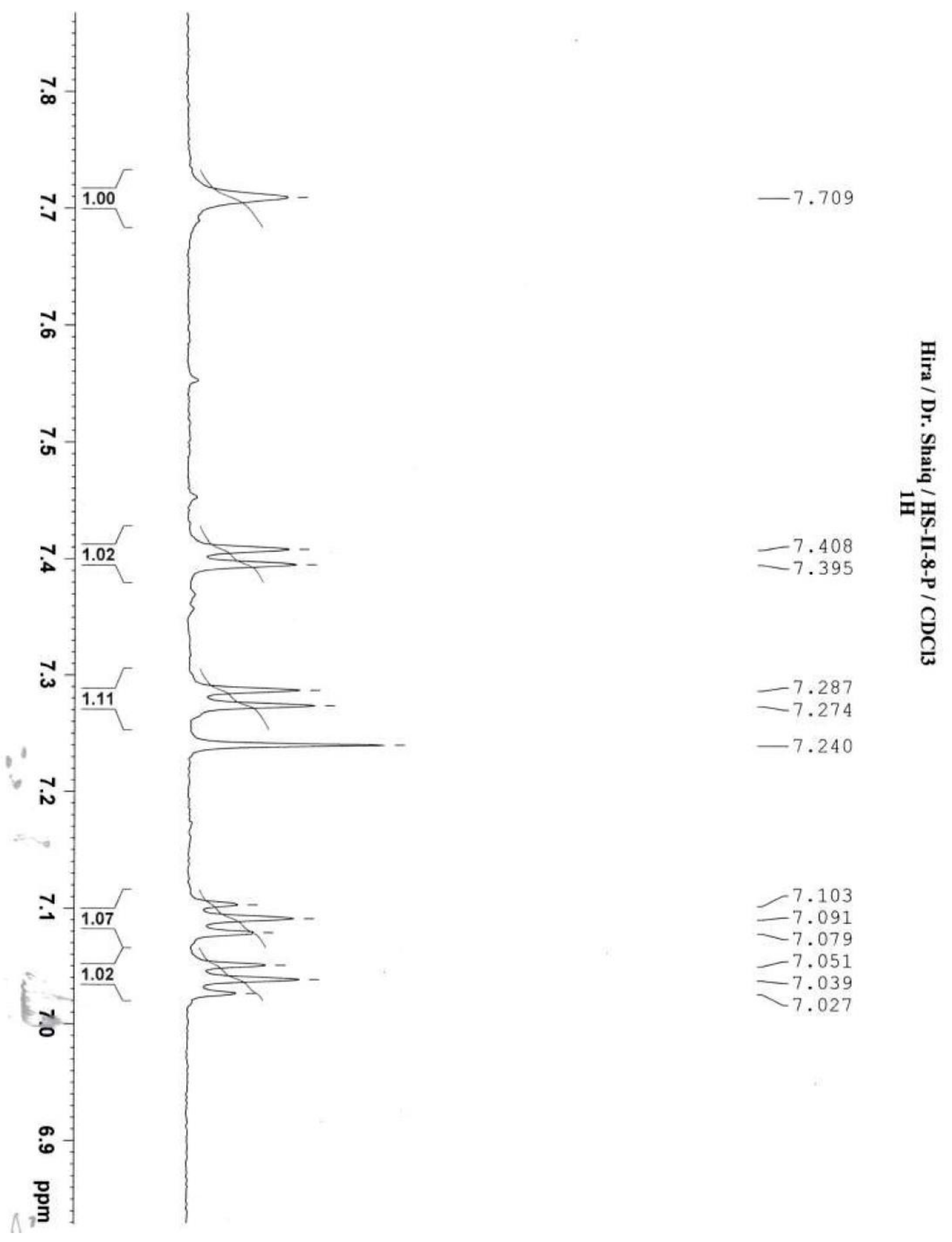

Hira / Dr. Shaiq / HS-II-8-P / CDCl3
1H
7.709
7.408
7.395
7.287
7.274
7.240
7.103
7.091
7.079
7.051
7.039
7.027
1.00
1.02
1.11
1.07
1.02
7.8
7.7
7.6
7.5
7.4
7.3
7.2
7.1
7.0
6.9
ppm

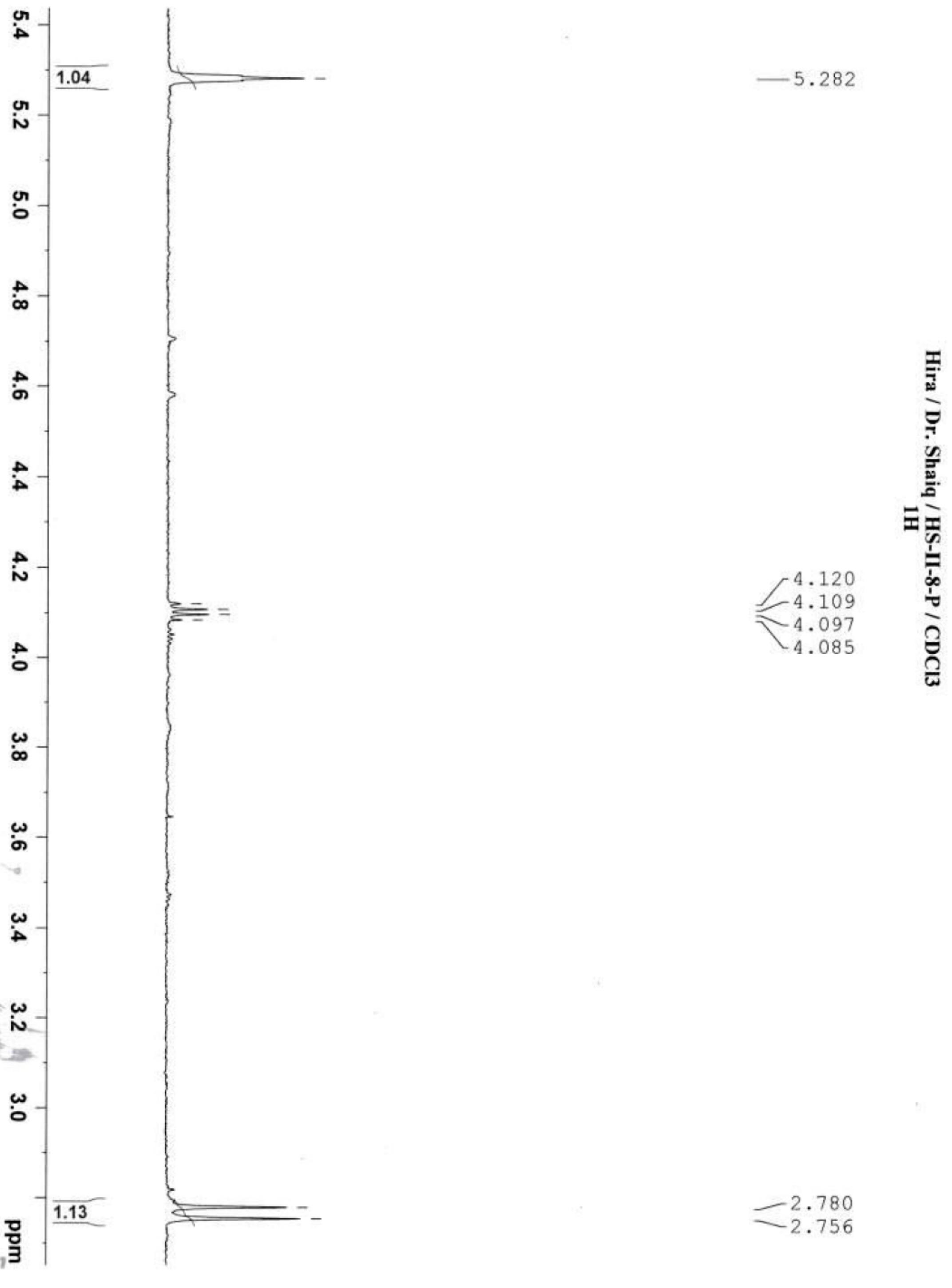
Hira / Dr. Shaiq / HS-II-8-P / CDCl3
1H
5.282
4.120
4.109
4.097
4.085
2.780
2.756
1.04
1.13
5.4
5.2
5.0
4.8
4.6
4.4
4.2
4.0
3.8
3.6
3.4
3.2
3.0
ppm

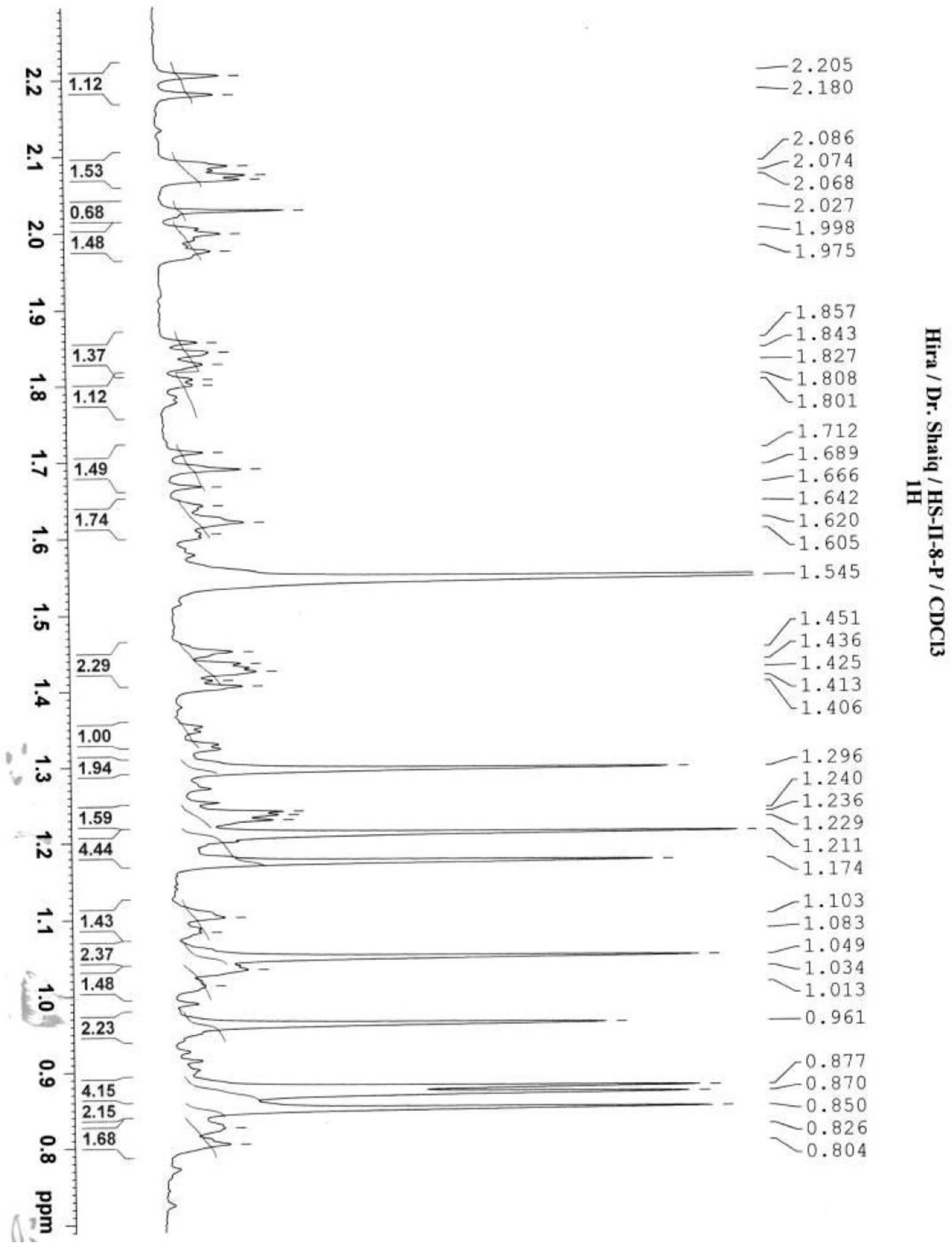

S27: ^{1}H-NMR spectrum of compound **4**

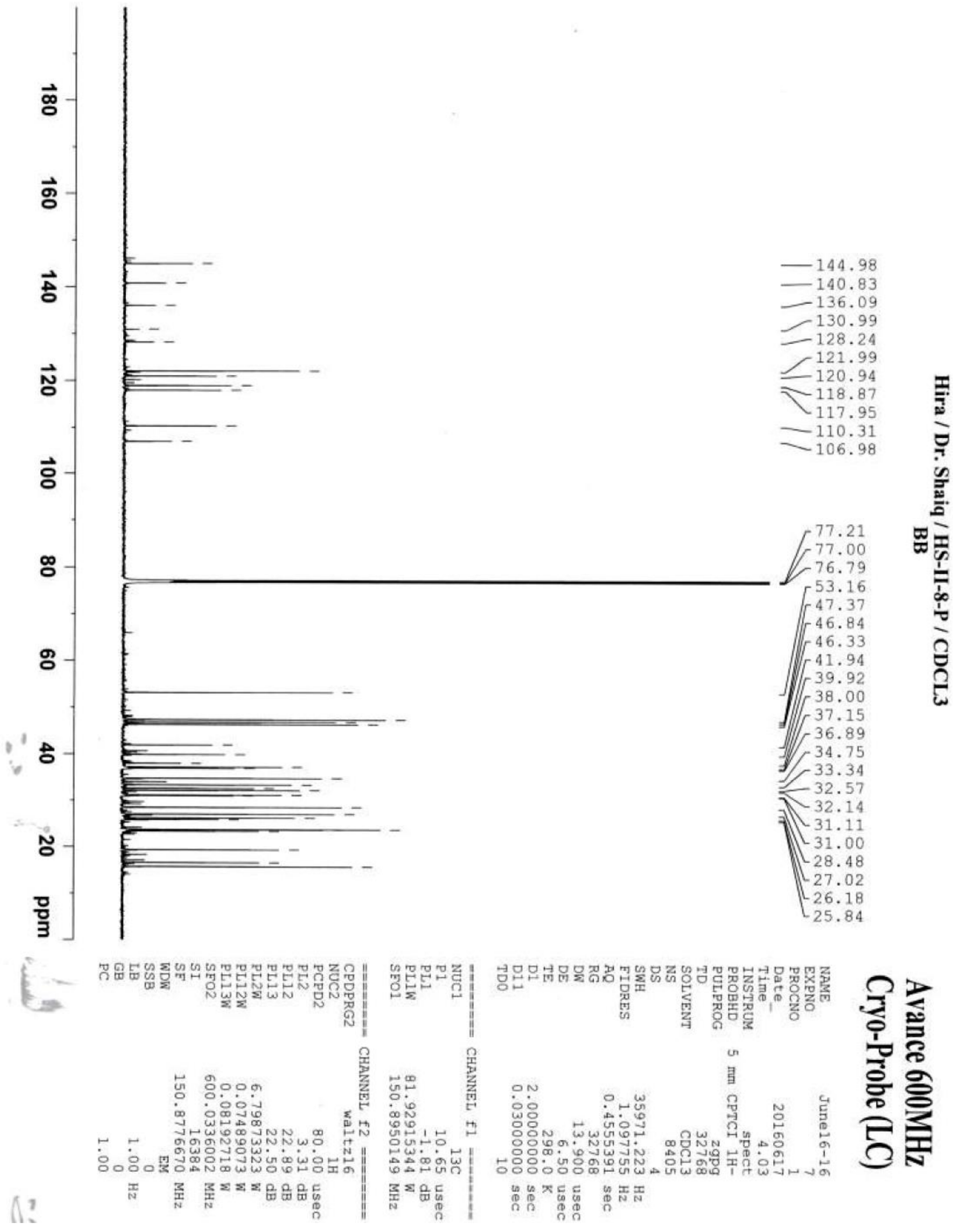

S28: ^{13}C-NMR spectrum of compound **4**

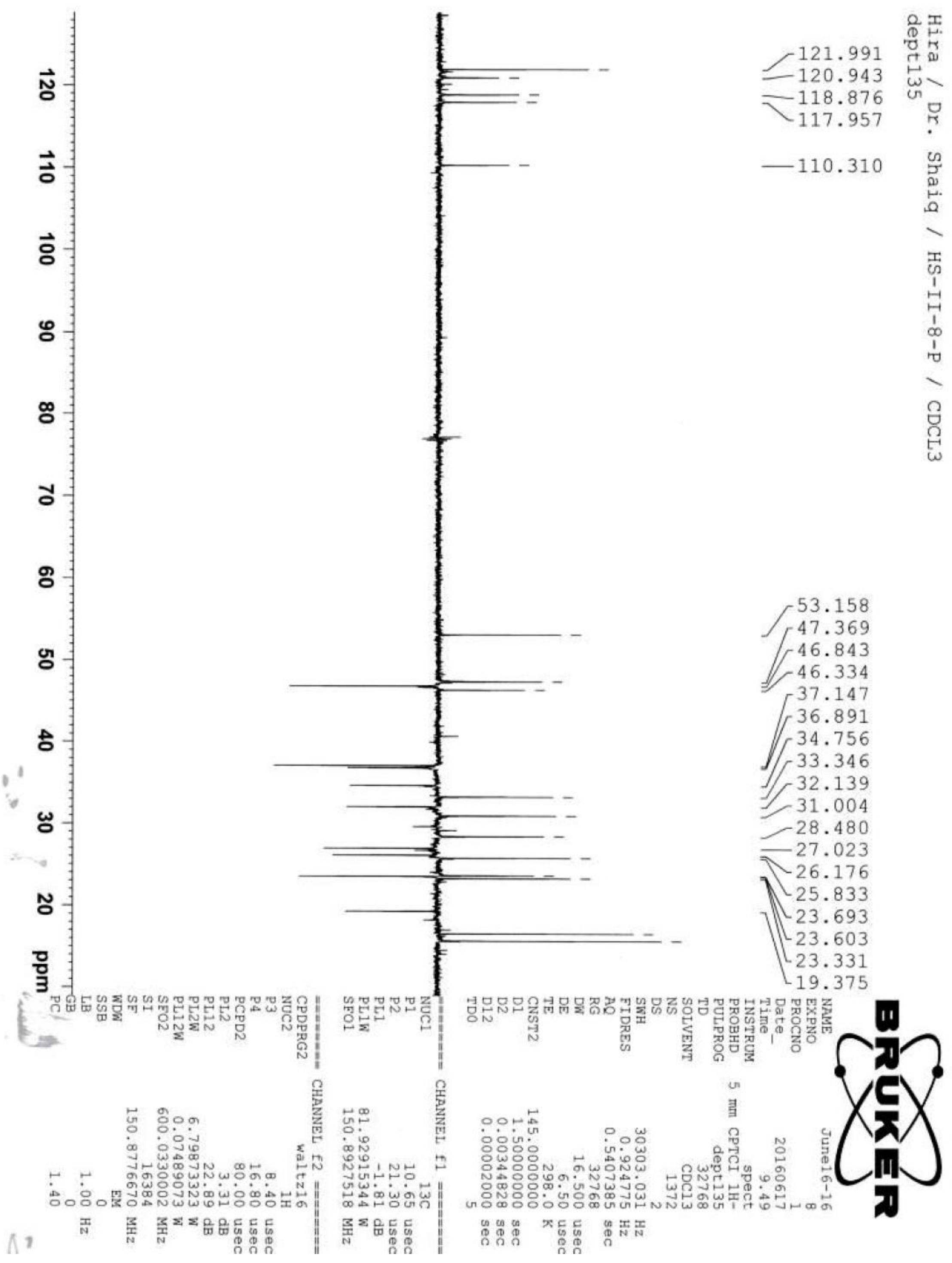

S29: ^{13}C DEPT-135 spectrum of compound **4**

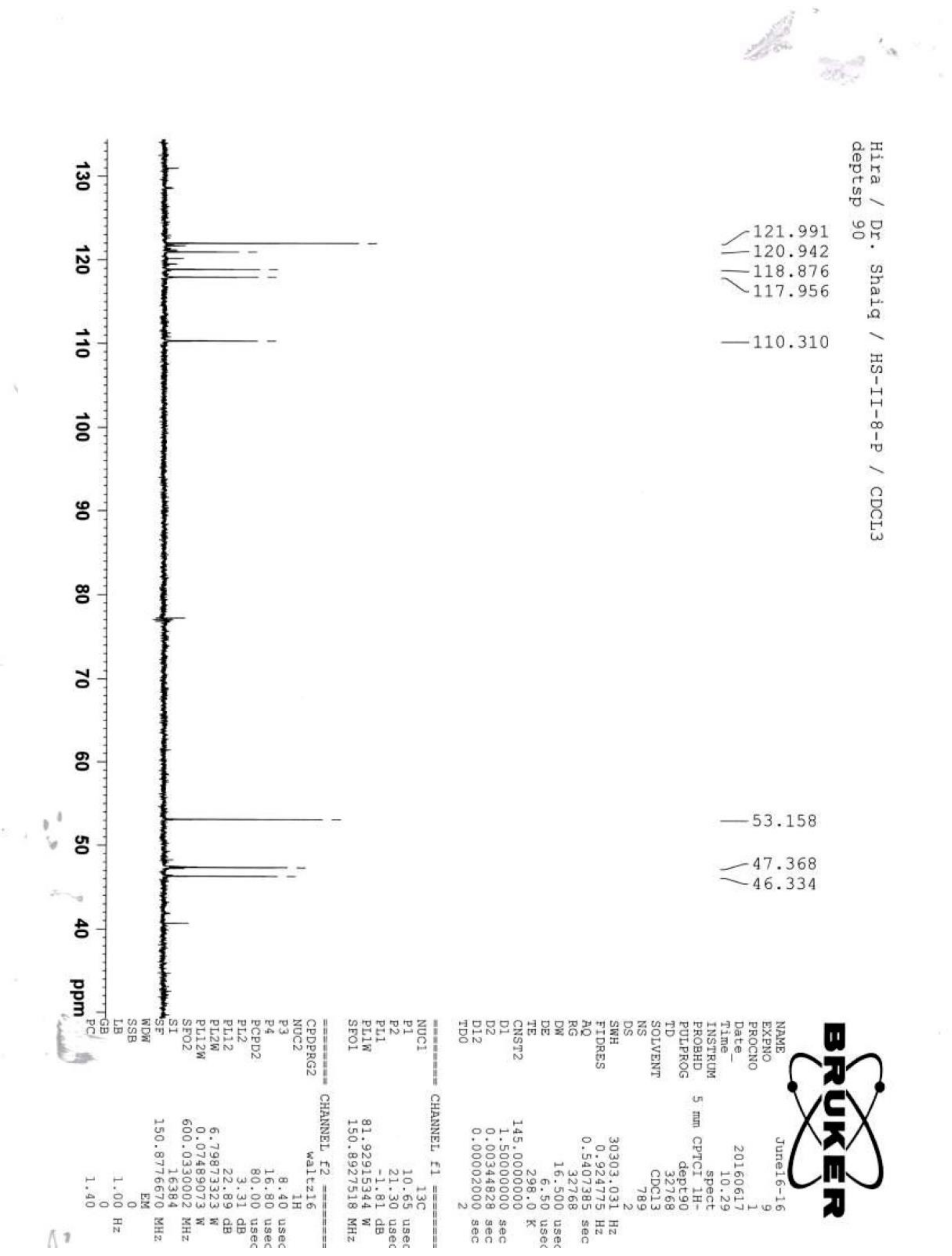

S30: ^{13}C DEPT-90 spectrum of compound **4**

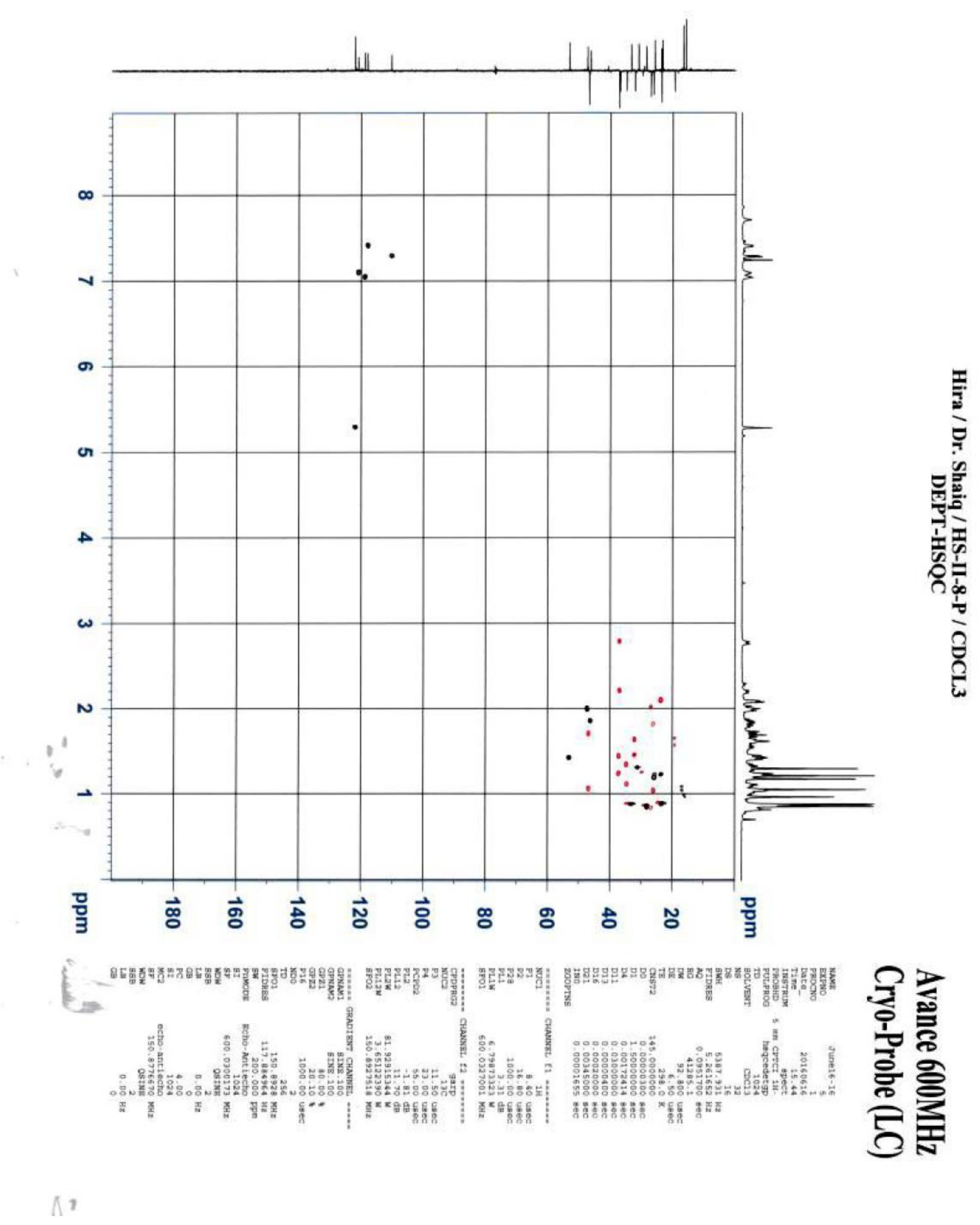

S31: DEPT-HSQC spectrum of compound **4**

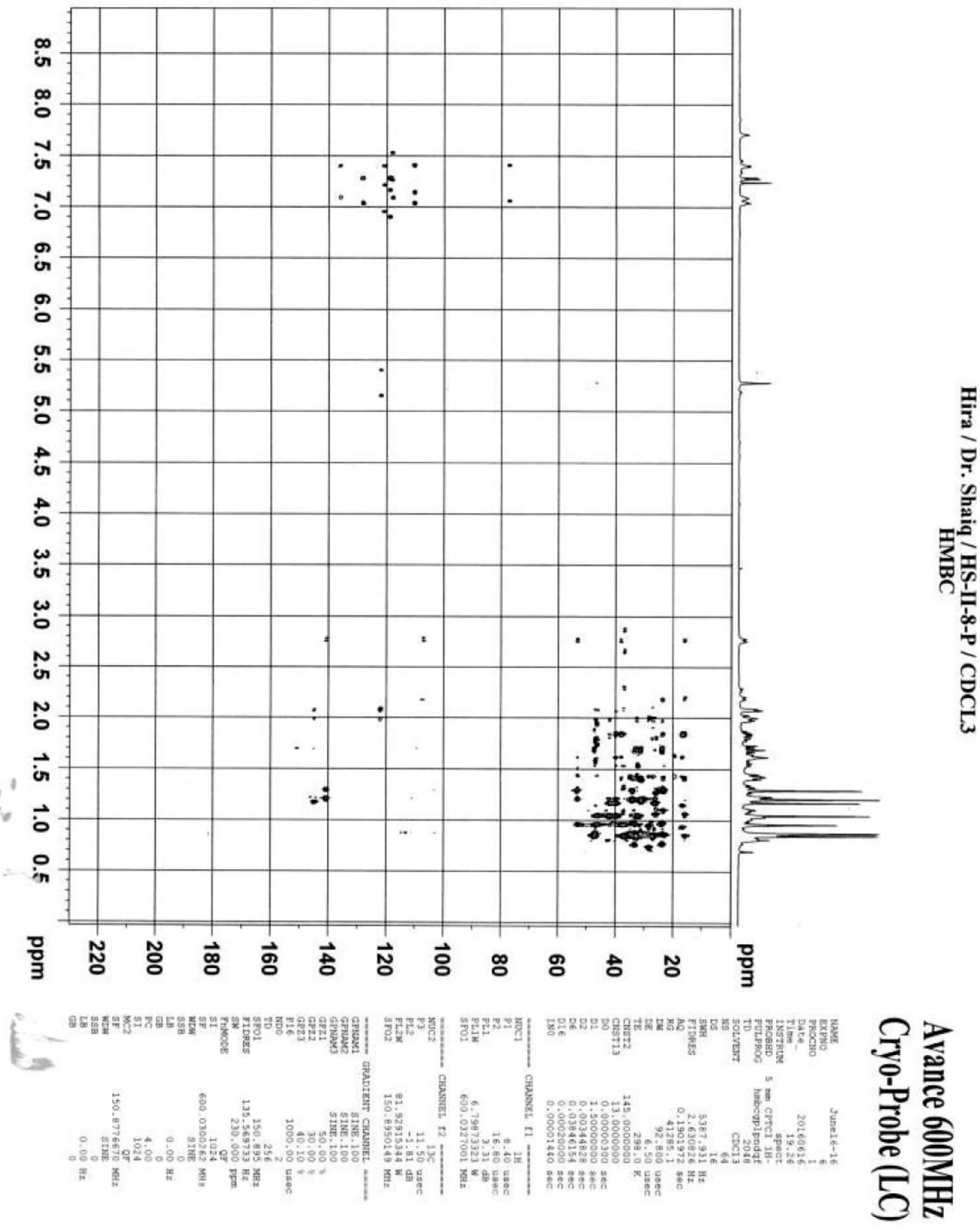

S32: HMBC spectrum of compound **4**

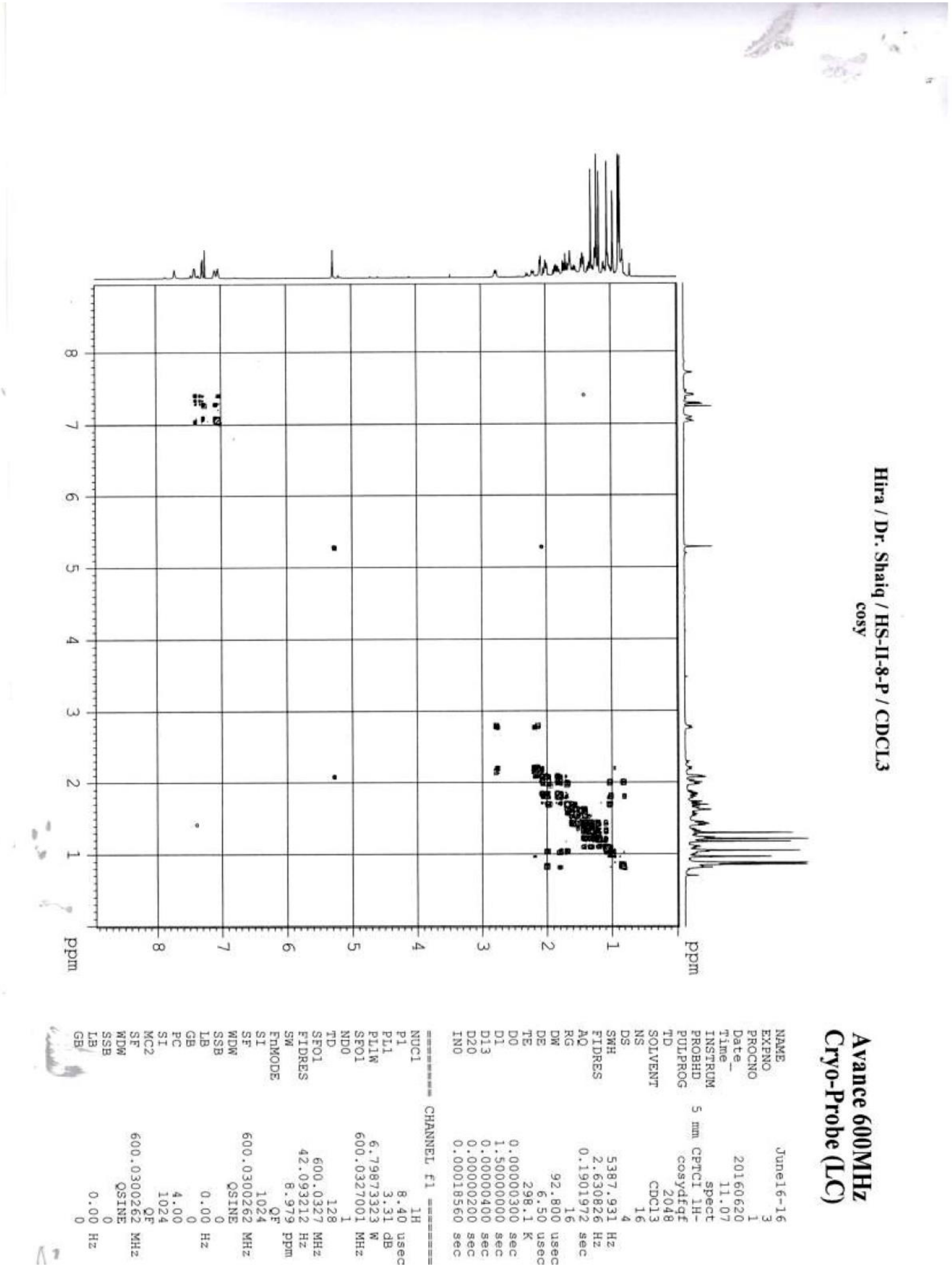

S33: COSY spectrum of compound **4**

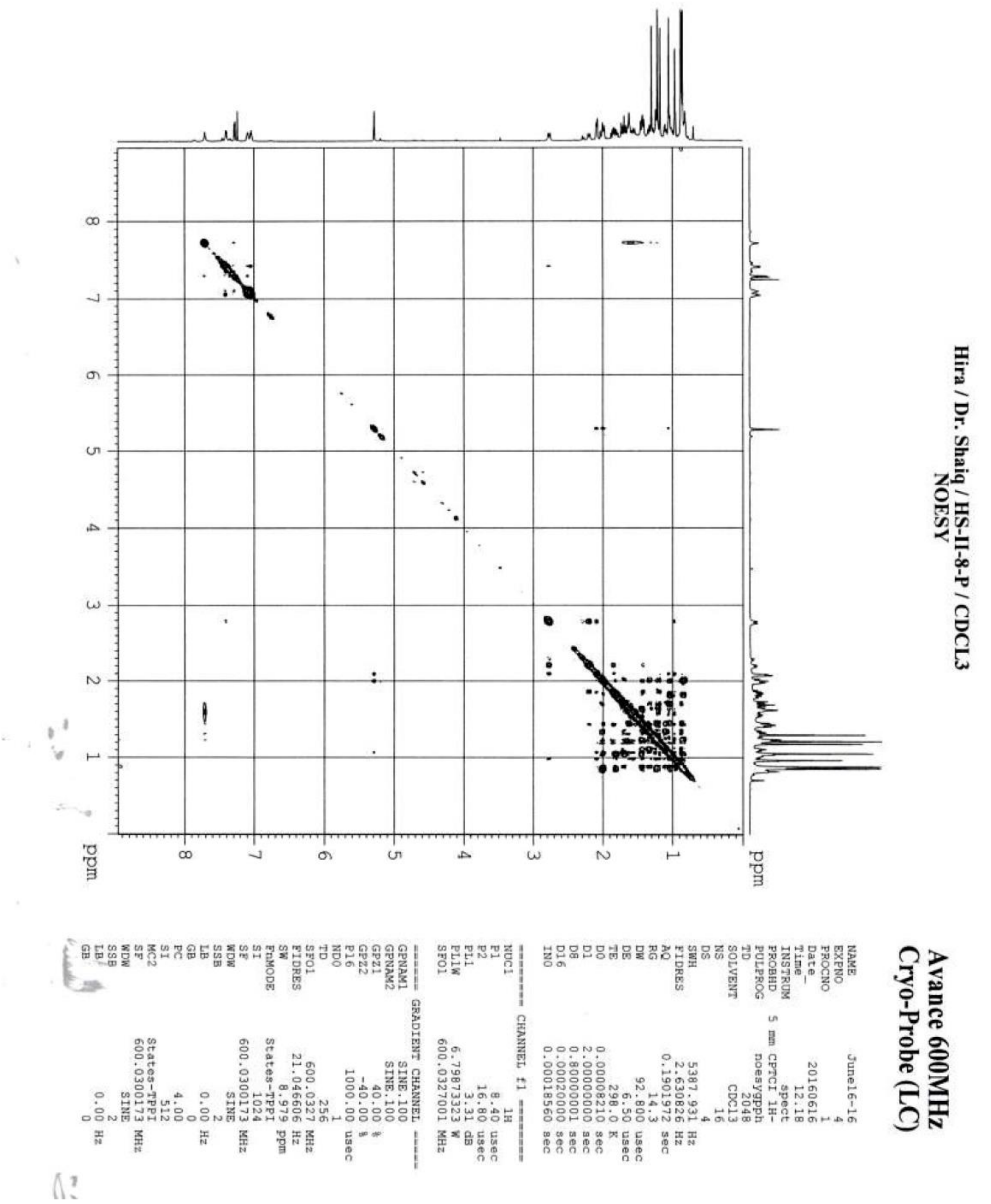

S34: NOESY spectrum of compound **4**

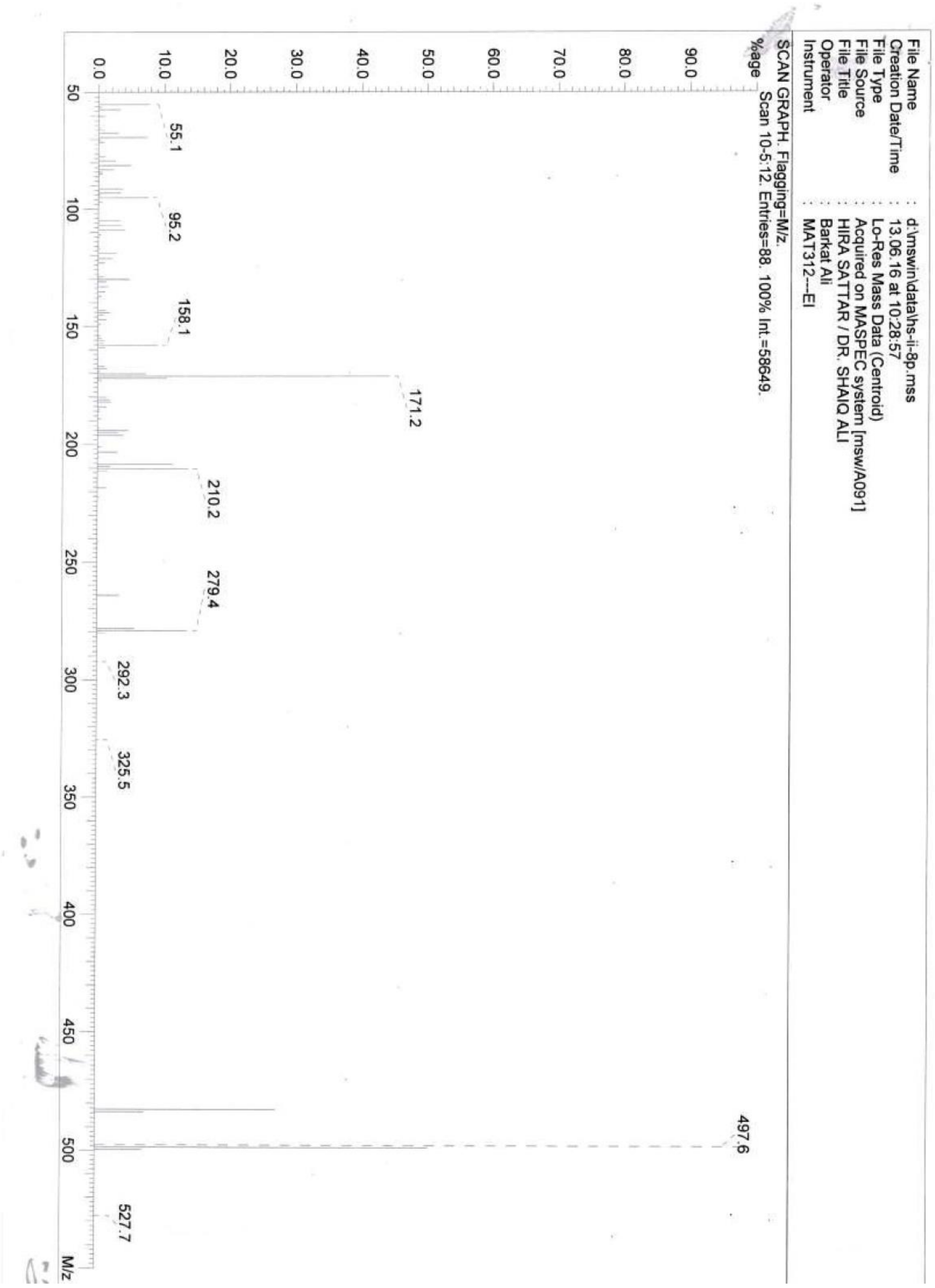

S35: EIMS spectrum of compound **4**

Mass	Relative Intensity	Theoretical Mass	Delta [ppm]	Delta [mmu]	RDB	Composition
		483.3991	-31.4	-15.2	11.5	$C_{36}H_{51}$
497.39929	4.6	497.4022	-5.8	-2.9	12.0	$C_{36}H_{51}N_1$
		497.4147	-31.0	-15.4	11.5	$C_{37}H_{53}$
498.40664	2.0	498.4100	-6.7	-3.3	11.5	$C_{36}H_{52}N_1$
		498.4226	-31.9	-15.9	11.0	$C_{37}H_{54}$
499.40256	0.4	499.4178	-30.5	-15.2	11.0	$C_{36}H_{53}N_1$

S36: HR-EIMS spectrum of compound **4**

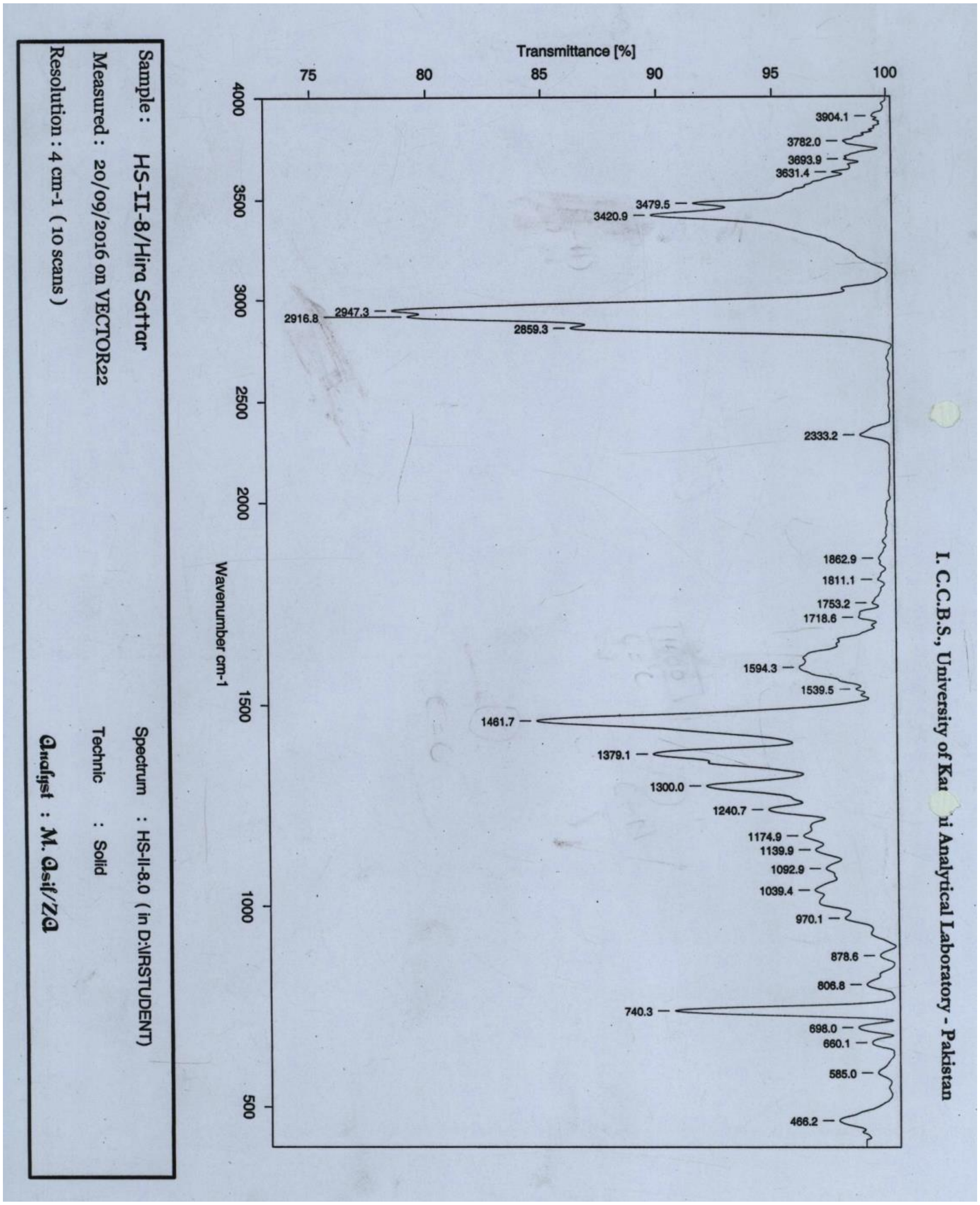

S37: IR spectrum of compound **4**

THERMO ELECTRON ~ VISIONpro SOFTWARE V4.10

Operator Name	ARSHAD ALAM	Date of Report	4/27/2018
Department	Analytical Laboratory TWC # 004	Time of Report	12:39:36AM
Organization	ICCBS Karachi of Universty.		
Information	Prof.Dr.M.Shaiq Ali./ Hira Sattar.		

Scan Graph

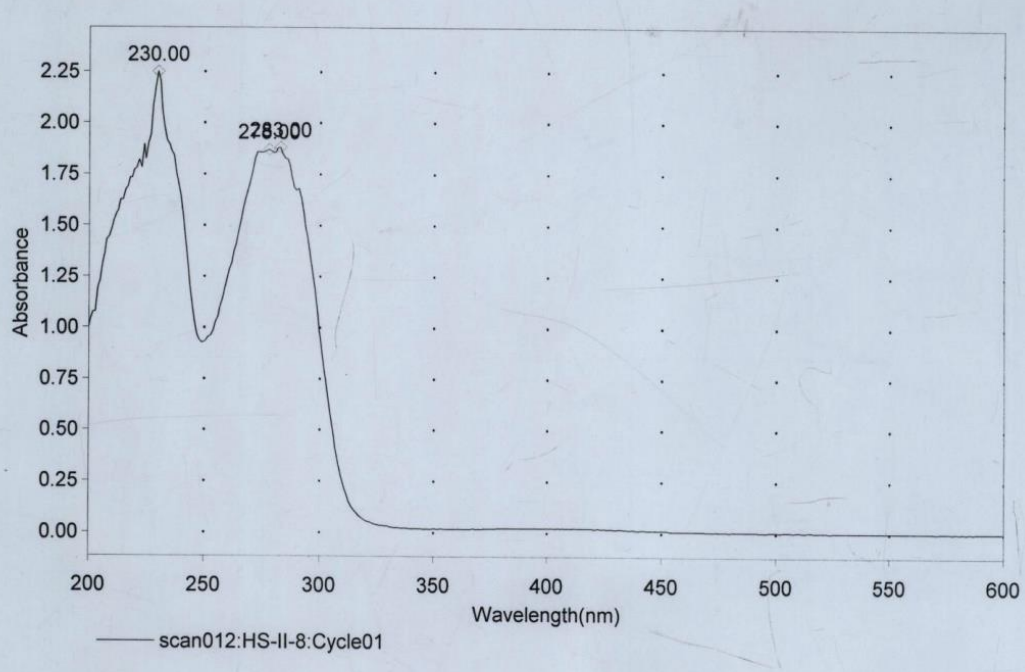

Results Table - HS-II-8.sre,HS-II-8,Cycle01

nm	A	**Peak Pick Method**
230.00	2.248	Find 3 Peaks Above -3.0000 A
278.00	1.871	Start Wavelength 200.00 nm
283.00	1.880	Stop Wavelength 600.00 nm
		Sort By Wavelength
Sensitivity	Medium	

S38: UV spectrum of compound **4**
